# GeoSPM: Geostatistical parametric mapping for medicine

**DOI:** 10.1016/j.patter.2022.100656

**Published:** 2022-12-09

**Authors:** Holger Engleitner, Ashwani Jha, Marta Suarez Pinilla, Amy Nelson, Daniel Herron, Geraint Rees, Karl Friston, Martin Rossor, Parashkev Nachev

**Affiliations:** 1 UCL Queen Square Institute of Neurology, University College London, London WC1N 3BG, UK; 2 Research & Development, NIHR University College London Hospitals Biomedical Research Centre, London W1T 7DN, UK

**Keywords:** geostatistics, spatial analysis, statistical parametric mapping, kriging, epidemiology

## Abstract

The characteristics and determinants of health and disease are often organized in space, reflecting our spatially extended nature. Understanding the influence of such factors requires models capable of capturing spatial relations. Drawing on statistical parametric mapping, a framework for topological inference well established in the realm of neuroimaging, we propose and validate an approach to the spatial analysis of diverse clinical data—GeoSPM—based on differential geometry and random field theory. We evaluate GeoSPM across an extensive array of synthetic simulations encompassing diverse spatial relationships, sampling, and corruption by noise, and demonstrate its application on large-scale data from UK Biobank. GeoSPM is readily interpretable, can be implemented with ease by non-specialists, enables flexible modeling of complex spatial relations, exhibits robustness to noise and under-sampling, offers principled criteria of statistical significance, and is through computational efficiency readily scalable to large datasets. We provide a complete, open-source software implementation.

## Highlights

• A framework for topological inference applicable to diverse clinical data is proposed
• Superior robustness to noise and under-sampling is observed compared with kriging
• Application to UK Biobank data is demonstrated

## The bigger picture

Many aspects of health and disease are distributed in space, requiring models of topological organization. The complexity of the task, however, makes spatial analysis comparatively rare in medicine. Here, we introduce GeoSPM, a platform for topological inference from clinical data based on a mature mathematical framework—statistical parametric mapping—validated by decades of use in neuroimaging. We provide comprehensive synthetic evaluation of the approach, and illustrate its application on large-scale data from UK Biobank. The interpretability, flexibility, scalability, ease of implementation, robustness to noise and under-sampling, computational efficiency, and provision of principled criteria of statistical significance, provided by our open-source platform should catalyze wider use of spatial analysis across medicine.

## Teaser

We present GeoSPM, an approach to the spatial analysis of diverse clinical data that extends a framework for topological inference, well established in neuroimaging, based on differential geometry and random field theory. We evaluate GeoSPM with extensive synthetic simulations, and apply it to large-scale data from UK Biobank. Our approach is readily interpretable, easy to implement, enables flexible modeling of complex spatial relations, exhibits robustness to noise and under-sampling, offers principled criteria of statistical significance, and is scalable to large datasets.

## Introduction

Human beings vary along a rich multiplicity of social and biological dimensions, whose complex interactions across health and disease present a challenge for medical science and systems biology in general. The combination of large-scale data with machine learning promises to cast brighter light on this complexity than conventional inferential techniques, illuminating distributed, long-range dependencies hitherto obscured. Our interventions are increasingly grounded in an understanding of the factors that shape disease trajectories and determine individual responses to treatment.

One comparatively neglected dimension is the literal dimension of space: each of us inhabits a particular location that may reflect or modify our individual biological characteristics and the influence of (and on) other spatially distributed variables. Spatial factors may be static or vary over time, arising at multiple scales, ranging from the domestic to the inter-continental. Their reference frames may be set by internal communities, by external geographies, or by a complex blend of the two. Their spatial organization may be linear or consistently distorted by individual or environmental movement within these frames of reference. Spatial factors may disclose or alter characteristics of biology directly, or render them more or less clinically accessible or actionable. Space arises not only in epidemiology, environmental medicine, healthcare policy, and public health, but in the fundamental organization of biology itself.

Yet outside a few specialist areas spatial analysis is comparatively rare in medicine. An indicative survey of published paper titles and abstracts in Microsoft Academic Graph, spanning 30 years of medical research, reveals only 1,897 journal papers at the intersection of geospatial analysis and medicine, with an annual citation distribution for those cited more than once nonetheless substantially higher than a matched biomedical sample (mean 2.75 versus 2.13, Mann-Whitney U test, p < 0.001, Figure S1, see supplemental note). The comparative scarcity is arguably in part explained by the difficulty of the task. The spatial factors arising in a medical context are often entangled, their sampling is sparse and frequently corrupted by noise, and the underlying signals tend to be weak. But spatial analysis is hard even where the data regime is benign, for the problem is essentially multidimensional and is rarely, if ever, open to analytic solutions.

The fundamental challenge is reflected in the wide array of techniques in current use. A survey of 397 papers published since January 1, 2017, in the joint domains of health and spatial modeling identifies local indicators of spatial association,^1^ spatial scan statistics,^2^ inverse distance weighting,^3^ kernel density estimation,^4^^,^^5^ spatial regression in terms of spatial lag and spatial error models,^6^ geographically weighted regression (GWR),^7^ land-use regression,^8^ kriging,^9^^,^^10^ generalized linear mixed models,^11^ generalized (geo-)additive models,^12^^,^^13^ hierarchical Bayesian spatial analysis,^14^^,^^15^ and model-based geostatistics,^16^^,^^17^ among others.

This methodological diversity reflects differing demands on the spatial aspects of the model and the breadth of specific questions that arise in a spatial setting. With the question may vary the modeling objective, and the theoretical assumptions that underpin it. Common objectives include spatial prediction, the analysis and regression of spatially varying or spatially confounded associations, and the investigation of spatial point patterns. Arguably the most general and taxing research questions involve inference—whether explicit or not—to a topological organization, for example, identifying the location and extent of a spatially organized signal buried in noise. Such questions typically—if not always—require methods that treat space as a continuity, produce spatially continuous estimates, and provide principled measures of spatial uncertainty. Dominant in this category are methods that adopt a nonlinear multivariate approach, taking advantage of the flexibility and expressivity it offers. Although potentially powerful, they require joint expertise in the method and the domain of its application, depend on prior specification of model parameters, and tend to demand substantial computational resource even for data of moderate scale. Furthermore, in the generalized linear framework, space commonly enters the model as a latent random effect—usually derived from a suitable Gaussian process. This approach adjusts for spatially correlated variance within an otherwise non-spatial framework, with the fixed effects remaining constant across the spatial field.^18^

These obstacles motivate the pursuit of alternatives outside the multivariate paradigm for the task of topological inference. The direct counterpoint is a mass-univariate approach, where a complex multivariate model is replaced by a spatially indexed ensemble of simpler models. GWR modifies the predictors in a regression model through a spatially localized weight matrix, so that a variation of the model is estimated at each location and the resulting estimates exhibit spatial smoothness. Although GWR estimates can be derived from a prespecified grid, in practice only sampled locations or grids of modest size tend to be evaluated owing to the difficulty of correcting for multiple comparisons in a topologically informed manner.^19^ Spatial inference with GWR is commonly limited to regression coefficient or coefficient of determination maps that simply indicate the local goodness of fit,^20^ without employing formal tests of significance.^21^^,^^22^ Finally, these are regression models relating a response to a set of spatially organized predictors, not models of the spatial variation of a set of variables within a topological framework of uncertainty: our primary concern.

Here, we propose, implement, and validate an approach to the spatial analysis of diverse clinical or public health data that draw upon differential geometry and random field theory, with the topological objective of identifying connected neighborhoods and peaks of spatial significance. In particular, we leverage the procedures used in statistical parametric mapping (SPM): a framework for making topological inferences about spatially structured effects, with well-behaved spatial dependencies.^23^ This approach has been established for decades in the realm of (structural and functional) volumetric neuroimaging.

The core idea is to transform sparse spatial signals into a form suited to mass-univariate statistical testing on a chosen point grid: for example, testing that the spatial or regional expression of a particular variable is greater than would be expected under the null hypothesis of no regional effect. The probability of observing topological features in the observed map, such as peaks or clusters (i.e., level sets above some threshold), can then be evaluated with classical inference based on random field theory, and used to ascribe a p value to spatially organized effects. This principled approach radically simplifies one important domain of spatial analysis, rendering it potentially more sensitive and robust to noise, and places it on a formal inferential footing, yielding a general-purpose geostatistical tool readily deployable across a multitude of medical fields where the modeling objective requires inference to the topological organization of a set of signals of interest. For example, we may use the approach to infer the location and extent of regional expression of spatially organized variables—taken alone or in conjunction—such as disease prevalence in a community, while accounting for multiple potentially interacting confounding factors, and without relying on any *a priori* parcellation of the space.

In what follows, we (1) offer a detailed rationale for our approach; (2) proceed to evaluate it across an extensive array of synthetic simulations where the nature of the spatial relationships, sampling, and corruption by noise are prespecified; and (3) demonstrate its application on large-scale data from UK Biobank (https://www.ukbiobank.ac.uk/).^24^ The numerical analyses serve to establish face validity; the empirical analysis to demonstrate predictive validity. We provide a complete, open-source software implementation of our framework (https://github.com/high-dimensional/geospm), released as an extension to SPM; namely, geospatial SPM or “GeoSPM.” Supplemental note S4 and Figure S56 provide an overview of GeoSPM’s class structure as implemented in MATLAB.

## Experimental procedures

### Resource availability

#### Lead contact

Further information and requests for resources should be directed to and will be fulfilled by the lead contact, Holger Engleitner (h.engleitner@ucl.ac.uk).

#### Materials availability

This study did not generate new unique reagents and did not use any additional materials aside from the data and code cited below.

### Overview

Our approach builds on the well-established regression analysis framework implemented in SPM12 (http://www.fil.ion.ucl.ac.uk/spm/), the most widely used platform for spatial inference in brain imaging. Within this framework, a set of explanatory variables is associated with a multivariate, spatially structured response, whose components represent measurements taken at regular locations in a spatial domain. The association between explanatory variables and response is estimated at each location separately, using the same general linear model (GLM). This yields a collection of univariate multiple regression models that share the same model architecture and design matrix but differ in the response variable and the estimated parameter values. Crucially, random fluctuation, or variations in the response variable that are not explained by the GLM, are treated as realizations of a random (spatial) field with certain contiguity or smoothness properties. This is *mass-univariate* inference from a spatial perspective.

A distinguishing feature of SPM is the manner of correcting for multiple comparisons when testing mass-univariate model parameters (i.e., regression coefficients) for significance. The large number of tests, performed simultaneously, gives rise to a proportionally large number of false positives by chance alone. Conversely, the strong spatial correlations among the components of the response violate assumptions of mutual independence, and render simple Bonferroni correction inappropriately strict. SPM applies a more suitable correction by modeling the residuals as a random Gaussian field, so that p values are meaningful in terms of identifying significant peaks and clusters in a discretized spatial domain. Heuristically, topological inference of this kind automatically accounts for spatial dependencies; in the sense that smooth random fluctuations will produce a smaller number of maxima than rough random fields with less spatial dependence (even though the total area above some threshold could be the same). It can be shown that the smoothness of the residual fields is a suitable approximation to the smoothness of a t statistic map derived from the model, which in turn reflects the spatial dependence of the covariates.^25^^,^^26^

The kind of data we are concerned with comprise variables of interest observed at locations in a continuous spatial domain D. D is usually a subset of $R^{2}\;$ representing coordinates of a geographic space. More precisely, every element in a spatially referenced dataset associates a vector $y_{i}\;$ of P variable observations ${\left(y_{i1},\ldots ,y_{iP}\right)}^{\text{T}}\in R^{P}$ with a location $x_{i}\in D$

$$\left(y_{i},x_{i}\right):i=1,\ldots ,N.$$

SPM typically requires data sampled at regular locations across a grid, spanning the spatial domain. However, we wish to analyze data that are irregularly and sometimes sparsely sampled. This can be resolved by distributing each data point locally—over regular grid locations—using a spatial Gaussian kernel of suitable and fixed variance.

From a data-centric point of view, we can interpret this spatial transformation as estimating the contribution of an individual observation to regular sample points, where the contribution has a maximum value at the observation location and then diminishes with increasing distance. In this way, the dependent variable in the univariate regression at any location of space is essentially a weighting of individual observations according to their proximity to that location: the higher the local response, the closer the observation. We can do this with impunity because we are interested in the explainable differences in these contributions at prespecified (grid point) locations. These explainable differences are assessed with normalized effect sizes (i.e., classical statistics), which are not affected by the total contribution or variance.^23^^,^^27^

The chosen variance of the Gaussian kernel is a parameter—hereafter called the *smoothing* parameter—deliberately left open to the analyst to specify the appropriate degree of spatial coarse graining (i.e., spatial smoothness of the data features in question). Since SPM naturally handles volumetric data, we are free to use the third dimension to model multiple smoothing values on a continuous positive scale, rendering them as different spatial “scales” or “features” of a response variable.^28^ Here, two coordinates represent the location in space (i.e., location space), and the third coordinate tracks spatial spread (i.e., scale space), allowing the regression analysis to operate at different scales simultaneously. It is appropriate to permit inference under varied assumptions of uncertainty, allowing the analyst to draw conclusions from the similarities and differences obtained across the range of plausible spatial scales. The analyst is also free to implement mechanisms that select an optimal parameter under some criterion: here we suggest one pragmatic method of doing this. Note that this scale-space implementation of topological inference automatically accounts for dependencies in moving from one scale to another and enables topological inference in terms of maxima or clusters in both location and scale space (i.e., a particular effect can be declared significant at this location and this spatial scale). For simplicity, we will focus on topological inference at a given spatial scale.

Downstream of the above spatial transformation of data features, the statistical approach is formally identical to a standard SPM analysis. The output comprises a series of volumes representing regression coefficients, statistical contrasts derived from these model parameters, the statistical parametric maps—of classical statistics based on these contrasts—and, finally, thresholded binary maps that indicate whether the voxels in the corresponding statistical map are significant at the chosen (suitably corrected) p value.

### Synthetic data and generative models

The statistical validity of the proposed approach is underwritten by the assumptions on which SPM rests. Nonetheless, it is helpful to examine its construct validity, in comparison with alternative methods (e.g., kriging), and face validity, in terms of its ability to recover known effects in different situations. Such validation is best performed with a known (spatial) ground truth, under manipulations of sampling and noise traversing the plausible space of possibility as far as is practicable. Note, however, that no aspect of the modeling approach—as opposed to its validation—may be allowed to rely on a ground truth, for in topological inference—as opposed to prediction—no ground truth is generally available. We cannot, for example, use a ground truth to tune a hyperparameter without excluding precisely the inferential context we are interested in.

For maximum flexibility and control over the evaluation process, here we use synthetic data drawn from a generative model with a spatially varying distribution of one or two joint binary variables. The spatial variability of the distribution is determined by the locale and extent of shapes with a fractal boundary. Fractals characteristically exhibit detail across an infinite range of spatial scales, which makes them ideal candidates for a spatially structured ground truth with sensitivity to the widest possible range of spatial scales. The use of binary variables to generate two distinct signal levels for the response allows us to focus on data that are generated in a spatially structured way; namely, in a regionally specific fashion under various levels of noise or stochasticity. A full description of the process is provided in supplemental note S2.

### Demonstration with UK Biobank data

To demonstrate the application of GeoSPM to real data, we chose to explore the potential association between a common disease—type 2 diabetes—and a small number of demographic variables in UK Biobank drawn from the area of Greater Birmingham. It should be stressed that the sole purpose of this analysis was to illustrate the application of the method, not to make inferences about the data itself, which would require more detailed investigation than our foundational focus here permits. The objective instead is to illustrate how spatial variation of a variable of interest may be examined, with specific attention to two important contexts: where the effect of the variable must be isolated from a set of known potential confounders, and where the joint effects of two or more variables are of interest. A detailed description of the variable selection and preprocessing is given in supplemental note S2.2.

### Numerical experiments

#### Kriging

We evaluate GeoSPM in comparison with the well-established multivariate geostatistical method of kriging, described in detail in supplemental note S2.3. All kriging computations were done in R using the gstat package,^29^ which is available at https://cran.r-project.org/web/packages/gstat/index.html. For each variable of interest kriging produced an image of the predicted mean and an image of the corresponding prediction variance, which is derived solely from the arrangement of positions in the data, i.e., the prediction variance does not depend on the values of the observations, only on their locations.

#### Synthetic experiments: Noise parameterization

**Figure 1:**
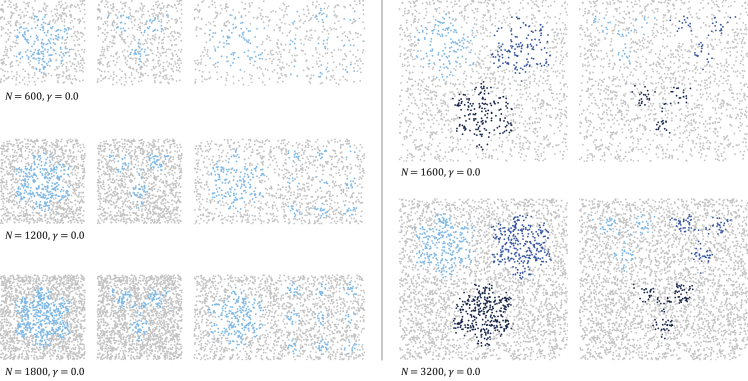
Sampling levels (noise-free, γ=0.0) for the univariate models on the left (N=600,1200,1800), and for the bivariate models on the right (N=1600,3200)

The numerical face validation experiments are based on three univariate models (snowflake, anti-snowflake, snowflake field) and two bivariate models (snowflake, anti-snowflake) as depicted in Figures S2 and S3. For all models, we ran experiments at different sampling levels, $N_{univariate}\;\epsilon \;\left\{600,\;1200,\;1800\right\}$ and $N_{bivariate}\;\epsilon \;\left\{1600,\;3200\right\}$, and increased the noise parameter γ from 0.0 to 0.35 in 0.01 increments (Figure 1). For each triplet (model, N, γ), 10 independent datasets were randomly generated.

Each generated dataset was processed by GeoSPM as well as gstat. For GeoSPM, the spread of the spatial response at locations $x_{i}$, i.e., the spatial distribution of the response following smoothing, was modeled at increasing smoothing parameter values (ℓ=10) using the 95% iso-density diameters of the bivariate normal distribution, $s={\left(10,15,20,25,30,35,40,45,50,55,60\right)}^{\text{T}}$. This measure of spread is the diameter of a circle that contains 95% of the probability mass of a two-dimensional Gaussian distribution at its center. The largest value of the smoothing parameter, 60, was chosen to be half the height of the grid for the univariate models. The regression coefficients estimated by GeoSPM were tested using a one-tailed t test at p < 0.05 FWE (voxel-level, family-wise correction), producing a stack of ℓ binary maps of significant areas for every variable of interest. To derive corresponding maps—one per variable—for kriging, we compared a standardized form of the kriging prediction ${\hat{y}}_{std}\left(j,k\right)$ with the critical value of the upper tail probability p < 0.05 of the normal distribution. We standardized $\hat{y}\left(j,k\right)$ at each grid cell (j,k) using its estimated (positional) variance $\hat{\sigma }\left(j,k\right)$ and assuming a null mean of 0.5 to produce ${\hat{y}}_{std}\left(j,k\right)$:

$${\hat{y}}_{std}\left(j,k\right)=\frac{\hat{y}\left(j,k\right)–{\text{μ}}_{null}}{\hat{\sigma }\left(j,k\right)},where\left(j,k\right)\in D^{\prime },\mu_{null}=0.5.$$

**Figure 2:**
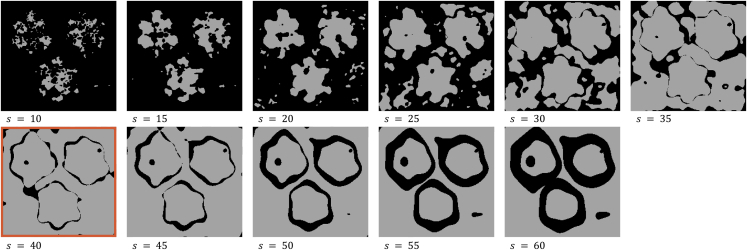
Example of a coverage computation for an instance of the bivariate snowflake model with noise γ=0.1 and N=1600 For each value s of the smoothing parameter, the combined significant areas for all four spatial conditions $\left(Z_{1},\;Z_{2}\right)\in \left\{\left(0,\;0\right),\;\left(1,\;0\right),\;\left(0,\;1\right),\;\left(1,\;1\right)\right\}$ as determined by a separate run of GeoSPM are shaded in light gray. The maximum number of significant grid cells is obtained for s=40, highlighted in red.

For a fair comparison with kriging, one of the ℓ smoothing values and its associated maps produced in a run of GeoSPM had to be chosen. We based this choice on maximizing the spatial coverage by the significant areas at each spatial scale (see Figure 2), while minimizing the spatial overlap between them. A spatial condition in the context of the observed variables $Y\in R^{P}$ in our models is obtained by applying a threshold of 0.5 to all observations, recording as 1 if an observed variable value exceeds the threshold or 0 if it does not. Each observation of a univariate model can thus be assigned one of two spatial conditions, or one of four conditions in the case of a bivariate model. We obtain the significant areas for each spatial condition by running a separate analysis in GeoSPM on a set of data that represents the spatial condition of each observation as a one-hot encoding, i.e., with each category represented as a set of binary dummy variables.

This approach enabled us to derive a score for each of the ℓ smoothing values, which simply comprised the total number of significant grid cells that appeared for exactly one of the spatial conditions, thereby ignoring any overlap. The smoothing value with the highest score was selected, together with the binary maps of significant areas computed from it. Ties were broken by choosing the smallest scale.

The binary maps for each variable were assessed relative to their respective target maps, which were derived by thresholding the corresponding marginal distribution of the model, adding grid cells with a probability greater than 0.5 to the target. We applied a number of representative image segmentation metrics to each pair of maps, computing a mean score over the 10 repetitions of each unique triplet (model, N, γ) and variable. The following metrics were used^30^^,^^31^: Jaccard index, Dice score, Matthew’s correlation coefficient, symmetric uncertainty and the modified Hausdorff distance (as a fraction of the length of the model diagonal). The mean score and deviation for each metric and computation method were aggregated into the plots reported below.

#### Numerical experiments: Interaction parameterization

These experiments used the snowflake interaction model above and comprise observations of its variables, augmented by an interaction term. The interaction term is formed in the usual manner, by multiplying the observed values for both variables, yielding augmented observations: $y^{\prime }\;=\;{\left(y_{1},y_{2},y_{1}\cdot y_{2}\right)}^{\text{T}}$. The regional arrangement of the model is the same as the one employed for the bivariate snowflake model shown on the left of Figure S2. A single sampling level $N_{interaction}=15000$ was used and the interaction parameter $c_{3}$ was increased from 0.25 to 0.5 in steps of 0.05. For each level of $c_{3}$, R=10 independent datasets were randomly generated. We set a single value for the smoothing parameter s=60, which was the highest value for the noise experiments. As before, a one-tailed t test at p < 0.05 FWE (voxel-level family-wise correction) determined areas of significance and the same set of image segmentation metrics was computed for the binary maps.

#### UK Biobank experiments

Results for the UK Biobank data were obtained by a single invocation of GeoSPM for each of the four models listed in Table S6. We choose a smoothing value of 7 km, specified as the diameter of a patch enclosing 95% of the density the bivariate normal distribution with equal variances. This represents 20% of the width and height of our Birmingham analysis area, and seemed appropriate for identifying local variation sensitive to the plausible spatial scale of distinct geographically defined communities. This time, a two-tailed t test at p < 0.05 FWE (voxel-level family-wise correction) was used for thresholding the statistic maps. Analysis is restricted to areas where the combined smoothing density of all observations is at least 10 times the kernel peak value.

#### Ethical approval

UK Biobank has approval from the North West Multi-centre Research Ethics Committee as a Research Tissue Bank (RTB) approval. This approval means that researchers do not require separate ethical clearance and can operate under the RTB approval.

## Results

Our numerical experiments with a known generative model enabled us to measure performance against a known ground truth under circumstances varying in density of sampling and contamination with noise, enclosing the range likely to obtain in real-world scenarios. It also permits robust evaluation of graded interaction effects. In total, 2,160 independent simulations with synthetic data were performed for the univariate models, 1,440 for the bivariate models and 60 for the interaction model. Summarizing scores within the three sets of simulations, we derive performance curves for GeoSPM and kriging solutions in each case. We then proceed to illustrate the application of GeoSPM to real world data from UK Biobank.

### Synthetic models

**Figure 3:**
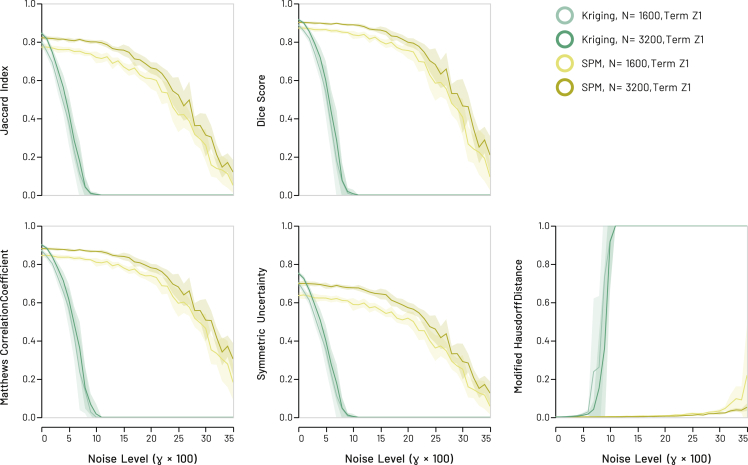
Synthetic snowflake models: recovery scores for GeoSPM and kriging of model term $Z_{1}$ in the low (*N* = 1600) and high (*N* = 3200) sampling regimes Lines denote the mean score across 10 random model realizations, shaded areas its SD to either side of the mean. Areas of overlapping performance are identified by additive shading. GeoSPM degrades more slowly and gracefully as noise increases compared with kriging. Comparable results for model term $Z_{2}$ are shown in Figure S10.

**Figure 4:**
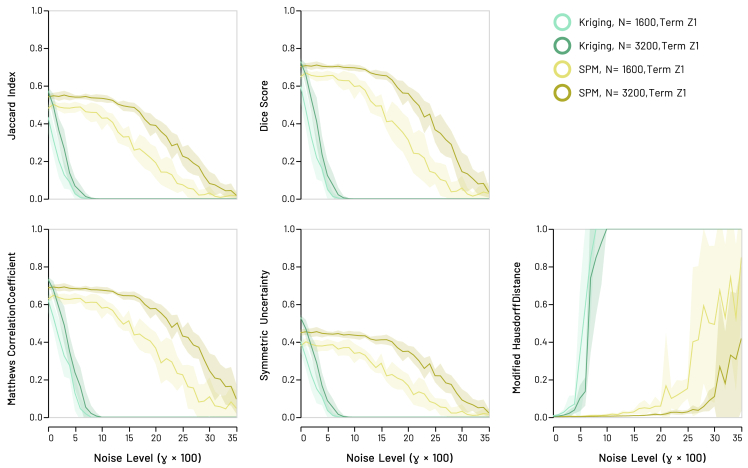
Synthetic anti-snowflake models: recovery scores for GeoSPM and kriging of model term $Z_{1}$ in the low (*N* = 1600) and high (*N* = 3200) sampling regime Lines denote the mean score across 10 random model realizations, shaded areas its SD to either side of the mean. Areas of overlapping performance are identified by additive shading. As is the case with the snowflake models, GeoSPM degrades more slowly and gracefully as noise increases compared with kriging. Comparable results for model term $Z_{2}$ are shown in Figure S11.

**Figure 5:**
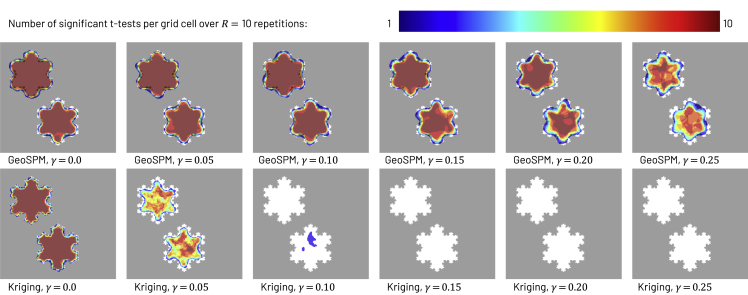
Recoveries of variable $Z_{1}$ in the synthetic bivariate snowflake model across R = 10 repetitions for GeoSPM in the top row and kriging with a Matérn kernel and nugget component in the bottom row, both in the high sampling regime (N = 3200) Grid cells that lie in the target region are shown in white, those outside in gray. The number of significant tests out of 10 repetitions is superimposed in color for each grid cell: dark blue indicates at least one significant test and dark red indicates the maximum number of 10, while cells with no significant test did not receive any color. Kriging only produces recoveries up to a γ value of 0.10, whereas GeoSPM still produces recoveries for much higher values of γ. GeoSPM used t tests with a family-wise error corrected p value of 0.05, for kriging we applied a z-test with an uncorrected p value of 0.05, a null mean of 0.5 and a sample deviation obtained from the (positional) kriging variance estimate, as described in the section on “synthetic experiments: noise parameterization”. Additional kriging recoveries are shown in Figures S21–S25 of supplemental note S3.5.

Displayed in the following figures are sets of independent simulations comparing the performance of GeoSPM (in yellow) versus kriging (in green) as a function of contaminating noise, measured by five different indices of retrieval fidelity, using the snowflake (Figure 3) or anti-snowflake (Figure 4) bivariate ground truths, and low or high data sampling regimes (similar results for the univariate ground truths are reported in Figures S7–S9 in supplemental note S3.1, as are the results for the second term in the bivariate models in Figures S10 and S11 of supplemental note S3.2). A visual summary of the recovered binary maps underlying these performance curves—for the bivariate snowflake model and the high sampling regime—affords a further qualitative comparison between the two methods (Figure 5).

It is evident that GeoSPM offers superior efficiency across most of the noise range in all models and on all metrics. GeoSPM models generally remain stable at higher levels of noise than kriging. Both GeoSPM and kriging exhibit sensitivity to the sampling regime, both in terms of variability and stability, but the effects are dwarfed by the difference between the two approaches. The type of ground truth has negligible impact. In addition, neither changing the (cross-) covariance function used for kriging from a Matérn function to a Gaussian nor applying a different regime for dealing with coincident observations—such as averaging—yields a discernible improvement to the performance of kriging in this context (see Figure S12 in supplemental note S3.3).

Additional results based on an extended selection of covariance models for kriging show comparable outcomes and are similar to those presented in Figures 3, 4, and 5, as documented in Figures S13–S20 in supplemental note S3.4 and Figures S21–S25 in supplemental note S3.5. For a more in-depth view of the behavior of kriging parameters and variograms, refer to supplemental note S3.6 and S3.7.

**Figure 6:**
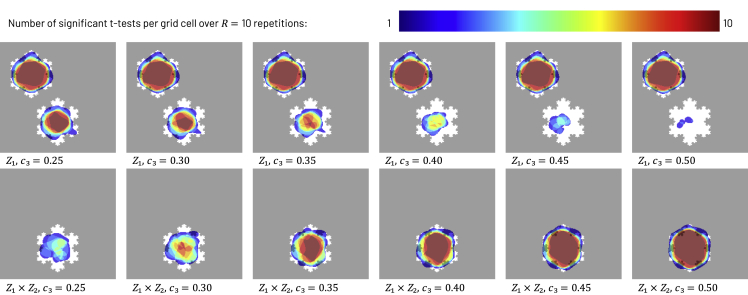
Recoveries produced by GeoSPM for the synthetic interaction model across R = 10 repetitions for variable $Z_{1}$ in the top row and term $Z_{1}\times \;Z_{2}$ in the bottom row, with *N* = 15,000 samples Grid cells that lie in the target region are shown in white, those outside in gray. The number of significant tests out of 10 repetitions is superimposed in color for each grid cell: dark blue indicates at least one significant test and dark red indicates the maximum number of 10, while cells with no significant test did not receive any color. Starting with a low value for the interaction effect $c_{3}$ on the left, recovery of the interaction term $Z_{1}\times \;Z_{2}$ in region $R_{3}$ is weak, while recovery for variable $Z_{1}$ in the same region is stronger. This correlates with the fact that observations (1,1) occur with only a slightly elevated probability $p_{3}=0.6$ compared with their null probability of 0.525 when $c_{3}$ equals 0 in the same setting. As $c_{3}$ increases toward the right, recovery in the same region for term $Z_{1}\times \;Z_{2}$ increases ($p_{3}=0.725$ at the right), while recovery for variable $Z_{1}$ decreases (probability $p_{1}=0.125\;$ at the right for observing (1,0), which is half of what it would be if there was no interaction effect). GeoSPM used t tests with a family-wise error corrected p value of 0.05.

**Figure 7:**
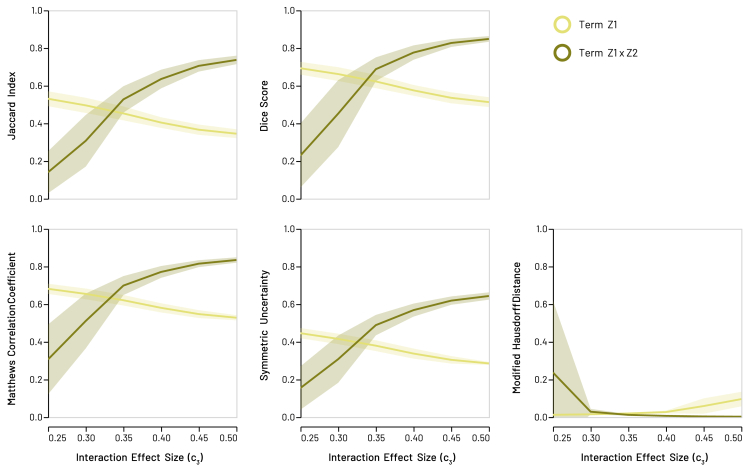
Synthetic snowflake interaction model: recovery scores for SPM model variable $Z_{1}$ and term $Z_{1}\times \;Z_{2}$ with *N* = 15,000 samples Lines denote the mean score across 10 random model realizations, shaded areas its SD to either side of the mean. We increase the approximate interaction effect in region $R_{3}$ of the grid from left to right, so that the probability of observing (1,1) grows while the probability of observing (1,0) or (0,1) shrinks (the probability of observing (0,0) stays the same). As a result, scores increase for the interaction term $Z_{1}\times \;Z_{2}$ as it captures more of the overall variance, whereas scores for variable $Z_{1}$ decrease, until the only significant recovery occurs in region $R_{1}$, which represents half of the target for $Z_{1}$ and explains why the overall decrease saturates.

The recoveries obtained from simulations of the interaction model clearly show GeoSPM’s ability to detect an interaction between two spatially distributed factors, even toward the lower end of the approximate interaction effect size range (Figure 6). Plots of the same five indices above demonstrate successful retrieval for these interaction simulations quantitatively (Figure 7). As we increase the size of the approximate interaction effect $c_{3}$, retrieval results for the interaction term $Z_{1}\times \;Z_{2}$ approach those of the previous, noise-free bivariate snowflake model (setting aside the different sampling regimes). At the same time, recovery for variable $Z_{1}$ decreases in the interaction region $R_{3}$ (but not elsewhere), as the interaction term explains more variance. Once the recovery for variable $Z_{1}$ in region $R_{3}$ has vanished, the corresponding retrieval scores are about half of those for the same term in the noise-free model, which agrees with our expectation, because only one of two snowflake shapes in the target are still retrieved at that stage.

### UK Biobank models

In real-world scenarios there is usually no explicit ground truth against which an inference can be tested: the conclusion rests on the integrity of the underlying statistical assumptions. Our illustrative analysis of UK Biobank data^24^ therefore does not seek to quantify GeoSPM’s fidelity but to demonstrate its potential utility in the medical realm. We focus on two aspects: the derivation of marginalized spatial maps that disentangle a factor of interest from a set of (interacting) confounders, and the use of conjunction analysis to identify regions jointly modulated by multiple spatially organized factors.

The propensity to develop type 2 diabetes is related to age, sex, BMI, and household income, among other factors: a known pattern clearly replicated in UK Biobank. A map of diabetes may therefore reflect not just the propensity to develop the disease but also the spatial structure of associated factors, both causal and incidental. If we are pursuing a previously unknown spatial factor—pollution, for example,^32^^,^^33^^,^^34^—we would wish to void our diabetes map of known confounders, yielding a spatial distribution of fully marginalized propensity.

**Figure 8:**
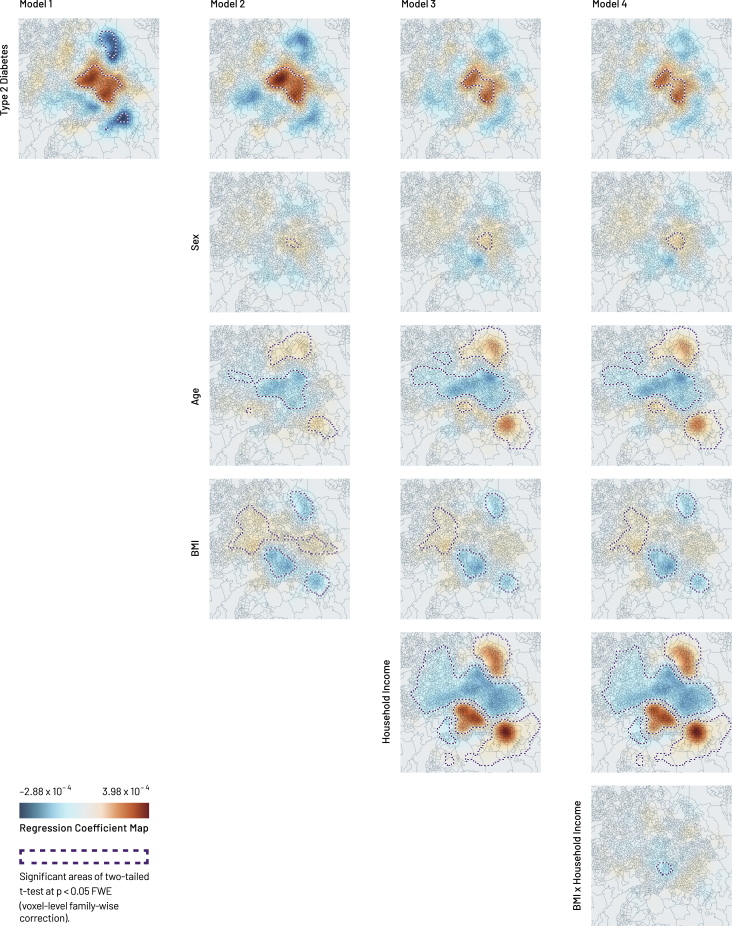
GeoSPM results for the four UK Biobank models of Birmingham (one column per model) Geographic regression coefficient maps are shown with outlines of significant areas in the corresponding two-tailed t test at p < 0.05 FWE (voxel-level family-wise correction). The smoothing parameter value is 7,000 m.

**Figure 9:**
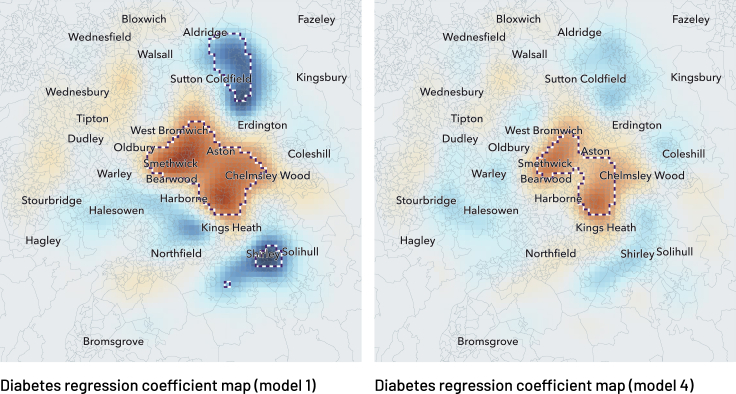
Geographic regression coefficient maps with location names for a single run of UK Biobank models 1 and 4 Model 1 is a univariate model of diabetes, model 4 adds sex, age, BMI, household income, and an interaction term BMI × household income. Outlines show significant areas in the corresponding two-tailed t test at p < 0.05 FWE (voxel-level family-wise correction). The smoothing parameter value is 7,000 m. The color map scale is the same as in Figure 8.

We demonstrate GeoSPM on individual-level UK Biobank data drawn from Birmingham. Figure 8 presents the regression coefficient maps and significant t test areas for four separate models of diabetes with incrementally greater numbers of covariates. The first, univariate, model of diabetes (model 1) reveals an extensive concentric organization, positive in the center and negative in the periphery, especially in the north and south. The map becomes more tightly circumscribed with the addition of sex, age, and BMI in model 2: the two negative areas in the north and south are no longer significant, and a stronger negative region emerges west of the center. With the addition of further covariates and their interactions, the spatial structure of diabetes that remains unexplained converges on a set of focal, central regions, displayed in detail in comparison with the univariate model in Figure 9. Here the regional expression of diabetes is not explained by the modeled covariates, suggesting the presence of other factors in play to be subsequently investigated. In general, the ensemble of significant areas for each model indicates the spatial structure that remains unexplained for the corresponding set of covariates, while the intensity and sign of each regression coefficient map represent the degree of spatial association of its covariate in the ensemble. With this in mind, the individual maps for diabetes represent a spatial distribution of propensity marginalized against the other covariates, but not an absolute rate of disease.

**Figure 10:**
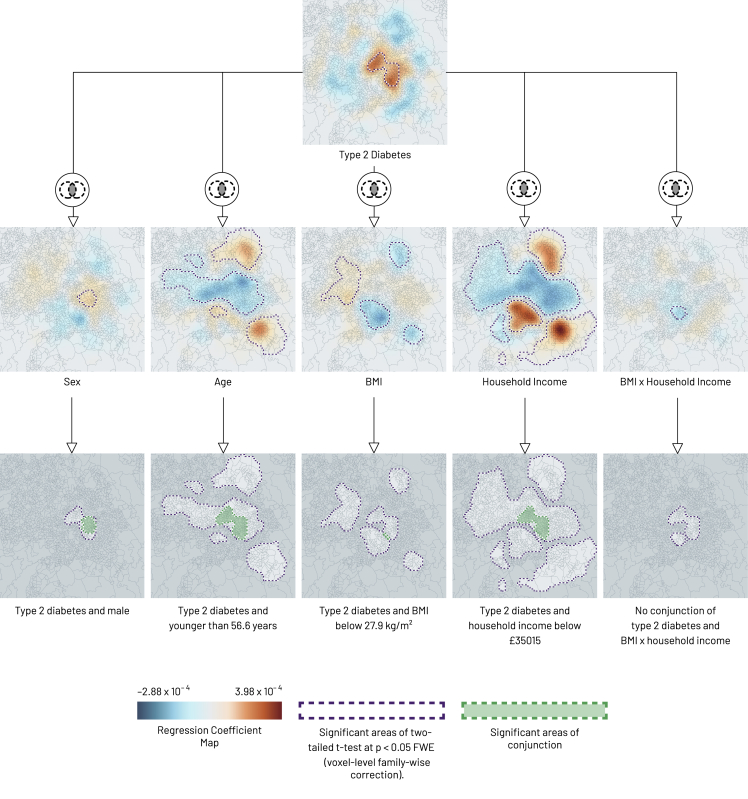
Binary conjunctions of geographic regression significance maps for a single run of UK Biobank model 4 A binary conjunction is formed of the significant areas of a two-tailed t test at p < 0.05 FWE (voxel-level family-wise correction) between type 2 diabetes and, in turn, sex, age, BMI, household income, and BMI × household income. Purple outlines show significant areas in the two-tailed t test of each variable, green outlines show significant areas of conjunction: significant areas of conjunction arise in diabetes combined with each of sex (male), age (younger than 56.6 years), BMI (below 27.9 kg/m^2^), and household income (below £35,015). No significant areas of conjunction exist for diabetes and BMI × household income. Locations shown in darker gray tone are not significant for any of the variables. The smoothing parameter value is 7,000 m.

**Figure 11:**
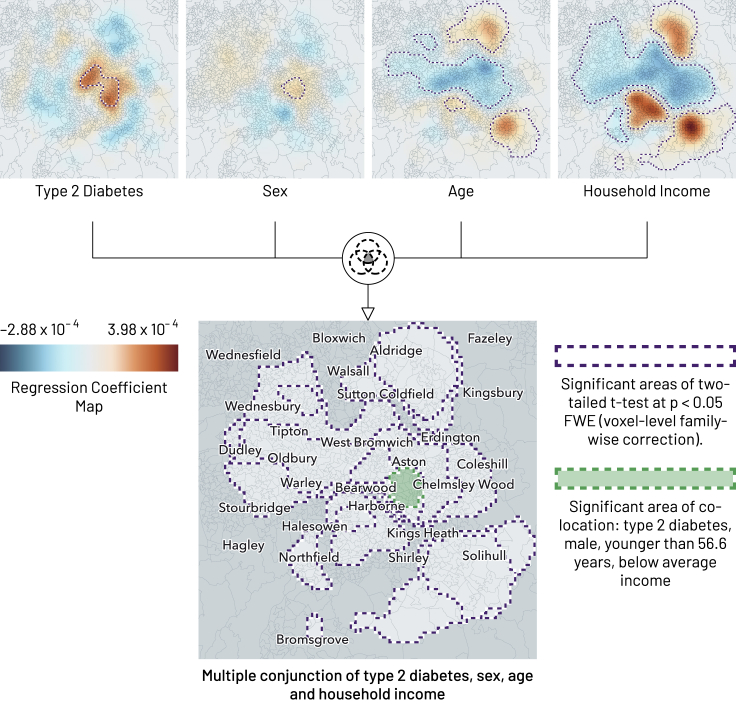
Example of a multiple conjunction (here quaternary) of geographic regression significance maps for a single run of UK Biobank model 4 A binary conjunction is formed of the significant areas of a two-tailed t test at p < 0.05 FWE (voxel-level family-wise correction) between type 2 diabetes and, in turn, sex, age, BMI, household income, and BMI × household income. Purple outlines show significant areas in the two-tailed t test of each variable, green outlines show significant areas of conjunction: we can identify a significant area where younger males of lower income are associated with having type 2 diabetes in Birmingham. The smoothing parameter value is 7,000 m.

We can now also examine the *conjunctions* of multiple maps, not necessarily derived from the same model, within a second-level analysis. Conjunctions are here simply the intersections of two or more thresholded t maps, identifying areas where the regression coefficients and their associated variables are jointly significant. Applied to the outputs of our most complex model above, the approach and resulting conjunctions are shown in Figure 10. Pairwise conjunctions show a single region where diabetes and male sex are colocalized; a distinct region where diabetes and age are inversely associated; a very narrow region with an inverse relation between diabetes and BMI; and a single region where diabetes is inversely related to household income. Finally, a three-way conjunction identifies a region where diabetes is spatially associated with younger age, male sex, and lower income (Figure 11). Such conjunction maps identify regions where two or more variables of interest are significantly expressed together, representing subpopulations whose intersectionally characteristic features may inform responsive action or further investigation.

This concludes our illustration of GeoSPM. Note that the fact that GeoSPM was able to identify significant regionally specific effects provides a provisional form of predictive validity; under the assumption that these effects were present in the population—and could therefore be used to predict response variables.

## Discussion

We propose, implement, and validate an approach to drawing spatial inferences from sparse clinical data, extending to geostatistics a mature, principled framework for topological inference—SPM—that is well established in the realm of brain imaging. Compared with kriging, GeoSPM combines similar fidelity under optimal conditions with substantially less sensitivity to noise and under-sampling, greater robustness to failure, faster computation, graceful handling of multiple scales of spatial variation, and formal inferential support. Its simplicity and accessibility facilitate widespread application of the comprehensive software implementation we have provided, built on the validated SPM open-source codebase, across a wide range of applications in medicine and beyond. Here, we consider six points concerning the application, extension, and limitations of our approach.

First, GeoSPM is applicable to problems of topological spatial inference, whose formulation conforms to the minimal assumptions of the underlying statistical framework. The types of data, the choice of model evaluated at each point, and the size and density of the evaluated grid are not under any strong constraint. Eliminating the spatial dimension allows each point-wise model to be more flexible than the data or computational resource could otherwise sustain. The model could even be complicated spatially, extending to encompass a local patch within otherwise the same framework. This is a key strength in medical applications, where a spatial effect typically needs to be disentangled from a wide array of others.

Second, although here prototyped on temporally stationary data, GeoSPM can be configured with time instead of the spatial scale in the third dimension, enabling graceful modeling of both spatial and temporal correlations. This has been used, for example, in the context of electrophysiology^35^ where extra dimensions can include peristimulus time or, indeed, fast oscillatory frequencies. The effects of manipulating noise and spatial dependencies can then be evaluated across individual time series. Equally, the third dimension could be used for multimodal data projected within the same grid, informing the inference by multiple sampling modalities.

Third, the smoothing parameter may be constrained by prior knowledge or independent estimation from the data, even if evaluating a set of models over a plausible range is arguably the most robust approach. One may alternatively rely on the properties of the inferred maps, as suggested in our validation analyses. All competing spatial modeling frameworks rely on chosen parameters to some degree; ours is reduced to a single readily interpretable one.

Fourth, no model could perfectly remedy defects in the data itself, such as inadequate or biased coverage. The former can be mitigated by confining inference to spatial locations exhibiting sufficient sampling density; the latter, analogously to structured missingness, is not easily remediable within this or any other inferential framework, and presents no more or less of a problem.

Fifth, GeoSPM, like SPM itself, is a platform for standard frequentist statistical inference, revealing the organization of spatially structured variables without causal implications of any kind. But, also like SPM, it is open both to Bayesian extensions, and causal modeling upstream or downstream of the core framework. There are many ways of querying data, both with classical mass univariate and Bayesian analyses of this kind. Although not illustrated here, model comparison using the *F*-statistic is a common application that could be enabled by GeoSPM. For example, one could ask whether household income has an effect on the regional prevalence of diabetes, having accounted for other demographic variables, by comparing (general linear) models that do and do not include household income as an explanatory variable.

Finally, the SPM approach, in any formulation, is designed for topological inference, not discrimination between distributed spatial patterns, which may also arise in healthcare, and requires explicit modeling of spatial interactions that only a multivariate model could conceivably deliver. Indeed, such use would violate the underlying assumption of benign regional dependence, as do analogous attempts in the domain of lesion-deficit mapping of the brain.^36^ GeoSPM maps may nonetheless be used to select features where the fragility of the multivariate model, or the applicable data regime, compel it.

## Data Availability

The data analyzed in this study are available on application to UK Biobank (https://www.ukbiobank.ac.uk). The open-source software implementation of GeoSPM presented in this study is available on GitHub: https://github.com/high-dimensional/geospm (https://doi.org/10.5281/zenodo.7258971).

## Supplemental information

Document S1. Supplemental experimental procedures, Figures S1–S56, Tables S1–S7, and supplemental notes

Document S2. Article plus supplemental information
