# Supplementary material for: GeoSPM: Geostatistical parametric mapping for medicine
Source: Patterns (N Y). 2022 Dec 9;3(12):100656. doi: 10.1016/j.patter.2022.100656 (PMC9768692; doi:10.1016/j.patter.2022.100656)
Supplement: Document S1. Supplemental experimental procedures, Figures S1–S56, Tables S1–S7, and supplemental notes [file mmc1.pdf]

Patterns, Volume *3*

Supplemental information

GeoSPM: Geostatistical parametric mapping for medicine

Holger Engleitner, Ashwani Jha, Marta Suarez Pinilla, Amy Nelson, Daniel Herron, Geraint Rees, Karl Friston, Martin Rossor, and Parashkev Nachev

Supplemental Note

S1 Bibliographic analysis

Geospatial field bibliometrics were computed through a search of the titles and abstracts of the entire medical corpus from Microsoft Academic Graph from January 1990-March 2019 cited at least once (17.1 million papers), filtered by keyword string matching (non case-sensitive) within abstracts on the following terms: "geo*" & "map*" & "illness*|disease*|health*". This returned 1897 papers, with mean citations (normalised by the average citation count of a paper in that journal) 1.67 (sd 2.87).

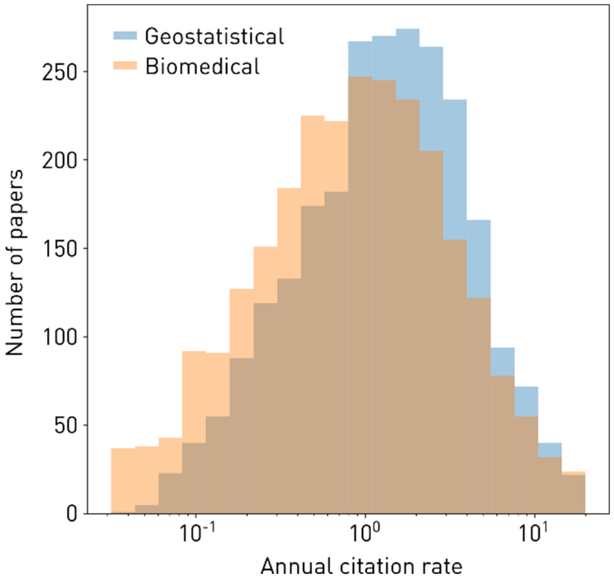

Figure S1. Overlapped histograms of the decimal log-transformed annual citation rates of 1897 identified journal papers at the intersection of spatial analysis and medicine cited more than once (blue), and an identically filtered random sample of non-spatial biomedical papers (orange), published between 1990 and 2019. The untransformed distributions are significantly different on a Mann-Whitney U test, p<0.001.

S2 Supplemental methods

S2.1.1 Synthetic data and generative models

We start by defining a spatial domain as a rectangular subset $D \coloneqq [0,a) \times [0,b) \subset \mathbb{R}^2$ for some $a, b \in \mathbb{N}^+$, and restrict a and b to positive integers so that

$$D = [0,a) \times [0,b) = \bigcup_{j=1}^{a} \bigcup_{k=1}^{b} [j-1, j) \times [k-1, k)$$

has a natural decomposition into $a \times b$ grid cells with coordinates $(j,k) \in D' \coloneqq \{1, \dots, a\} \times \{1, \dots, b\}$. Assuming that there are P binary factors in the generative model, the simulated response variables can be written as the components of a random vector $\boldsymbol{Z} = (Z_1, \dots, Z_P)^{\mathrm{T}} \in \{0,1\}^P$, which is sampled at

random grid cells $\boldsymbol{W} \in D'$ in the underlying space. Our data generation mechanism is based on the factorisation of the joint distribution of $\boldsymbol{Z}$ and $\boldsymbol{W}$ as

$$\Pr(\boldsymbol{Z}, \boldsymbol{W}; \boldsymbol{\theta}) = \Pr(\boldsymbol{Z} \mid \boldsymbol{W};\ \boldsymbol{\theta})\Pr(\boldsymbol{W})$$

so that the response variables $(Z_1, \dots, Z_P)^{\mathrm{T}}$ are conditioned on location $\boldsymbol{W}$ with model parameters $\boldsymbol{\theta}$ fixed at $(\theta_1, \theta_2,\ \dots)^{\mathrm{T}}$. $\boldsymbol{W}$ is distributed independently of $\boldsymbol{Z}$ and uniformly over D'.

The conditioning allows breaking down the distribution of $\boldsymbol{Z}$ spatially, by partitioning the grid D' into a small number of (not necessarily) continuous regions $R_k \subseteq D' : k = 0, \dots, K-1$ for which local distributions $\mathrm{P}_k(\boldsymbol{Z}; \boldsymbol{\theta}) \coloneqq \Pr(\boldsymbol{Z} \mid \boldsymbol{W} \in R_k;\ \boldsymbol{\theta})$ can be specified for each region, k. As $P \in \{1,2\}$ for the models considered here, at most four probabilities are required for each of these local distributions. The partitions are based on arrangements of fractal shapes, shown in Figure S2 for the two bivariate models and in Figure S3 for the univariate models.

In these images, a single pixel represents a grid cell, and the shading indicates the distinct distributions $\mathrm{P}_k(\boldsymbol{Z}; \boldsymbol{\theta})$. The resolution of the bivariate models is 220 by 210 grid cells, whereas the resolution of the univariate models is 120 by 120 grid cells. Geometry for the fractal shapes is constructed by recursively substituting the edges of a (start) shape with a simple curve (Figure S4) and then rasterising the resulting polygon into the grid using MATLAB's poly2mask function. We wish to examine the effect of noise and interactions in our numerical experiments, which leads us to consider two distinct parameterisations of the distributions P_k.

S2.1 Parameterisation of $\mathbf{P}_k(\boldsymbol{Z}; \boldsymbol{\theta})$ for Examining Noise

The first parameterisation is expressed in terms of a function $\mathrm{p}_{noise}(\cdot)$ with parameters p and q:

$$\mathrm{P}_k(\boldsymbol{Z}; \boldsymbol{\theta}) = \mathrm{p}_{noise}(Z_1, Z_2;\ p, q), \qquad p = \theta_k, q = \theta_{k+K}$$

$\mathrm{p}_{noise}(\cdot)$ is summarised in Table S1. The parameters p and q are simply the values of the marginal probabilities $\mathrm{p}_{noise}(z_1 = 1)$ and $\mathrm{p}_{noise}(z_2 = 1)$ and are sufficient for defining $\mathrm{p}_{noise}(Z_1, Z_2;\ p, q)$ if Z_1 and Z_2 are assumed to be independent.

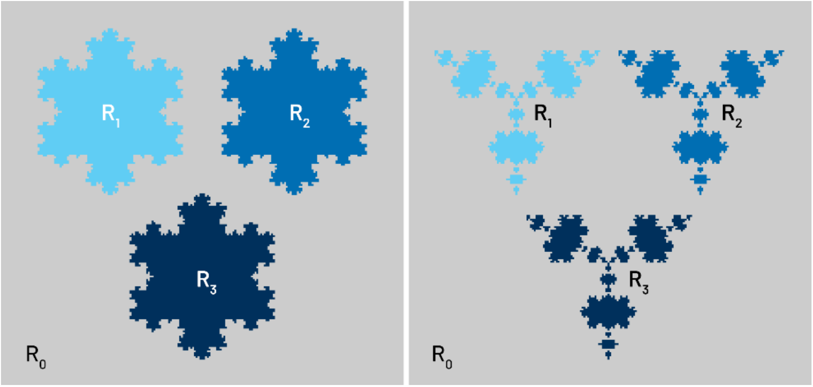

Figure S2. The four distinct regions $R_k : k = 0, \dots, 3$, of the joint conditional probability $\Pr(\boldsymbol{Z} \mid \boldsymbol{W};\ \boldsymbol{\theta})$ for the snowflake model (left) and the anti-snowflake model (right).

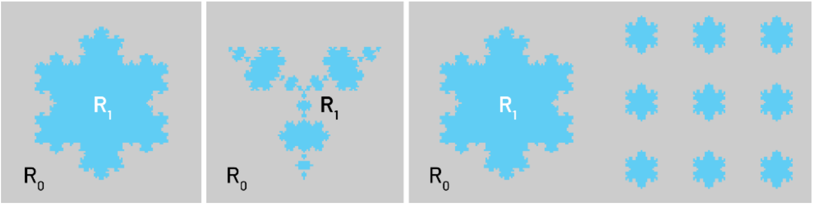

Figure S3. The two distinct regions $R_k : k = 0, 1$ of the conditional probability $\Pr(Z \mid \boldsymbol{W};\ \theta)$ for the univariate models: snowflake model (left), anti-snowflake model (middle) and snowflake field model (right).

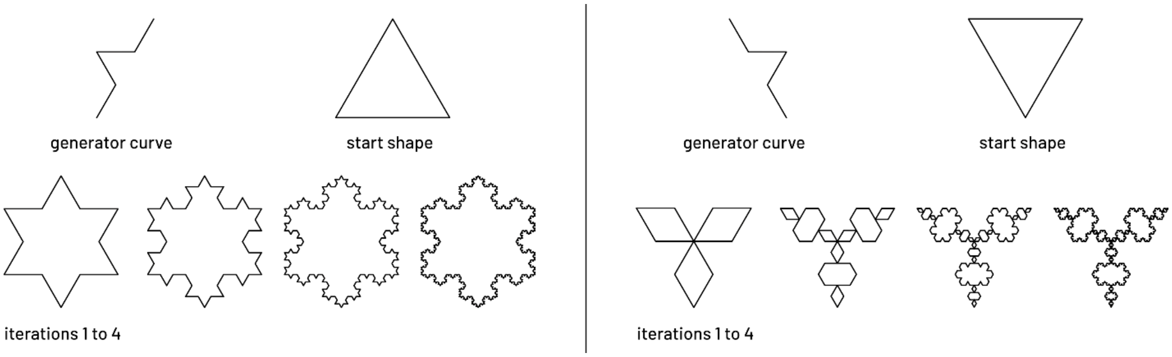

Figure S4. The geometric construction used for the fractal shapes. Koch snowflake on the left, Koch anti-snowflake on the right.

$\mathrm{p}_{noise}(Z_1, Z_2;\ p, q)$	$z_1 = 0$	$z_1 = 1$	$\mathrm{p}_{noise}(z_2)$
$z_2 = 0$	$(1-q)(1-p)$	$(1-q)p$	$1 - q$
$z_2 = 1$	$q(1-p)$	qp	q
$\mathrm{p}_{noise}(z_1)$	$1 - p$	p	1

Table S1. Probability table for the bivariate local distribution function $\mathrm{p}_{noise}(Z_1, Z_2;\ p, q)$

As we move in D from one region to the next, we simulate spatially distinct conditions of the variables $Z_1$and Z_2 by changing the regional expectations $\mathbb{E}_k[(Z_1, Z_2)]$ through $\mathrm{p}_{noise}(Z_1, Z_2;\ p, q)$: For the four regions of the bivariate models in Figure S2, the corresponding expectations are listed in Table S2, together with the respective values for p and q. In the absence of uncertainty, the models generate the expected values in each region exactly, thus R_0 *only*generates observations $(0,\ 0)$, R_1 *only* $(1,\ 0)$, and so on.

As more uncertainty (i.e., noise) is introduced—by adjusting the values for p and q—the overall pattern of observations still holds, but other values have a non-zero probability of occurrence: R_0 *mostly* generates observations $(0,\ 0)$, R_1 *mostly* $(1,\ 0)$, and so on, until a maximum level of uncertainty is reached and each observation is equally probable in every region. By expressing p and q in terms of a single parameter $\gamma \in [0, \ldots, 0.5]$ (the last two columns in Table S2), we can easily vary the degree of observation noise from a spatially deterministic and regionally differentiated form, to one where all regional differentiation is lost (see Figure S5 for an example).

	$\mathbb{E}_k[(Z_1, Z_2)] \rightarrow \ldots$	$p \rightarrow \ldots$	$q \rightarrow \ldots$	$p(\gamma)$	$q(\gamma)$
R_0	(0,0)	0	0	γ	γ
R_1	(1,0)	1	0	$1-\gamma$	γ
R_2	(0,1)	0	1	γ	$1-\gamma$
R_3	(1,1)	1	1	$1-\gamma$	$1-\gamma$
$max.uncertainty$	(0.5,0.5)	0.5	0.5		

Table S2. Expected values of $Z_1$and Z_2 in each region of the Snowflake and Anti-Snowflake models shown in Figure 1 for the given values of p and q.

The parameter vector $\boldsymbol{\theta}$ in $\mathrm{P}_k(\boldsymbol{Z}; \boldsymbol{\theta})$ for the bivariate models is determined by γ as shown in Table S2 and has the following structure:

$$\boldsymbol{\theta} = \boldsymbol{\theta}_\gamma, \qquad \boldsymbol{\theta}_\gamma \coloneqq [\gamma, 1-\gamma, \gamma, 1-\gamma, \gamma, \gamma, 1-\gamma, 1-\gamma]$$

We consider the deviation of observed values of $\boldsymbol{Z}$ when γ is non-zero from the expected values when γ is 0 as simulating noise induced by confounding variables that are not captured in the data. Its effect of degrading the observable spatial differentiation of the variables of interest is key in our analysis of the performance of GeoSPM, and so we treat γ as an independent variable in these numerical experiments.

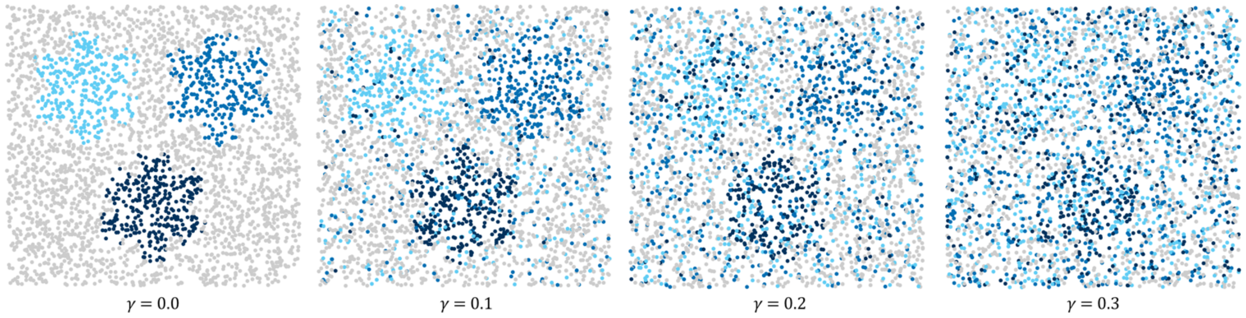

Figure S5. Random realisations of the bivariate Snowflake model for different levels of γ (from left to right).

Of course, real data also exhibit additive measurement noise. To simulate measurement noise an observation $\boldsymbol{Y} \in \mathbb{R}^P$ with location $\boldsymbol{X} \in D$ is derived from $\boldsymbol{Z}$ and $\boldsymbol{W}$ by adding random effects sampled from multidimensional uniform distributions:

$$\boldsymbol{Y} = \boldsymbol{Z} + \boldsymbol{\zeta}, \qquad \boldsymbol{\zeta} \sim \text{uniformly on } I_1 \times \ldots \times I_P, \qquad I_i = [0, 0.005]$$
$$\boldsymbol{X} = \boldsymbol{W} + \boldsymbol{\omega}, \qquad \boldsymbol{\omega} \sim \text{uniformly on } [0,1) \times [0,1)$$

A practical benefit of applying 'spatial noise' $\boldsymbol{\omega}$ to $\boldsymbol{W}$ is that the probability of two randomly drawn elements $(\boldsymbol{y}_i, \boldsymbol{x}_i)$ and $(\boldsymbol{y}_j, \boldsymbol{x}_j)$ coinciding at the same location $\boldsymbol{x}_i = \boldsymbol{x}_j$ is minimised. This is relevant for the geostatistical method used for validation in these numerical experiments, because such collisions would produce singular, non-positive definite covariance matrices, when predicting observations and need to be removed from any data set prior to model estimation.

As GeoSPM only operates in terms of the discrete space D', it always applies the congruency $\boldsymbol{X} \equiv \boldsymbol{W}$ and so the added noise $\boldsymbol{\omega}$ has no effect. We will also consider an alternative method for resolving spatially coincident observations—by averaging observations from the same location—and provide corresponding results for kriging below.

We can now summarise the procedure for generating a spatially-referenced data set of size N and noise level γ_0 as follows:

1. Draw a uniform sample of N grid cell coordinates $\boldsymbol{w}_i \in \{1, \ldots, a\} \times \{1, \ldots, b\},\ i: 1, \ldots, N$
2. For each grid cell coordinate $\boldsymbol{w}_i$ draw a sample $\boldsymbol{z}_i$ from $\mathrm{P}_k(\boldsymbol{Z}; \boldsymbol{\theta}_\gamma)$, where $\boldsymbol{w}_i \in R_k$
3. Obtain an observation $\boldsymbol{y}_i$ at location $\boldsymbol{x}_i$ by adding small amounts of random noise to $\boldsymbol{z}_i$ and $\boldsymbol{w}_i$:

$$\boldsymbol{y}_i = \boldsymbol{z}_i + \boldsymbol{\zeta}_i$$
$$\boldsymbol{x}_i = \boldsymbol{w}_i + \boldsymbol{\omega}_i$$

S2.1.2 Parameterisation of $\mathbf{P}_k(\boldsymbol{Z}; \boldsymbol{\theta})$ for Examining Interactions

A common feature of regression modelling is the inclusion of interaction terms, when there is reasonable belief that the marginal effect of one variable depends on the value of another. In a spatial setting, an interaction could be described as the degree to which the observation of a value of one variable is affected by the value of another variable at the same location. Therefore, GeoSPM's ability to detect interactions merits additional evaluation.

Here, a second parameterisation of the local distributions P_k can be motivated by interpreting the spatial response introduced earlier as a *concentration* instead of a measure of closeness. The constituent probabilities of the P_k are then the *regionally expected concentrations* of their respective observations $(Z_1, Z_2) \in \{(0,0), (1,0), (0,1), (1,1)\}$. As the response of local univariate regression models, these concentrations should have approximately *additive structure*, given the objective is to model interactions. To this end, we define the P_k as a function $\mathrm{p}_{interaction}(\cdot)$ with parameters p_0, c_1, c_2 and c_3, which we assign from a global parameter vector $\boldsymbol{\theta}$ for each region R_k:

$$\mathrm{P}_k(\boldsymbol{Z}; \boldsymbol{\theta}) = \mathrm{p}_{interaction}(Z_1, Z_2;\ p_0, c_1, c_2, c_3), \qquad p_0 = \theta_k, c_i = \theta_{k+iK}$$

Table S3 provides a definition of $\mathrm{p}_{interaction}(\cdot)$. The parameters c_i approximate the *effect sizes* induced by the observations of the variables (Z_1, Z_2) in the local univariate regression models, which—due to their binary values—correspond to the effect of Z_1, Z_2 and their interaction $Z_1 \times Z_2$ on the concentration or response. This is only an approximation of effect size, because of the non-linear fall-off of the Gaussian kernels used to synthesize the response.

$\mathrm{p}_{interaction}(Z_1, Z_2;\ p_0, c_1, c_2, c_3)$	$z_1 = 0$	$z_1 = 1$
$z_2 = 0$	p_0	$p_0 + c_1$
$z_2 = 1$	$p_0 + c_2$	$p_0 + c_1 + c_2 + c_3$

Table S3. Probability table for the bivariate local distribution function $\mathrm{p}_{interaction}(Z_1, Z_2;\ p_0, c_1, c_2, c_3)$

The parameters c_i define the respective probabilities relative to the parameter p_0, the probability of generating the observation $(0, 0)$. The c_i have a maximum range of $[-1, \dots, 1]$, subject to the conditions that each p_i is a valid probability ($p_i \in [0, \dots, 1]$) and that all the resulting probabilities add up to 1:

$$p_1 = p_0 + c_1$$

$$p_2 = p_0 + c_2$$

$$p_3 = p_0 + c_1 + c_2 + c_3$$

$$1 = 4p_0 + 2c_1 + 2c_2 + c_3$$

For the interaction experiments, we define the same four distinct regions $R_{0\dots3}$ as for the bivariate snowflake model (shown on the left of Figure 1) in the noise parameterisation and vary the magnitude of the interaction effect c_3 in region R_3. In detail, in region R_0, all observations are equiprobable, representing the null state, as all three effects c_1, c_2 and c_3 are 0. R_1 and R_2 are regions where we either observe a non-zero effect c_1 for variable Z_1 or a non-zero effect c_2 for variable Z_2 but no effect that corresponds to their interaction.

Finally, we construct region R_3 to model an interaction effect c_3 at different intensities while keeping p_0 constant at a non-zero level and assuming $c_1 = c_2$. Based on (arbitrarily) setting p_0 to a small non-zero value of 0.025 and obeying all constraints, c_3 can range between 0 and 0.9. Given p_0 and a value for c_3 we can derive $c_1 = c_2 = \frac{1 - 4p_0 - c_3}{4}$. The regional probabilities chosen for the experiments are summarised in Table S4 (including null values for when $c_3 = 0$), where we picked 6 equally spaced settings between 0.25 and 0.5 for the interaction effect c_3 in R_3.

	R_0	R_1	R_2	$R_{3[null]}$	$R_{3[0.25]}$	$R_{3[0.3]}$	$R_{3[0.35]}$	$R_{3[0.4]}$	$R_{3[0.45]}$	$R_{3[0.5]}$
p_0	0.25	0.125	0.125	0.025	0.025	0.025	0.025	0.025	0.025	0.025
c_1	0	0.25	0	0.225	0.1625	0.15	0.1375	0.125	0.1125	0.1
c_2	0	0	0.25	0.225	0.1625	0.15	0.1375	0.125	0.1125	0.1
c_3	0	1	1	0	0.25	0.3	0.35	0.4	0.45	0.5
p_1	0.25	0.375	0.125	0.25	0.1875	0.175	0.1625	0.15	0.1375	0.125
p_2	0.25	0.125	0.375	0.25	0.1875	0.175	0.1625	0.15	0.1375	0.125
p_3	0.25	0.375	0.375	0.475	0.6	0.625	0.65	0.675	0.7	0.725

Table S4. Approximate effect sizes and derived probabilities for each region of the interaction experiments. Region $R_3$is the only region that is varied across experiments, by increasing the magnitude of the interaction effect c_3 specified in square brackets.

As with the noise parameterisation, a data set of N observations can be generated in a few simple steps. Again, we add random effects $\boldsymbol{\zeta}$ and $\boldsymbol{\omega}$ from multidimensional uniform distributions as defined above:

1. Draw a uniform sample of N grid cell coordinates $\boldsymbol{w}_i \in \{1, \dots, a\} \times \{1, \dots, b\},\ i: 1, \dots, N$
2. For each grid cell coordinate $\boldsymbol{w}_i$ draw a sample $\boldsymbol{z}_i$ from $\mathrm{P}_k(\boldsymbol{Z}; \boldsymbol{\theta}_{interaction})$, where $\boldsymbol{w}_i \in R_k$ and $\boldsymbol{\theta}_{interaction}$ is the vector of combined regional parameters p_0, c_1, c_2 and c_3 for all four regions.

3. Obtain an observation $\boldsymbol{y}_i$ at location $\boldsymbol{x}_i$ by applying small amounts of random noise to $\boldsymbol{z}_i$ and $\boldsymbol{w}_i$:

$$\boldsymbol{y}_i = \boldsymbol{z}_i + \boldsymbol{\zeta}_i$$
$$\boldsymbol{x}_i = \boldsymbol{w}_i + \boldsymbol{\omega}_i$$

This completes our description of the synthetic data used to establish the face validity of GeoSPM.

S2.2 UK Biobank data

UK Biobank provides a large collection of health and genetic information for its prospective cohort of more than 500 000 participants recruited between 2006 and 2010 with assessment centres throughout Great Britain (https://www.ukbiobank.ac.uk/) [24].

We extracted a set of variables from UK Biobank in a region defined by a 35 km by 35 km square (spanning from 388000E, 423000N in the south-west corner to 269000E, 304000N in its north-east corner, in co-ordinates of the Ordnance Survey National Grid). The variables were sex (field 31), age (field 21022), body mass index (BMI, field 21001), household income (field 738) and the location of the participants (fields 20074 and 20075). Location information is based on the address to which the participants invitation was sent. Address verification and geo-coding was performed by UK Biobank using commercial software from Experian PLC and locations are provided at 100 metre and 1000 metre resolutions, the latter being the resolution available to us. All location co-ordinates use the Ordnance Survey reference. UK Biobank provides one or more temporal instances for certain fields. For such fields, the value of the earliest instance was chosen, which was the case for BMI and household income. In addition, ICD-10 and ICD-9 diagnosis codes were gathered from a separate hospital inpatient data table named HESIN_DIAG provided through field 41259. From these diagnosis codes we defined an indicator variable for type 2 diabetes, whose value was set to 1 whenever a participant had a record of either an ICD-10 code in block E11 ("type 2 diabetes mellitus") or at least one of a handful of relevant ICD-9 codes as specified in Table S7 in Supplemental Note. The number of participants with available data for all selected variables in the selected area of Birmingham was 18193, resulting in a collection of as many individual locations and associated individual observations that was used in the subsequently described analysis.

As a preliminary sanity check for the presence and degree of associativity, the diabetes indicator variable was entered as the response variable into a multiple Bayesian logistic regression model with a ridge prior. Sex, age, BMI, household income and the interaction between BMI and household functioned as predictors. Age, BMI and household income were centred at 0 and divided by their respective sample standard deviations. The interaction term was then formed as a simple multiplication. The model was evaluated by BayesReg version 1.9.1 [S1] in MATLAB. BayesReg uses a Markov Chain Monte Carlo (MCMC) Gibb's sampler. Posterior parameters were estimated from a single chain of 250000 samples (after a burn-in period of the same number of samples), of which only every 5th sample was used for computing the estimate. The posterior means of the regression coefficients and their credible intervals were as follows:

Predictor	Coefficient Posterior Mean ± SD	95% Credible Interval	t-Statistic	ESS
Sex	0.748 ± 0.054	(0.642 to 0.855)	13.79	82.2
Age	0.313 ± 0.029	(0.257 to 0.371)	10.73	83.5
BMI	0.719 ± 0.025	(0.670 to 0.769)	28.64	71.5
Household Income	-0.344 ± 0.036	(-0.416 to -0.274)	-9.50	61.9
BMI x Household Income	0.053 ± 0.028	(-0.001 to 0.108)	1.93	73.0

Table S5. Results of the preliminary Bayesian logistic ridge regression analysis of the UK Biobank diabetes data set extracted for Birmingham.

The results showed that there is a reasonably strong association between type 2 diabetes and all main terms, but evidence for an interaction between BMI and household income appears to be weak. On the basis of this preliminary analysis, we directed our attention to the spatial variability of diabetes and the question of how much of this spatial variability is driven by the other variables. We defined a progression of four models, listed in Table S6.

Model	Type 2 Diabetes	Sex	Age	BMI	Household Income	BMI x Household Income
1	■	–	–	–	–	–
2	■	■	■	■	–	–
3	■	■	■	■	■	–
4	■	■	■	■	■	■

Table S6. The four GeoSPM models used for the Birmingham data from UK Biobank.

It is important to keep in mind that unlike in this preliminary analysis, in these GeoSPM models, type 2 diabetes is no longer a response variable but an explanatory or independent variable, which means its effect is marginalised relative to the other variables in each model. By applying a single colour map to all regression coefficient maps across models, the intensity and nature of topological changes—in the marginalised contribution of each variable—become visible, not only within a single model but over the ensemble of four models. Similarly, changes in the extent and location of significant areas, due to the addition of variables as we move from one model to the next, allow us to assess patterns of spatial variability. Lastly, using intersections between significant areas of multiple variables, we can identify areas of significant *conjunctions* between those variables[S2].

S2.3 Kriging

Kriging[S3] is an ensemble of linear least-squares regression techniques for predicting the value of a random field at an unsampled location from observations at other locations. It is commonly used when interpolating spatially-referenced point data over a surface and provides a measure of the

uncertainty in its predictions. In statistics and machine learning, kriging is essentially an application of multivariate Gaussian process prediction. Crucially, kriging requires an explicit model of the spatial covariance and cross-covariance of the data, which needs to be chosen *a priori*. As the random field is generally assumed to be second-order stationary and isotropic, the covariance can be expressed as a function of the Euclidean distance between a pair of points, independently of their actual location in the spatial domain. A theoretical variogram is the quasi-dual form of a covariance model (it is slightly more generic in some situations). An overview of some common theoretical variograms is shown in Figure S6. Parameters required by the selected model are estimated from the data and substituted for the true values when computing the predictions. The covariance and cross-covariance model we used for all kriging predictions presented in the main text is the family of Matérn functions[S4] together with an added "nugget" component. The Matérn model exhibits adaptable smoothness controlled by a parameter κ and is recommended as a sensible default choice in the literature[S5], [S6]. The nugget component adds a discontinuous jump to the covariance function at coincident points and captures variance due to measurement error. Its relative strength is specified by a single numeric parameter. Additional parameters of the Matérn model are the sill, which determines its contribution to the covariance, as well as the range which reflects its spatial scale. For the main results reported in Figures 3 and 4, as well as Figures S7–S11, we left parameter κ fixed at its default gstat setting of 0.5, whereas for the extended comparison of kriging covariance models reported in Sections S3.4, S3.5, S3.6 and S3.7), κ was estimated within a pre-specified range of [0.1, 5].

In cases where the experimental data contained several variables, gstat estimated a linear model of coregionalization (LCM), which expressed all required auto- and cross-covariances as linear combinations of a Matérn function and a nugget component. The range parameter is constrained by gstat to be the same for all covariances in the LCM and was estimated from the first variable in the data prior to estimating the LCM. We configured gstat to use ordinary (co-)kriging with a constant but unknown mean in a global search window.

In addition to the Matérn model, we present a wider comparison of results with the kriging models shown in Figure S6 in Section S3.

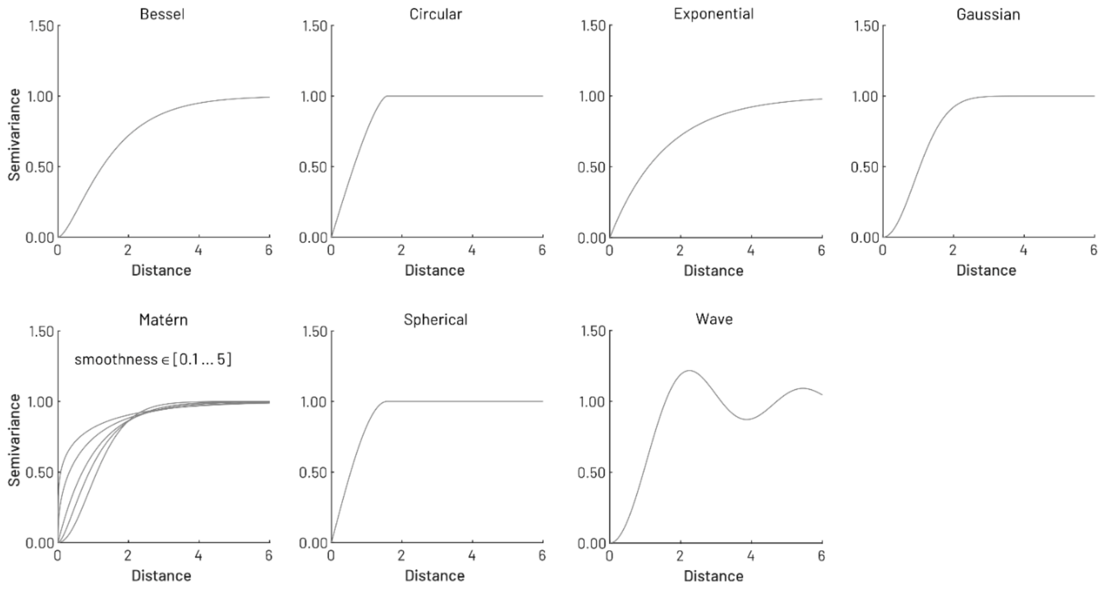

Figure S6. A range of common theoretical variograms to be fitted to the synthetic model data in the kriging experiments.

S3 Additional Results

S3.1 Synthetic Experiment Results for Univariate Models

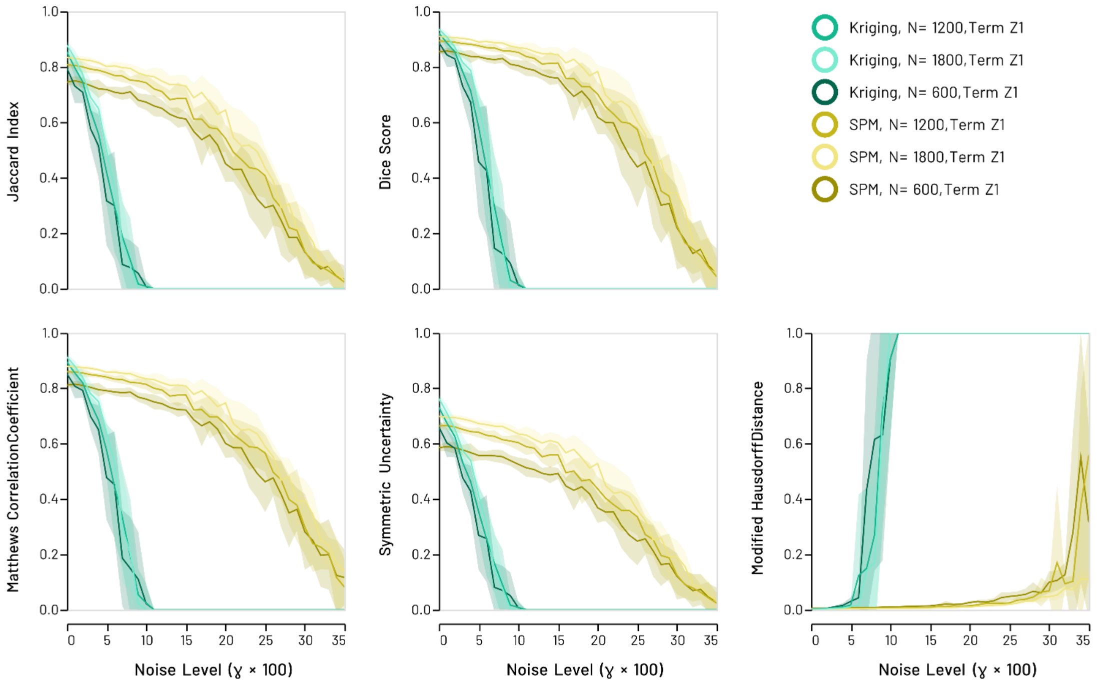

Figure S7. Synthetic univariate snowflake models: Recovery scores for the single GeoSPM and kriging model term in the low (N = 600), middle (N = 1200) and high (N = 1800) sampling regime. Lines denote the mean score across 10 random model realisations, shaded areas its standard deviation to either side of the mean. GeoSPM degrades more slowly and gracefully as noise increases compared with kriging.

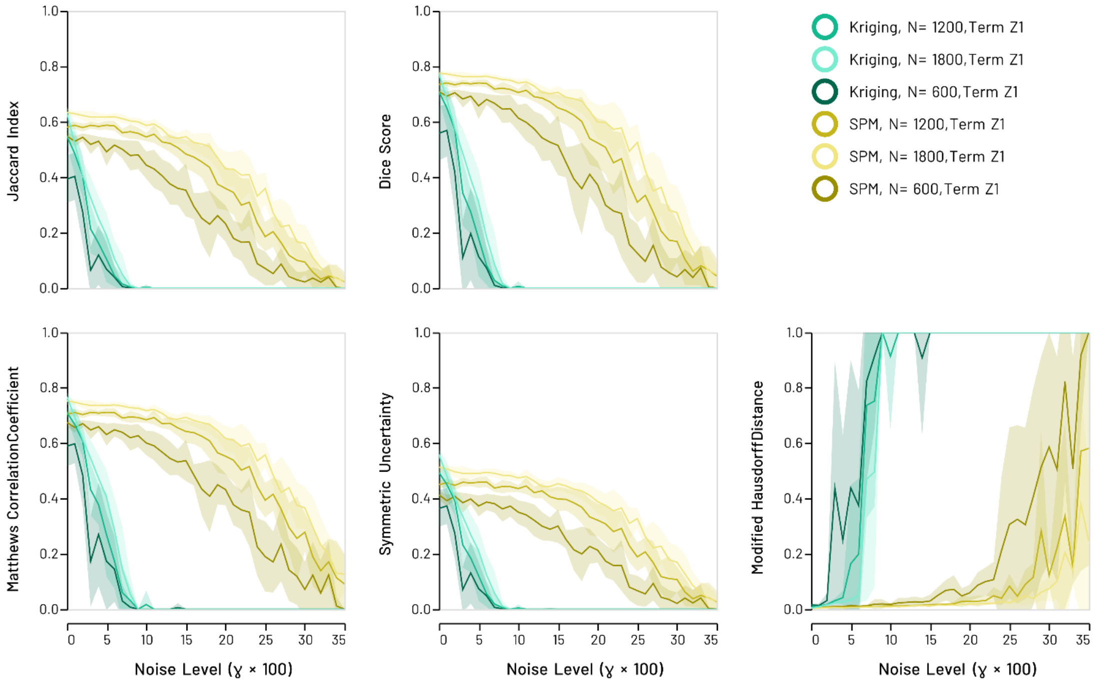

Figure S8. Synthetic univariate anti-snowflake models: Recovery scores for the single GeoSPM and kriging model term in the low (N = 600), middle (N = 1200) and high (N = 1800) sampling regime. Lines denote the mean score across 10 random model realisations, shaded areas its standard deviation to either side of the mean. Areas of overlapping performance are identified by additive shading. GeoSPM degrades more slowly and gracefully as noise increases compared with kriging.

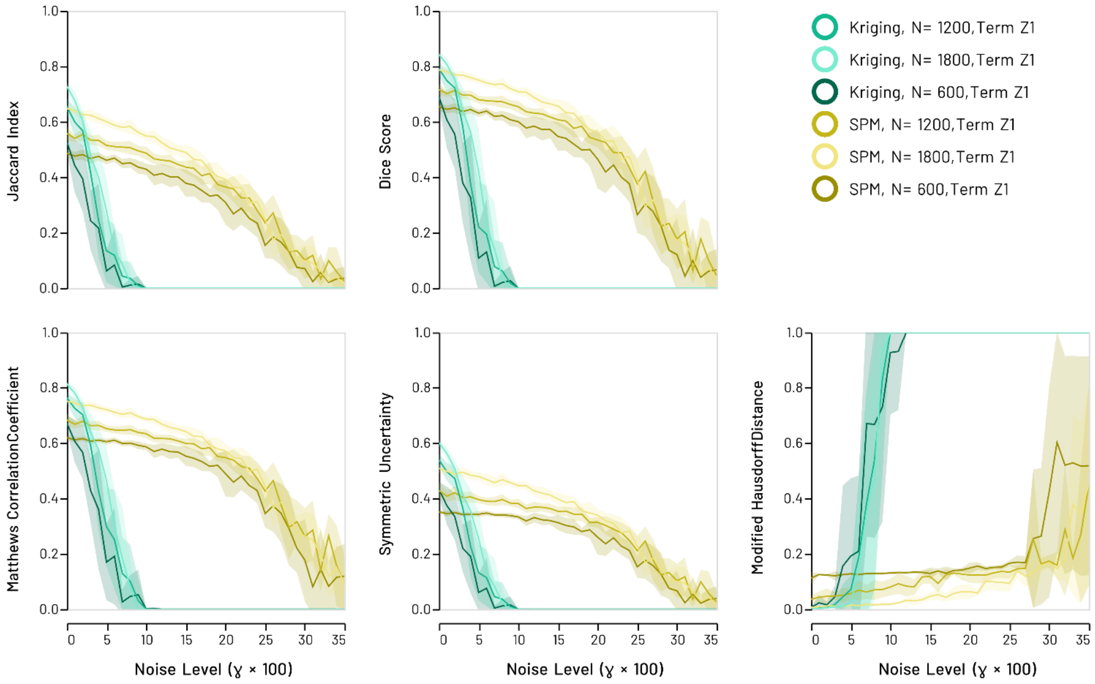

Figure S9. Synthetic univariate snowflake field models: Recovery scores for the single SPM and kriging model term in the low (N = 600), middle (N = 1200) and high (N = 1800) sampling regime. Lines denote the mean score across 10 random model realisations, shaded areas its standard deviation to either side of the mean. Areas of overlapping performance are identified by additive shading. GeoSPM degrades more slowly and gracefully as noise increases compared with kriging.

S3.2 Synthetic Experiment Results for Term Z_2 of the Bivariate Models

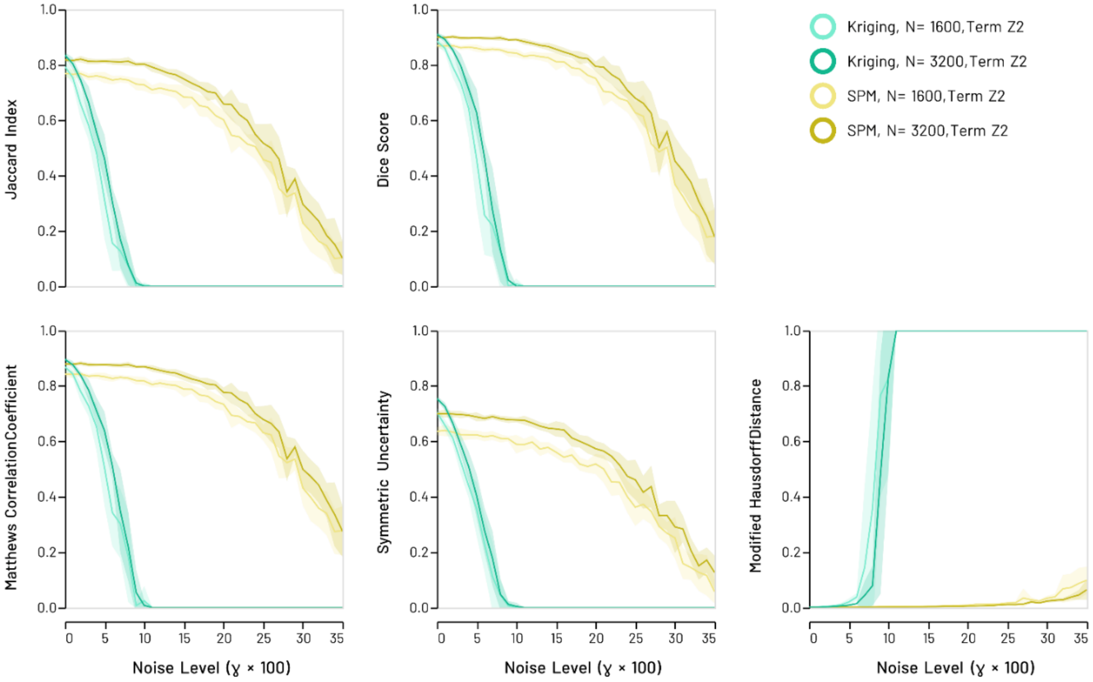

Figure S10. Synthetic snowflake models: Recovery scores for GeoSPM and kriging model term Z_2 in the low (N = 1600) and high (N = 3200) sampling regime. Lines denote the mean score across 10 random model realisations, shaded areas its standard deviation to either side of the mean. Areas of overlapping performance are identified by additive shading. GeoSPM degrades more slowly and gracefully as noise increases compared to kriging.

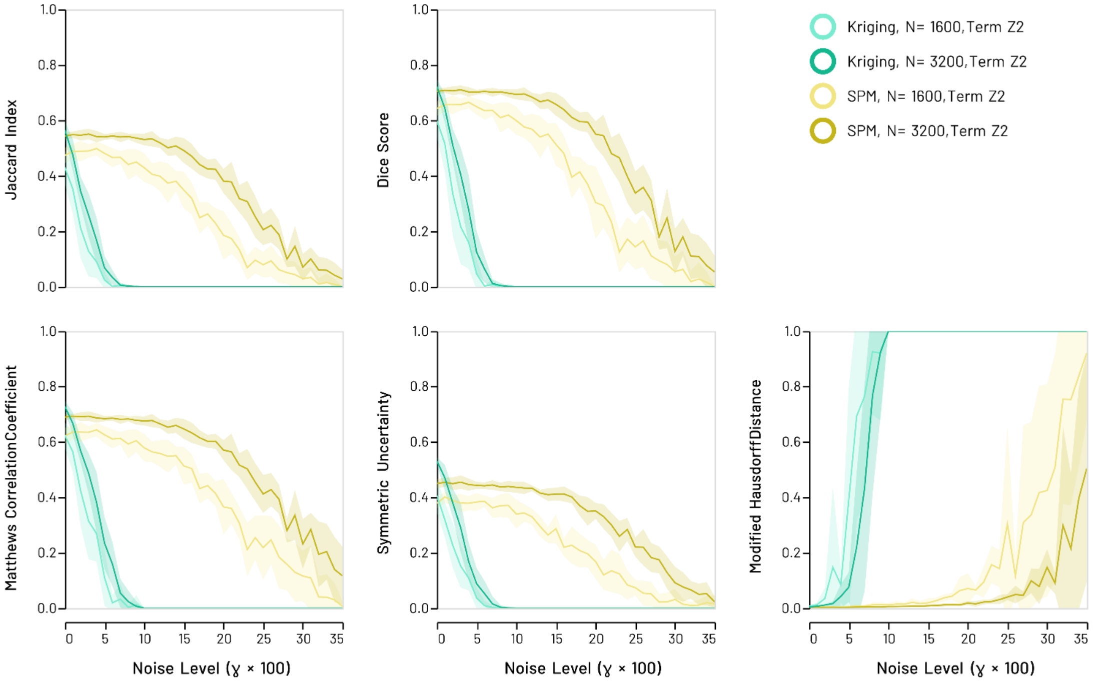

Figure S11. Synthetic anti-snowflake models: Recovery scores for GeoSPM and kriging model term Z_2 in the low (N = 1600) and high (N = 3200) sampling regime. Lines denote the mean score across 10 random model realisations, shaded areas its standard deviation to either side of the mean. Areas of overlapping performance are identified by additive shading. GeoSPM degrades more slowly and gracefully as noise increases compared with kriging.

S3.3 Synthetic Experiment Results for Kriging When Averaging Coincident Observations

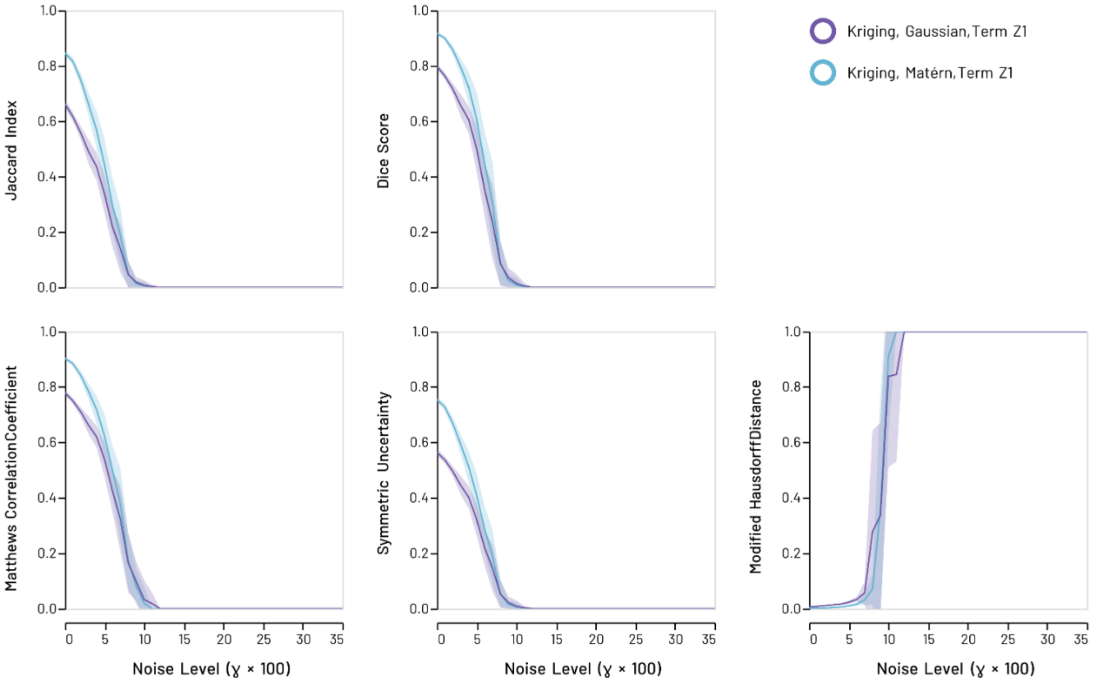

Figure S12. Synthetic bivariate snowflake models: Recovery scores for kriging model term Z_1 with a Matérn covariance function (blue) and a Gaussian covariance function (purple) in the high (N = 3200) sampling regime. Lines denote the mean score across 10 random model realisations, shaded areas its standard deviation to either side of the mean. In both cases coincident observations were *averaged* and reduced to one instead of adding a small amount of random noise to their locations as before. However, this did not change the performance in any meaningful way when compared with a Matérn covariance function with random noise added (as shown in Figure 3): That curve is almost identical to the averaged version displayed here in blue and was therefore left out. The Gaussian covariance function performs slightly worse than the Matérn covariance. This leads us to believe that kriging performance is not improved in our experiments by choosing a different coincident observation regime or covariance function (which is confirmed by the results presented in section S3.4)

S3.4 Extended Synthetic Experiment Results for Term $\boldsymbol{Z_1}$ of the Bivariate Model

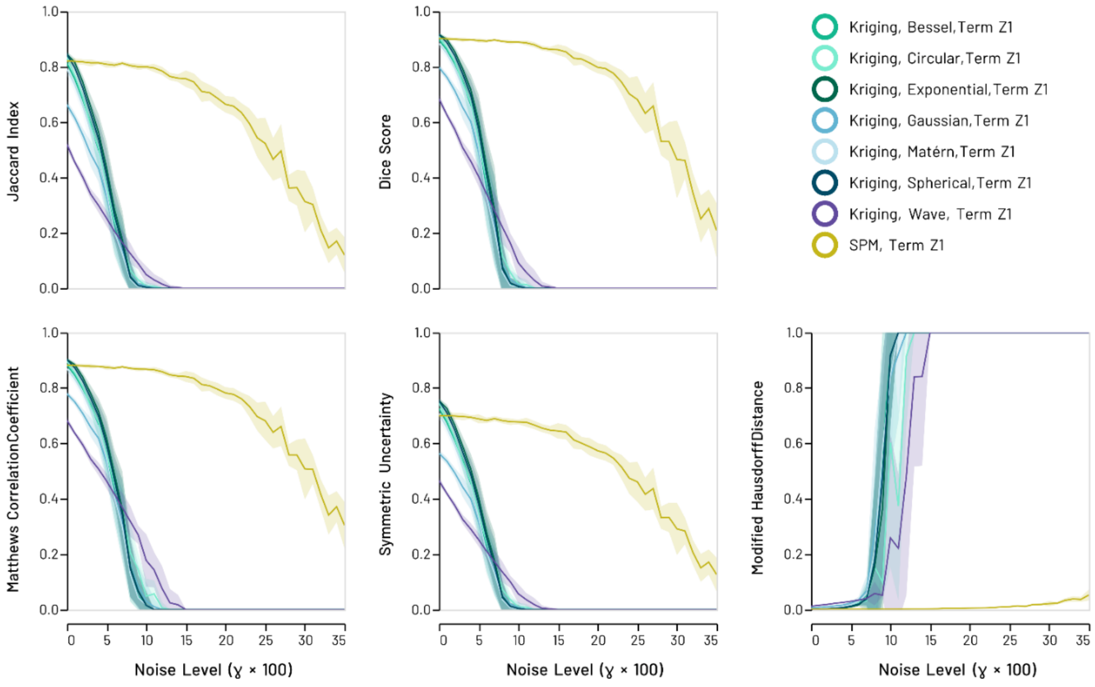

Figure S13. Synthetic snowflake models: Recovery scores for various kriging models *with* a nugget term in comparison with SPM for term Z_1 and the high sampling regime (N = 3200).

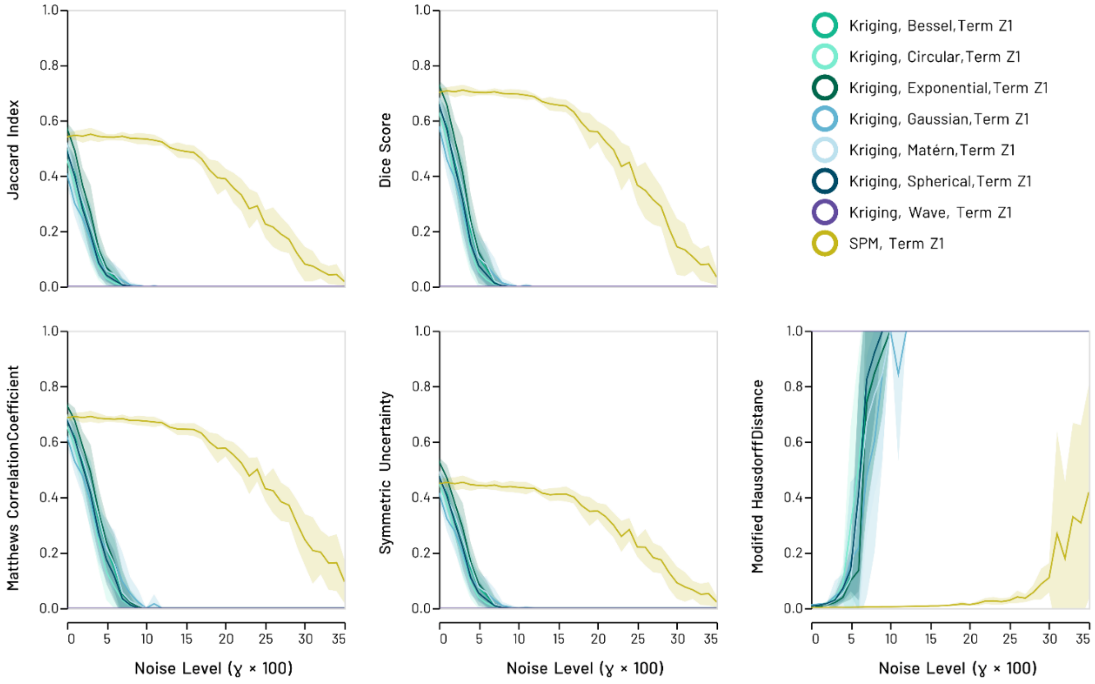

Figure S14. Synthetic anti-snowflake models: Recovery scores for various kriging models *with* a nugget term in comparison with SPM for term Z_1 and the high sampling regime (N = 3200).

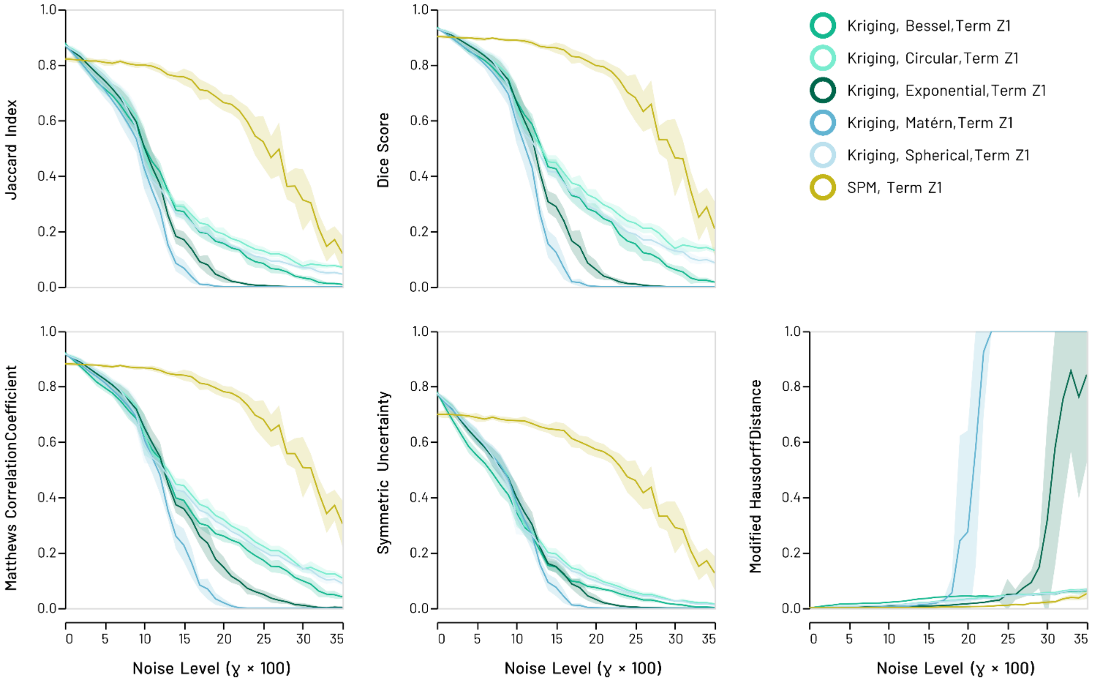

Figure S15. Synthetic snowflake models: Recovery scores for various kriging models *without* a nugget term in comparison with SPM for term Z_1 and the high sampling regime (N = 3200).

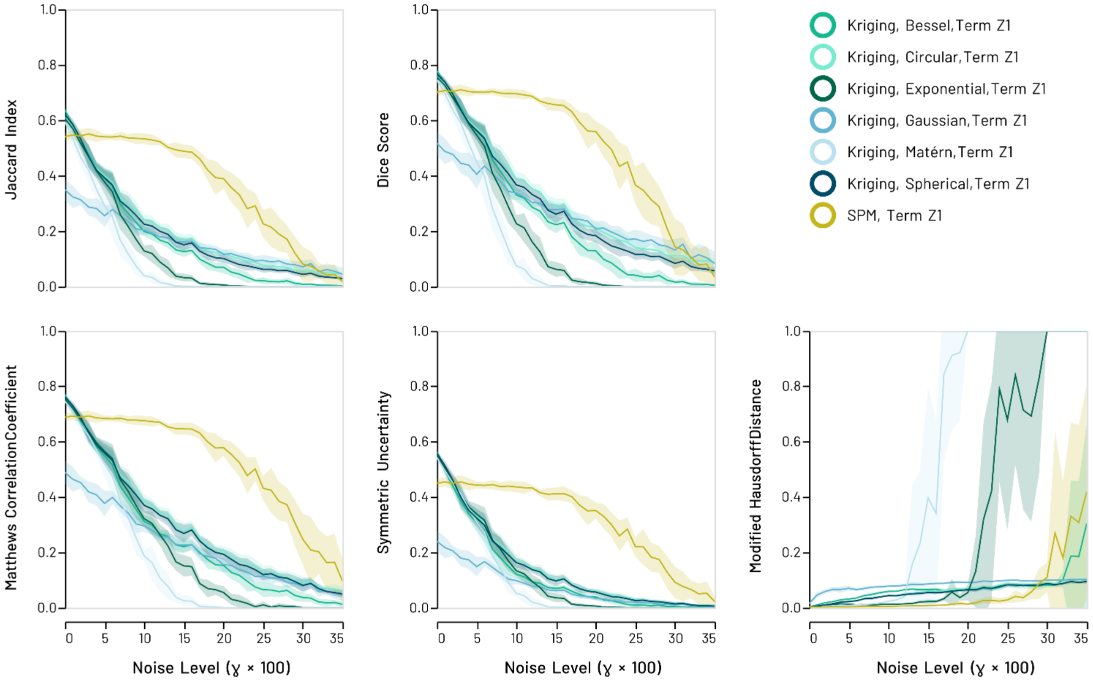

Figure S16. Synthetic anti-snowflake models: Recovery scores for various kriging models *without* a nugget term in comparison with SPM for term Z_1 and the high sampling regime (N = 3200).

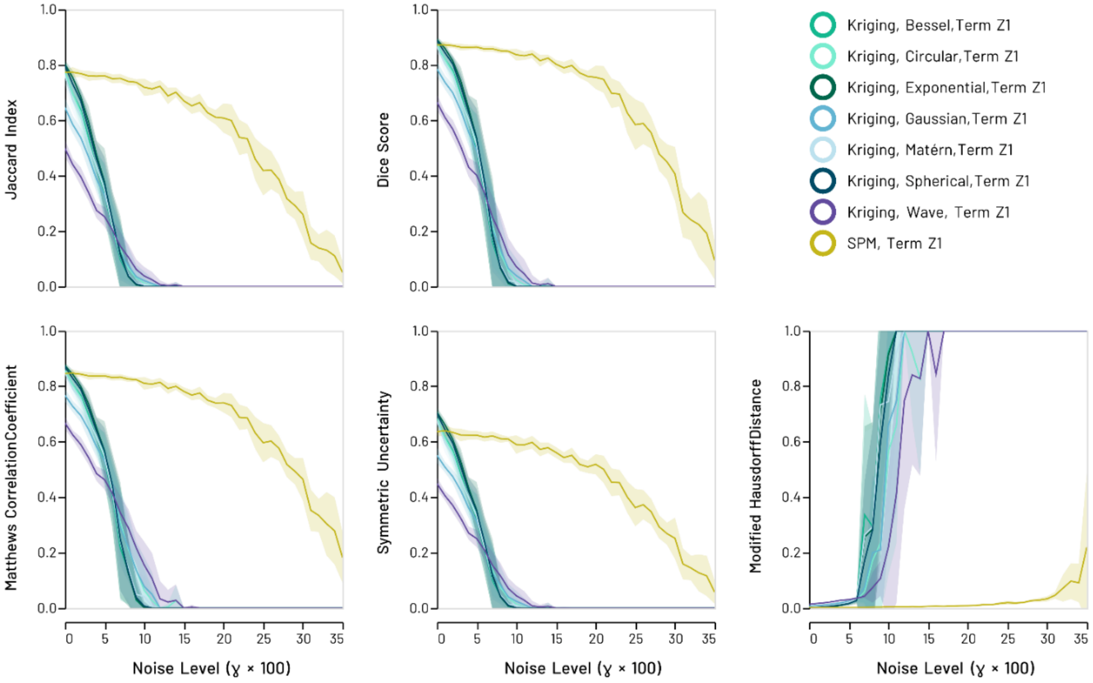

Figure S17. Synthetic snowflake models: Recovery scores for various kriging models *with* a nugget term in comparison with SPM for term Z_1 and the low sampling regime (N = 1600).

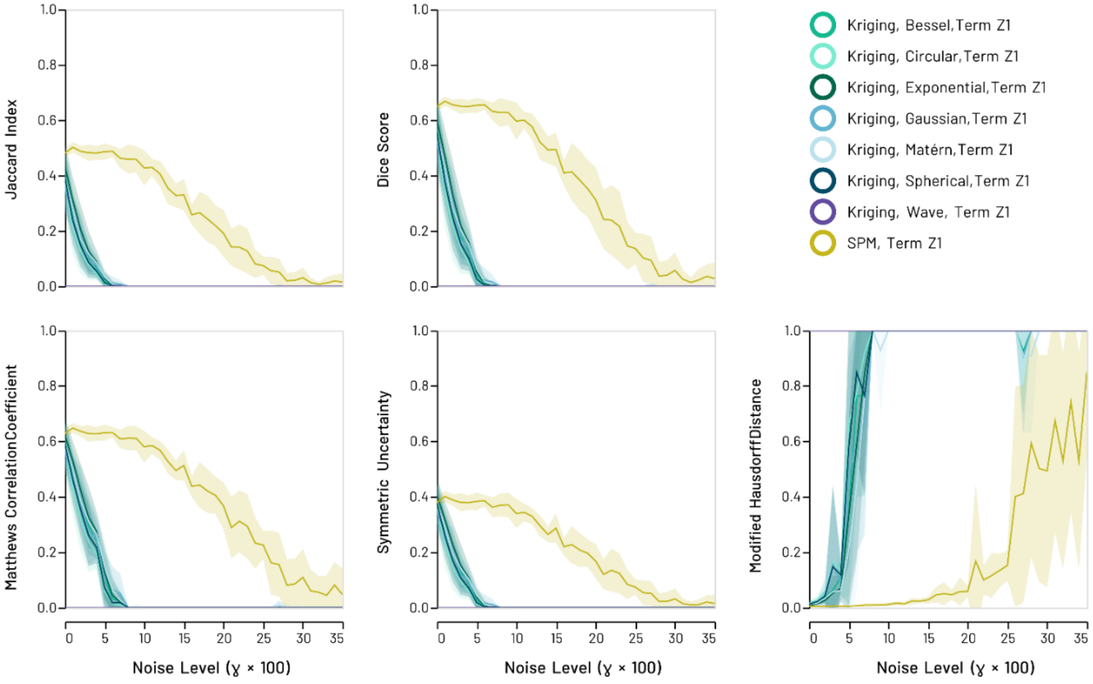

Figure S18. Synthetic anti-snowflake models: Recovery scores for various kriging models *with* a nugget term in comparison with SPM for term Z_1 and the low sampling regime (N = 1600).

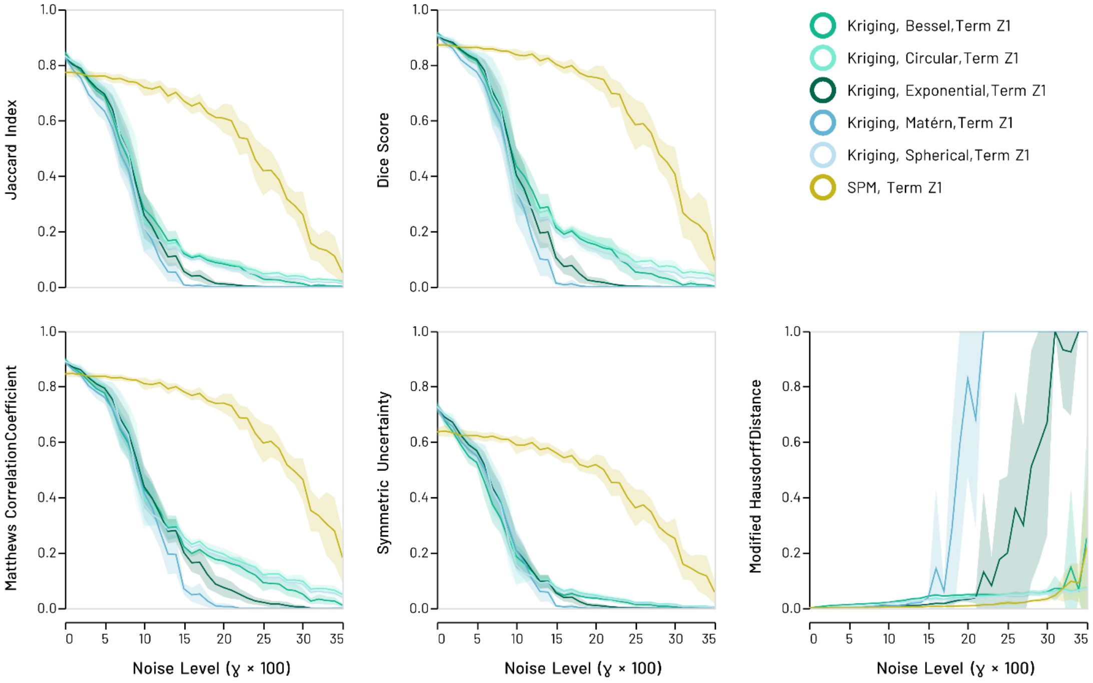

Figure S19. Synthetic snowflake models: Recovery scores for various kriging models *without* a nugget term in comparison with SPM for term Z_1 and the low sampling regime (N = 1600).

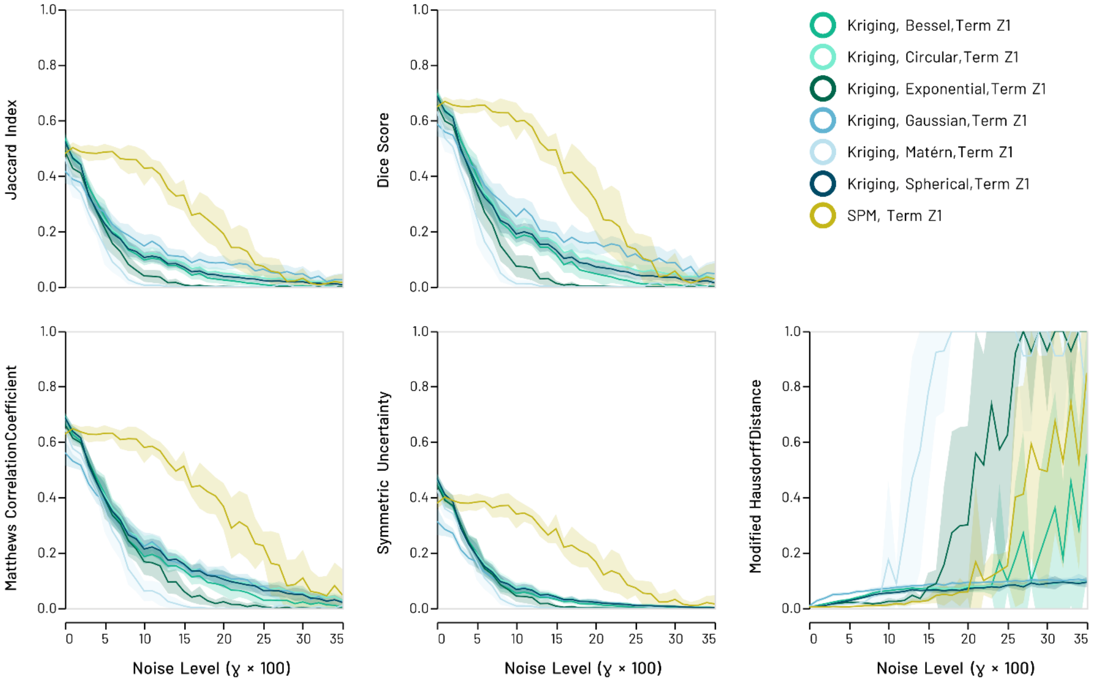

Figure S20. Synthetic anti-snowflake models: Recovery scores for various kriging models *without* a nugget term in comparison with SPM for term Z_1 and the low sampling regime (N = 1600).

S3.5 Extended Synthetic Experiment Kriging Recoveries for Term $\boldsymbol{Z_1}$ of the Bivariate Model

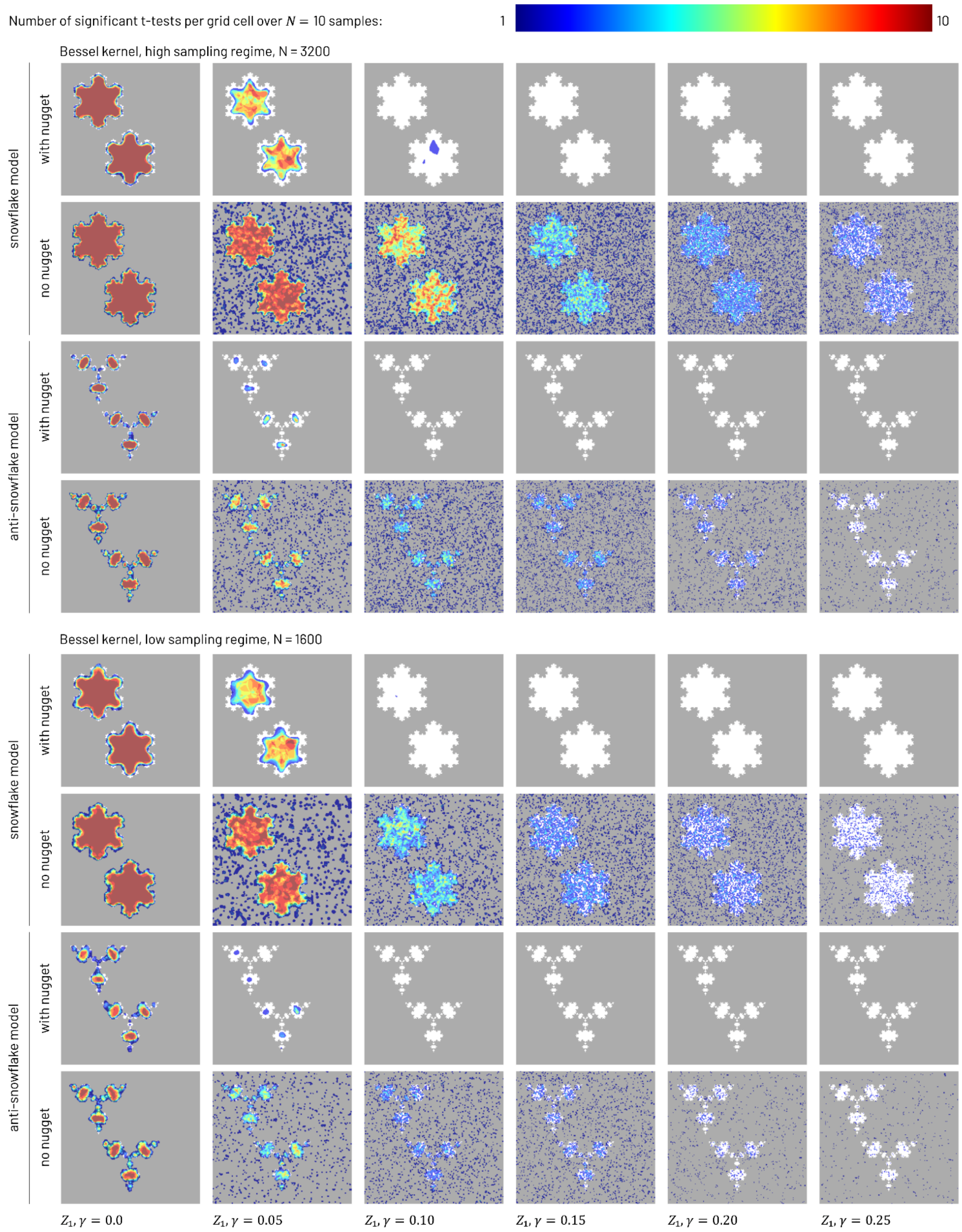

Figure S21. Kriging recoveries of term Z_1 for the Bessel kernel. Columns represent increasing noise levels. Each row shows a combination of the synthetic model used and whether a nugget component was included in the variogram.

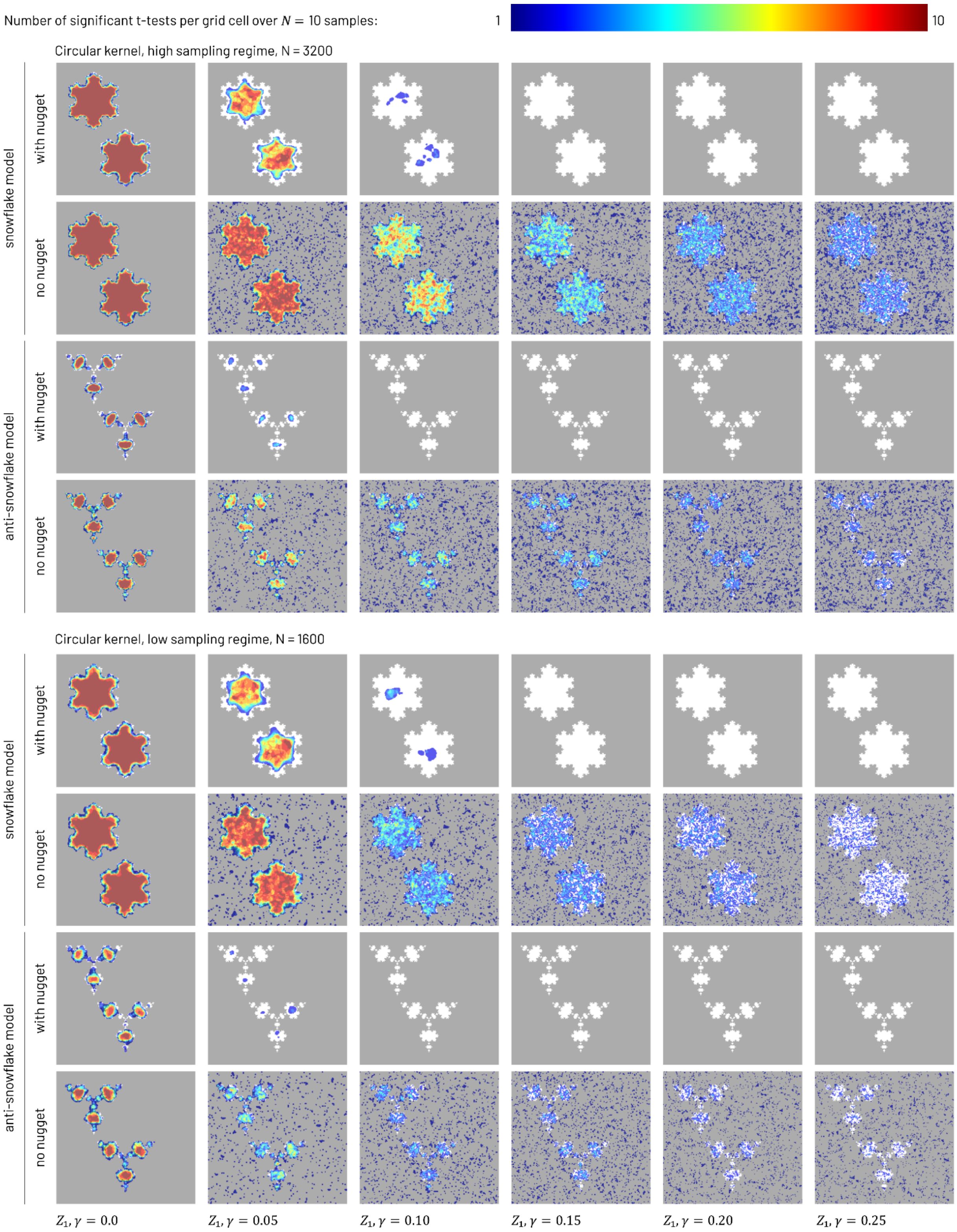

Figure S22. Kriging recoveries of term Z_1 for the circular kernel. Columns represent increasing noise levels. Each row shows a combination of the synthetic model used and whether a nugget component was included in the variogram.

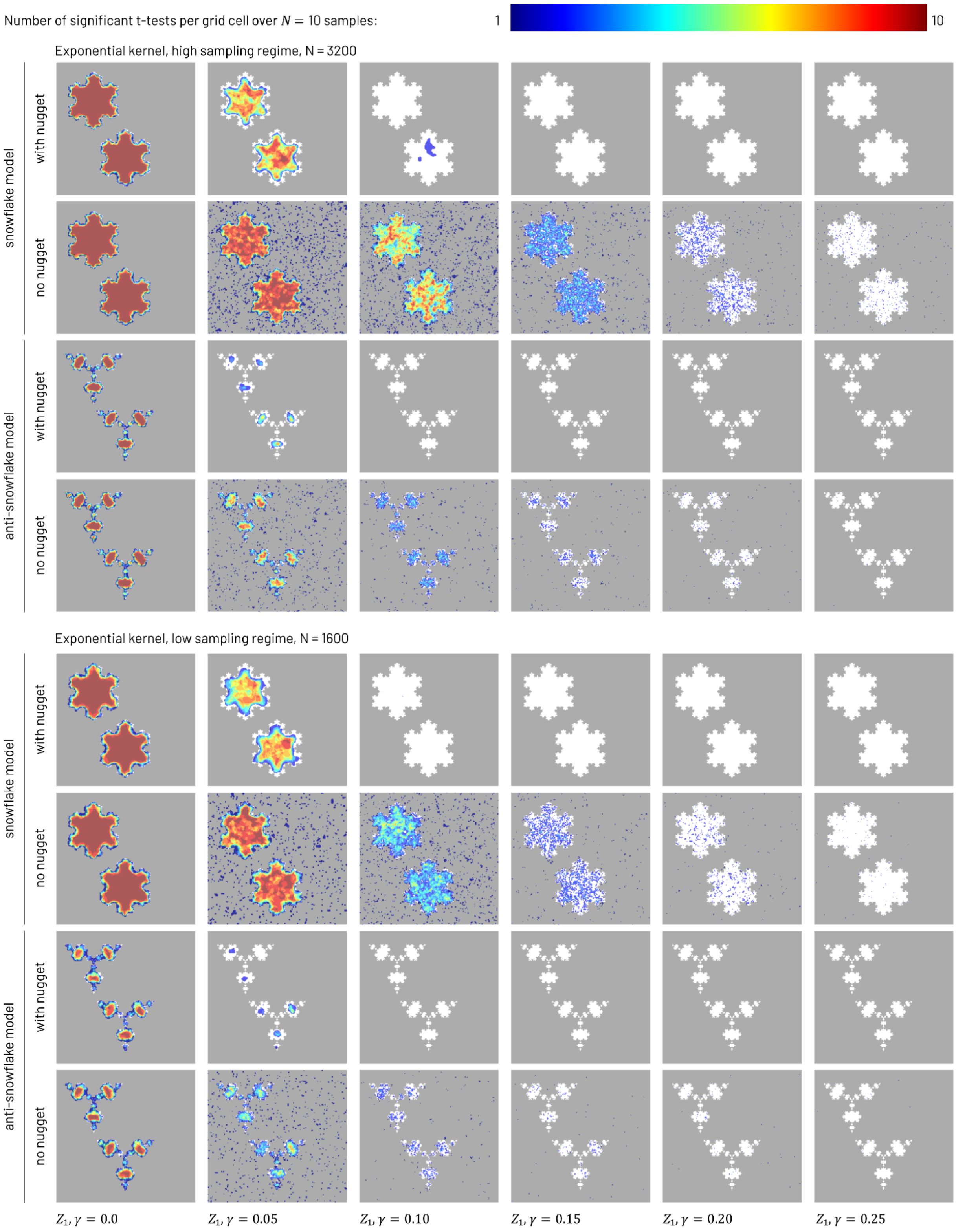

Figure S23. Kriging recoveries of term Z_1 for the exponential kernel. Columns represent increasing noise levels. Each row shows a combination of the synthetic model used and whether a nugget component was included in the variogram.

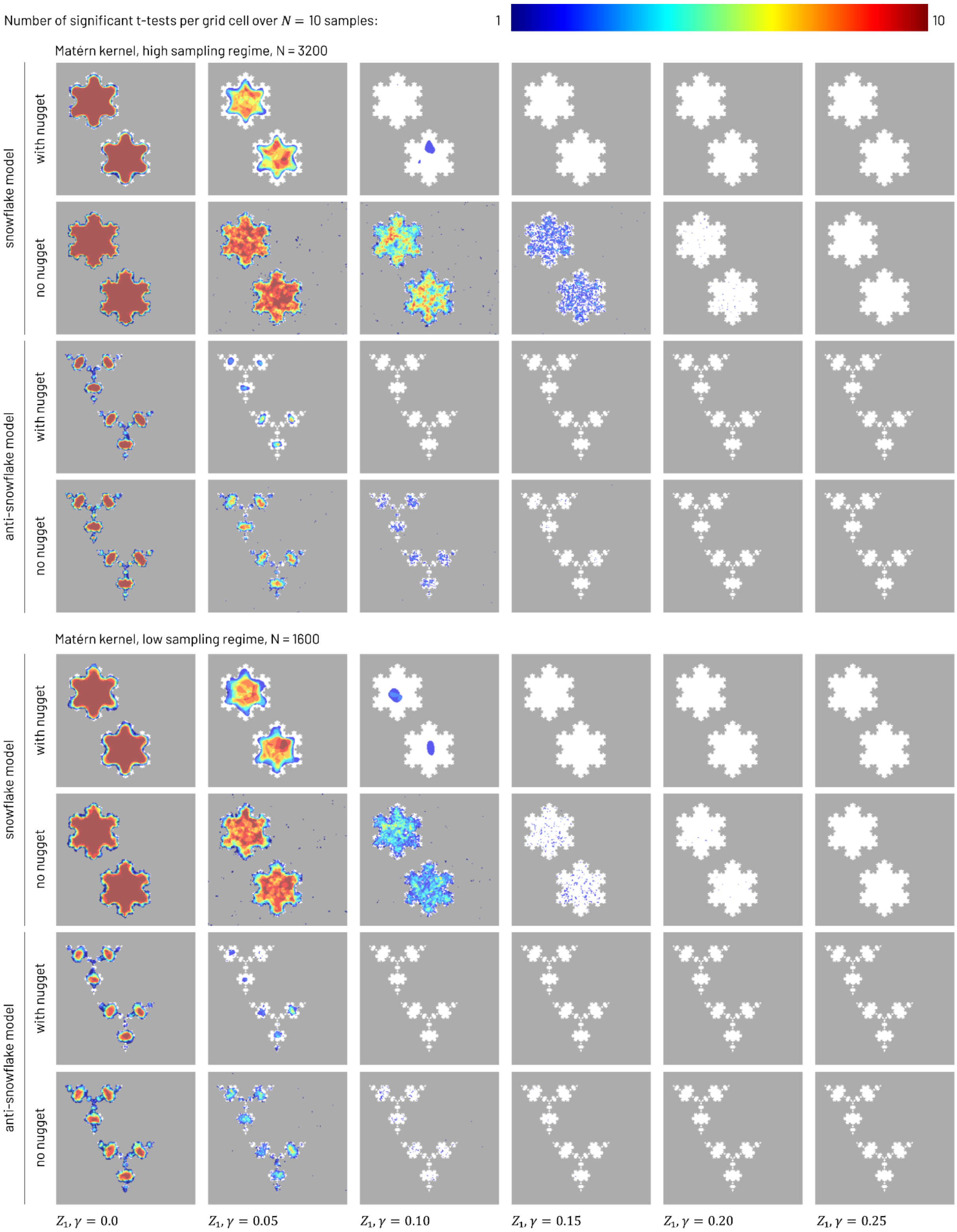

Figure S24. Kriging recoveries of term Z_1 for the Matérn kernel. Columns represent increasing noise levels. Each row shows a combination of the synthetic model used and whether a nugget component was included in the variogram.

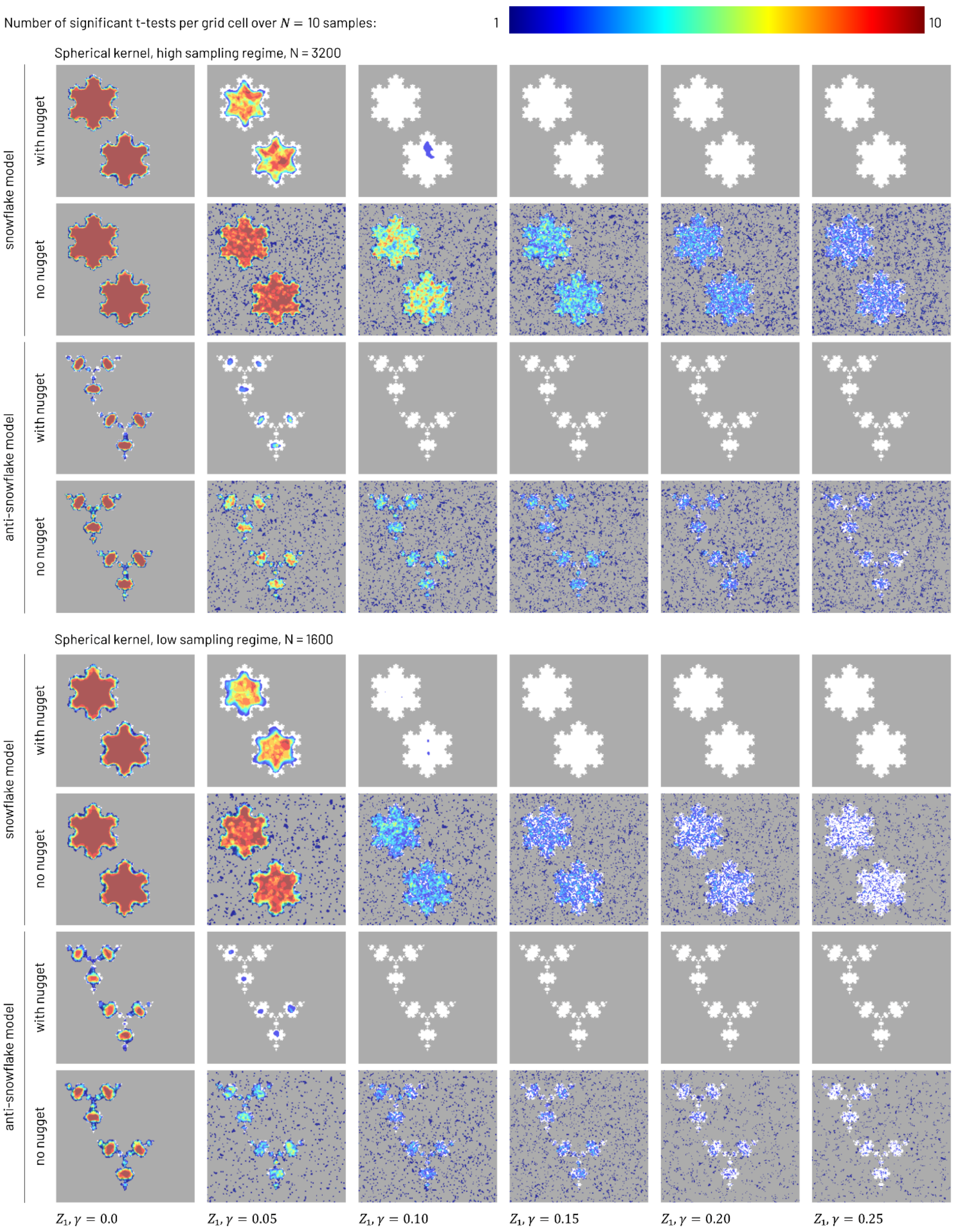

Figure S25. Kriging recoveries of term Z_1 for the spherical kernel. Columns represent increasing noise levels. Each row shows a combination of the synthetic model used and whether a nugget component was included in the variogram.

S3.6 Summary of Kriging Parameters for the Extended Synthetic Experiments

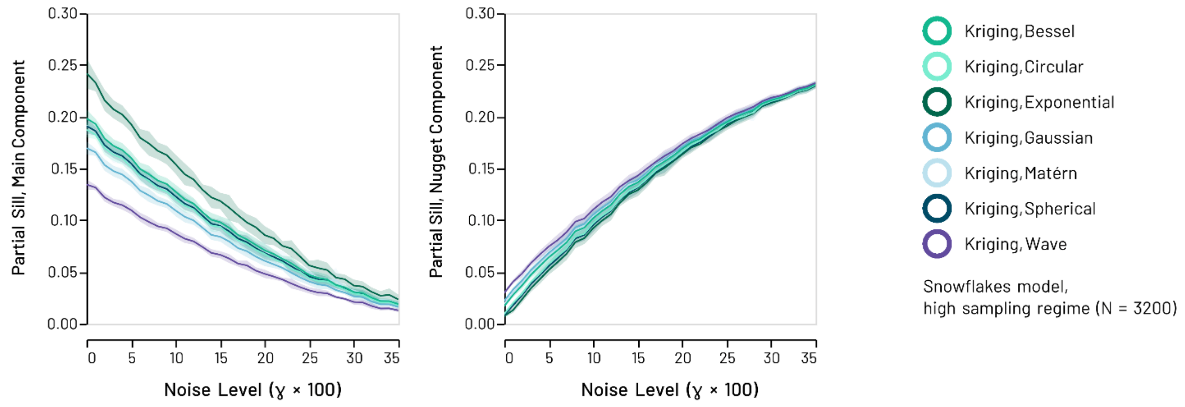

Figure S26. Synthetic snowflakes model: Estimated partial sill parameters for the main (left) and nugget (right) variogram components in the high sampling regime (N = 3200). Lines denote the mean estimate across 10 random model realisations, shaded areas its standard deviation to either side of the mean.

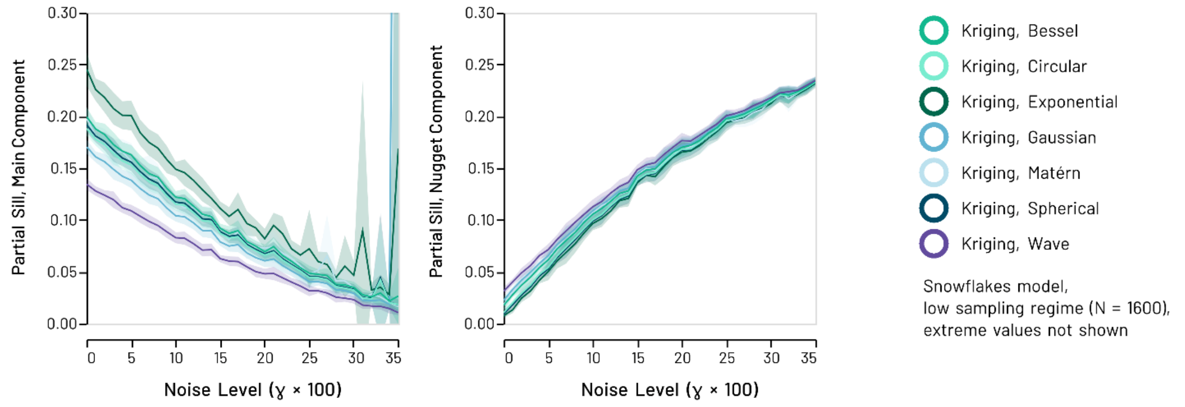

Figure S27. Synthetic snowflakes model: Estimated partial sill parameters for the main (left) and nugget (right) variogram components in the low sampling regime (N = 1600). Lines denote the mean estimate across 10 random model realisations, shaded areas its standard deviation to either side of the mean. At high levels of noise, estimates for some main components become unreliable, resulting in extreme values.

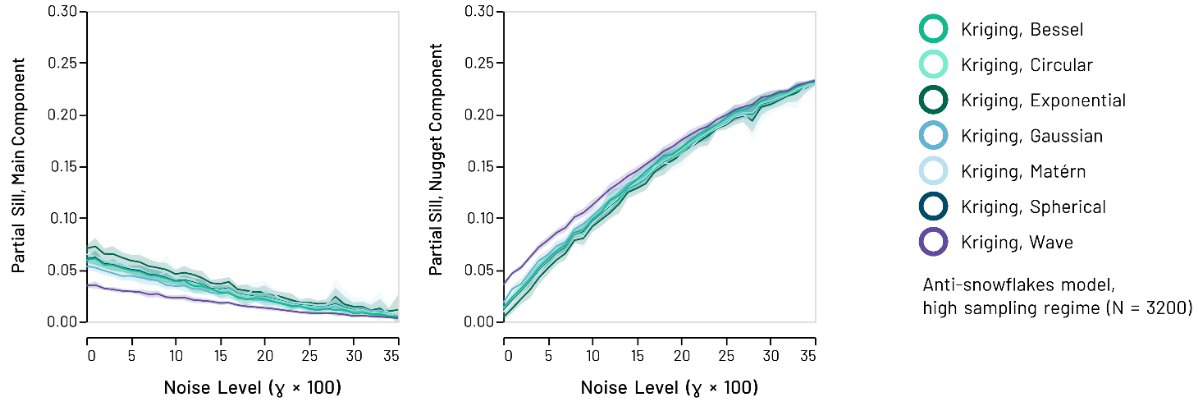

Figure S28. Synthetic anti-snowflakes model: Estimated partial sill parameters for the main (left) and nugget (right) variogram components in the high sampling regime (N = 3200). Lines denote the mean estimate across 10 random model realisations, shaded areas its standard deviation to either side of the mean.

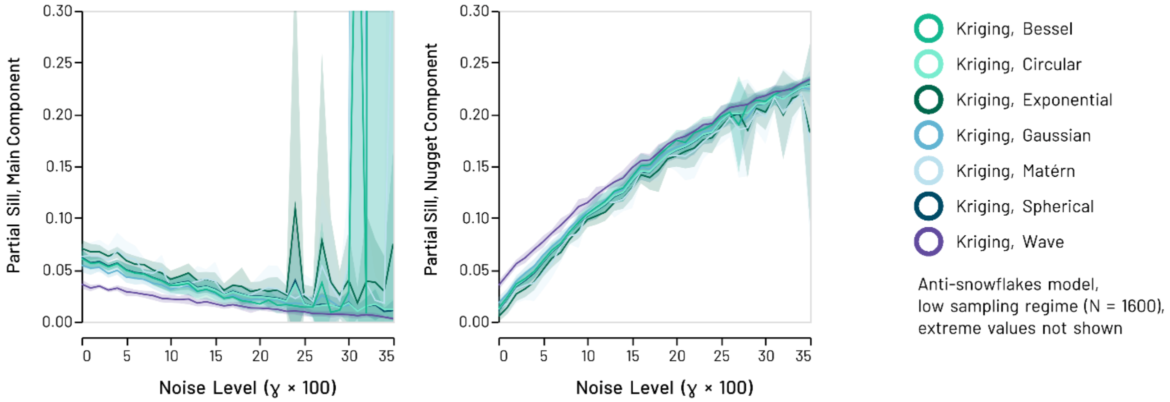

Figure S29. Synthetic anti-snowflakes model: Estimated partial sill parameters for the main (left) and nugget (right) variogram components in the low sampling regime (N = 1600). Lines denote the mean estimate across 10 random model realisations, shaded areas its standard deviation to either side of the mean. At high levels of noise, estimates for some main components become unreliable, resulting in extreme values.

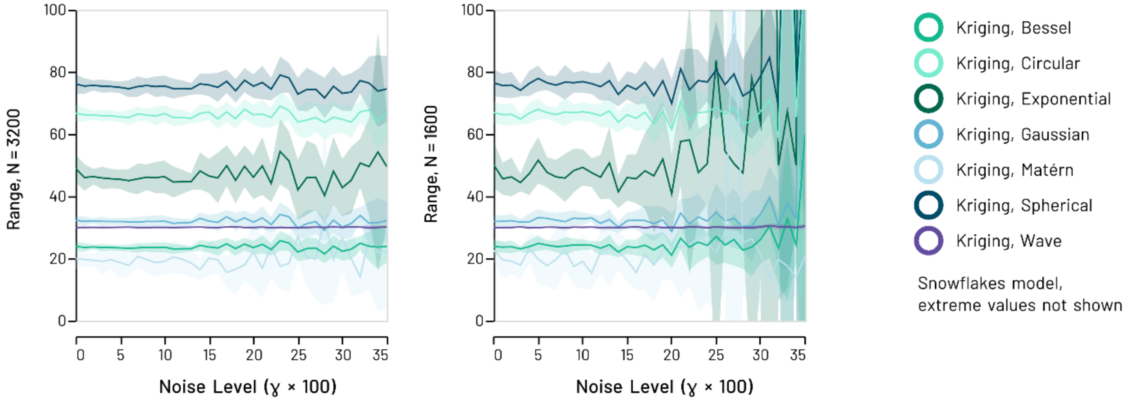

Figure S30. Synthetic snowflakes model: Estimated range parameters for the main variogram component in the high sampling regime (N = 3200, left) and low sampling regime (N = 1600, right). Lines denote the mean estimate across 10 random model realisations, shaded areas its standard deviation to either side of the mean. At high levels of noise, estimates unreliable, resulting in extreme values.

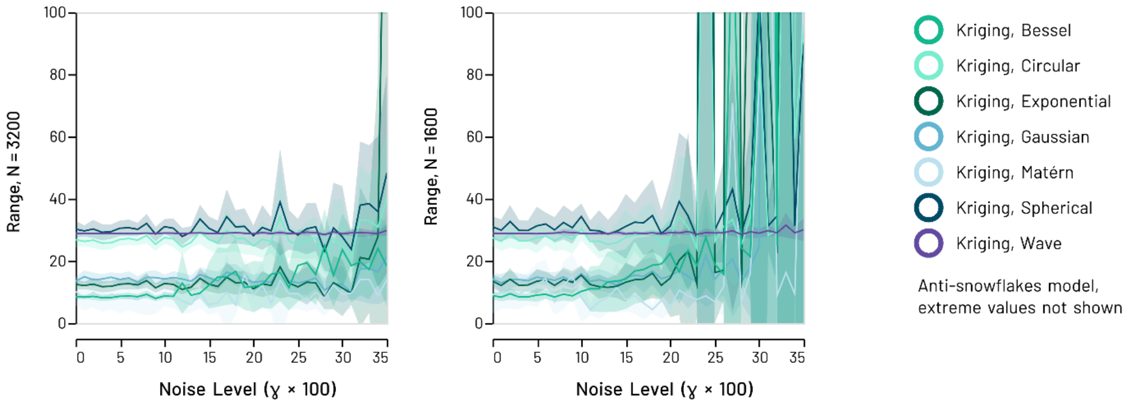

Figure S31. Synthetic anti-snowflakes model: Estimated range parameters for the main variogram component in the high sampling regime (N = 3200, left) and low sampling regime (N = 1600, right). Lines denote the mean estimate across 10 random model realisations, shaded areas its standard deviation to either side of the mean. At high levels of noise, estimates unreliable, resulting in extreme values.

S3.7 Summary of Kriging Variograms for the Extended Synthetic Experiments

S3.7.1 Bessel Kernel

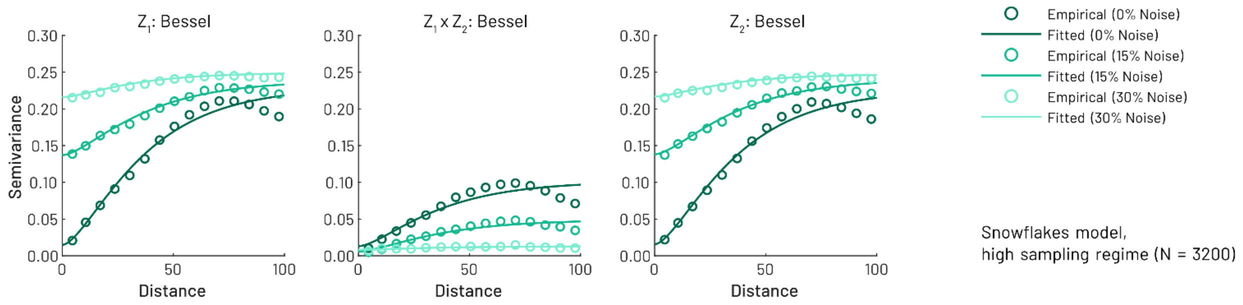

Figure S32. Synthetic snowflakes model: Empirical semivariograms and fitted Bessel kernel *with* a nugget component at three different noise levels for terms Z_1 and Z_2 and their cross-covariance in the high sampling regime (N = 3200) for a single run of the experiments.

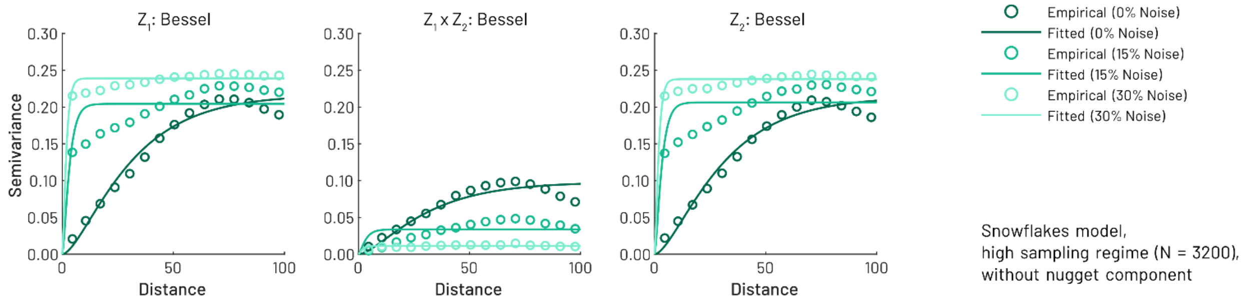

Figure S33. Synthetic snowflakes model: Empirical semivariograms and fitted Bessel kernel *without* a nugget component at three different noise levels for terms Z_1 and Z_2 and their cross-covariance in the high sampling regime (N = 3200) for a single run.

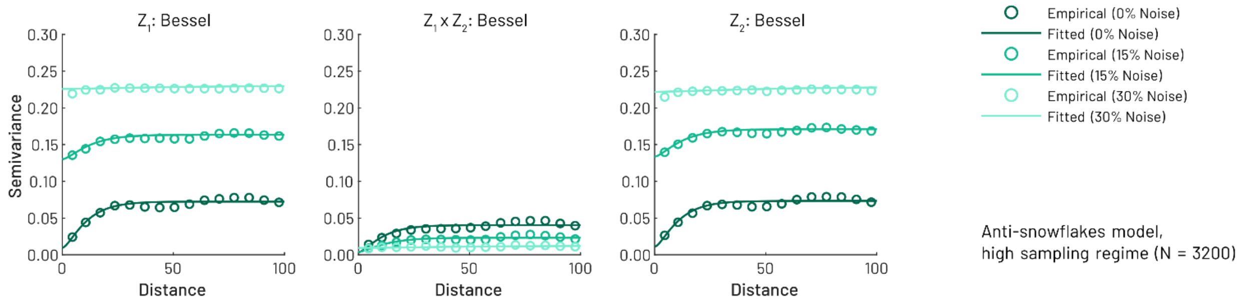

Figure S34. Synthetic anti-snowflakes model: Empirical semivariograms and fitted Bessel kernel *with* a nugget component at three different noise levels for terms Z_1 and Z_2 and their cross-covariance in the high sampling regime (N = 3200) for a single run.

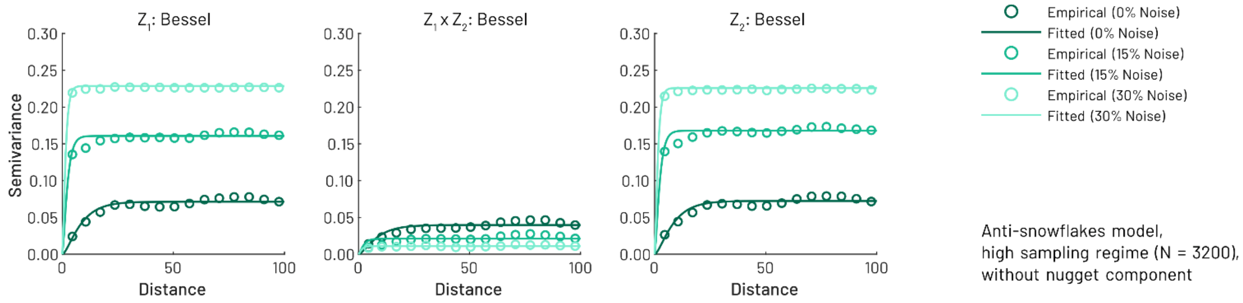

Figure S35. Synthetic anti-snowflakes model: Empirical semivariograms and fitted Bessel kernel *without* a nugget component at three different noise levels for terms Z_1 and Z_2 and their cross-covariance in the high sampling regime (N = 3200) for a single run.

S3.7.2 Circular Kernel

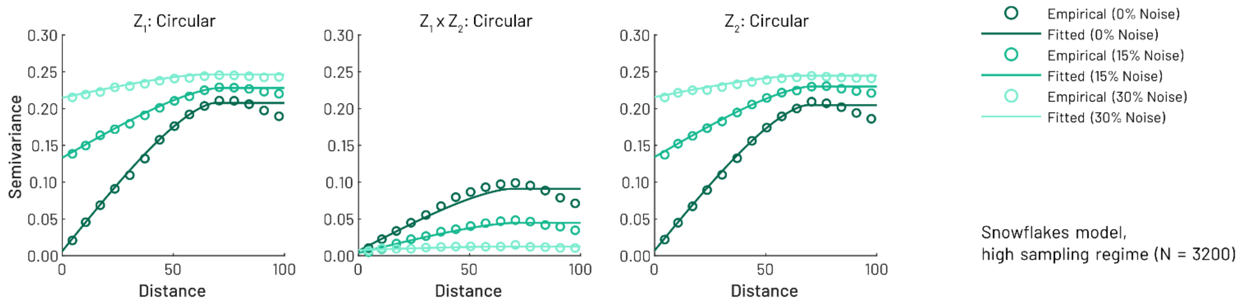

Figure S36. Synthetic snowflakes model: Empirical semivariograms and fitted circular kernel *with* a nugget component at three different noise levels for terms Z_1 and Z_2 and their cross-covariance in the high sampling regime (N = 3200) for a single run.

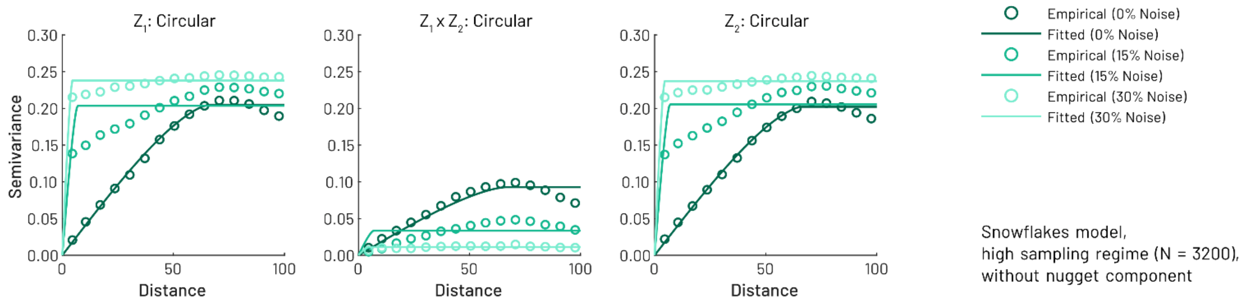

Figure S37. Synthetic snowflakes model: Empirical semivariograms and fitted circular kernel *without* a nugget component at three different noise levels for terms Z_1 and Z_2 and their cross-covariance in the high sampling regime (N = 3200) for a single run.

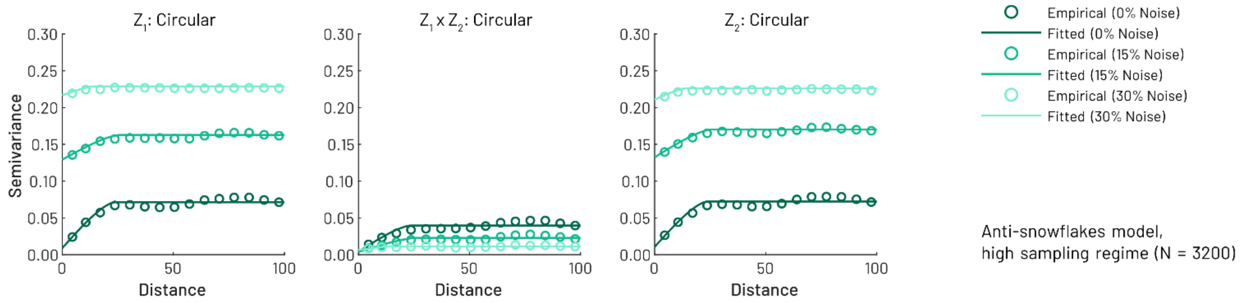

Figure S38. Synthetic anti-snowflakes model: Empirical semivariograms and fitted circular kernel *with* a nugget component at three different noise levels for terms Z_1 and Z_2 and their cross-covariance in the high sampling regime (N = 3200) for a single run.

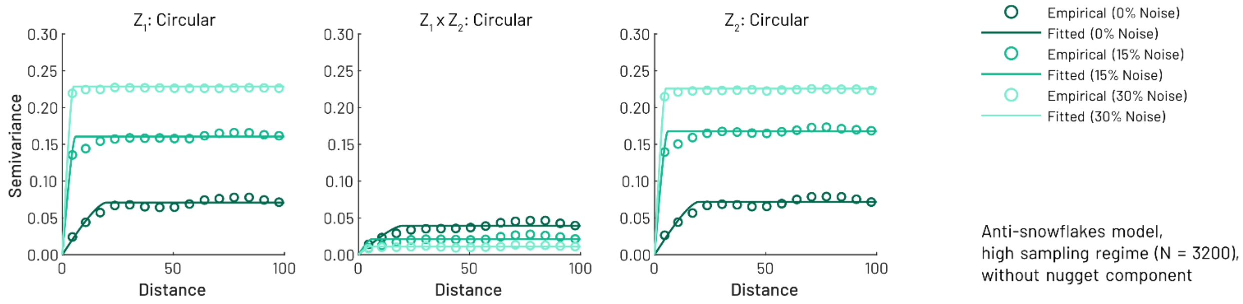

Figure S39. Synthetic anti-snowflakes model: Empirical semivariograms and fitted circular kernel *without* a nugget component at three different noise levels for terms Z_1 and Z_2 and their cross-covariance in the high sampling regime (N = 3200) for a single run.

S3.7.3 Exponential Kernel

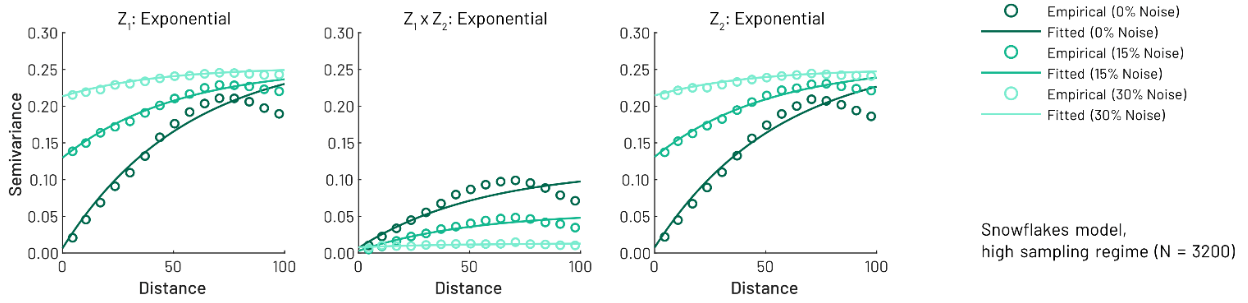

Figure S40. Synthetic snowflakes model: Empirical semivariograms and fitted exponential kernel *with* a nugget component at three different noise levels for terms Z_1 and Z_2 and their cross-covariance in the high sampling regime (N = 3200) for a single run.

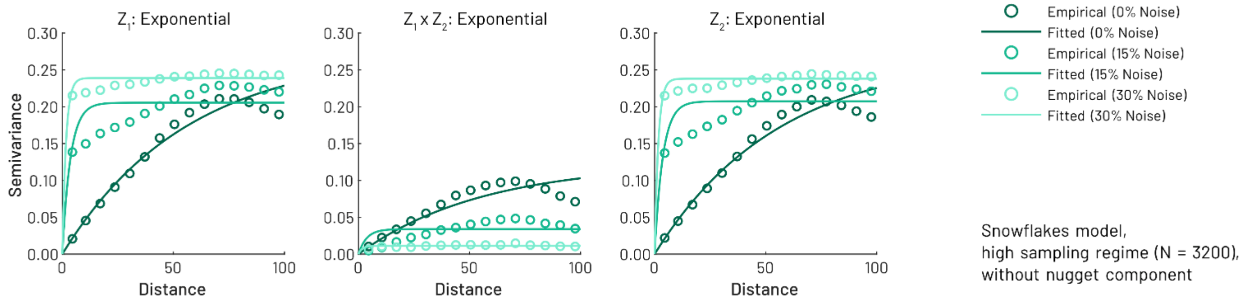

Figure S41. Synthetic snowflakes model: Empirical semivariograms and fitted exponential kernel *without* a nugget component at three different noise levels for terms Z_1 and Z_2 and their cross-covariance in the high sampling regime (N = 3200) for a single run.

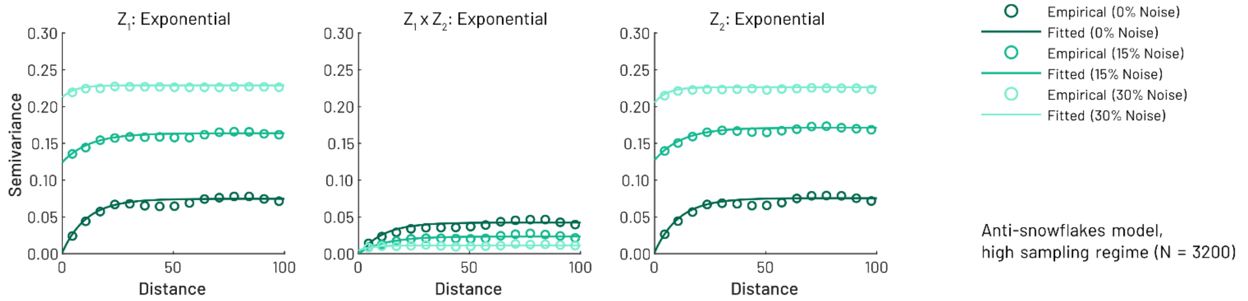

Figure S42. Synthetic anti-snowflakes model: Empirical semivariograms and fitted exponential kernel *with* a nugget component at three different noise levels for terms Z_1 and Z_2 and their cross-covariance in the high sampling regime (N = 3200) for a single run.

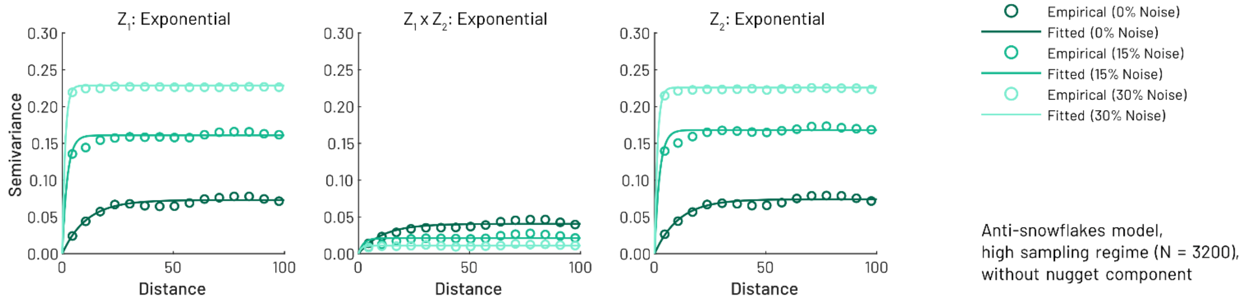

Figure S43. Synthetic anti-snowflakes model: Empirical semivariograms and fitted exponential kernel *without* a nugget component at three different noise levels for terms Z_1 and Z_2 and their cross-covariance in the high sampling regime (N = 3200) for a single run.

S3.7.4 Gaussian Kernel

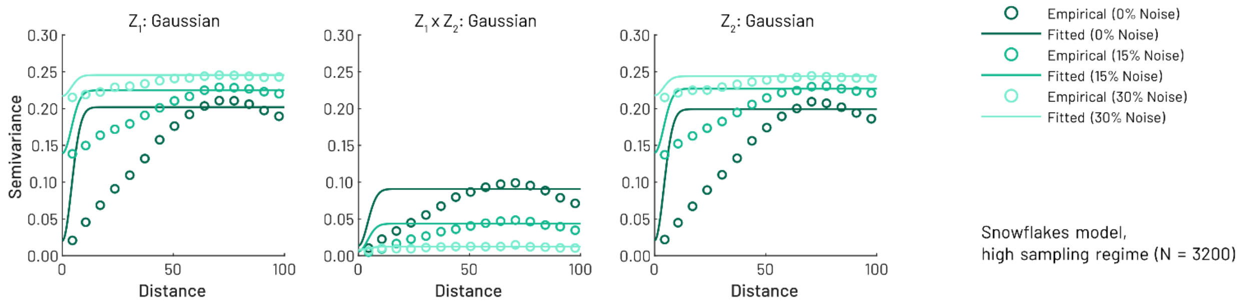

Figure S44. Synthetic snowflakes model: Empirical semivariograms and fitted Gaussian kernel *with* a nugget component at three different noise levels for terms Z_1 and Z_2 and their cross-covariance in the high sampling regime (N = 3200) for a single run.

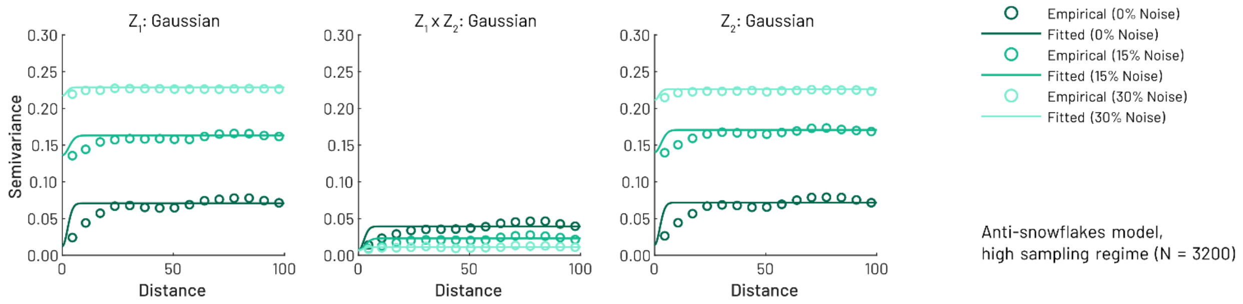

Figure S45. Synthetic anti-snowflakes model: Empirical semivariograms and fitted Gaussian kernel *with* a nugget component at three different noise levels for terms Z_1 and Z_2 and their cross-covariance in the high sampling regime (N = 3200) for a single run.

S3.7.5 Matérn Kernel

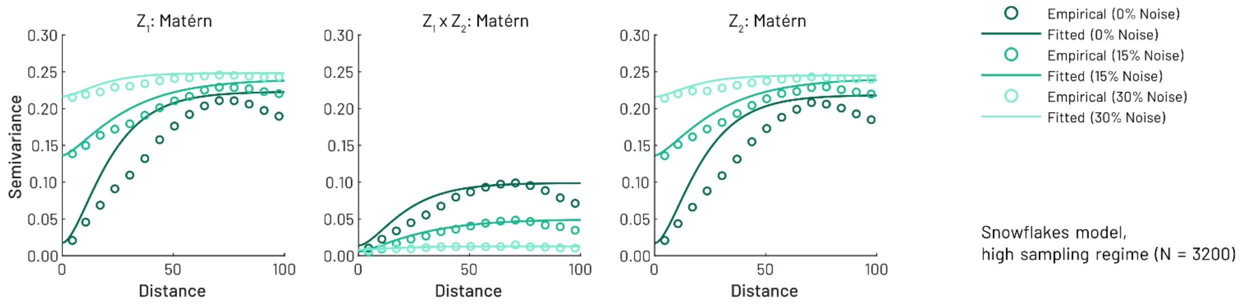

Figure S46. Synthetic snowflakes model: Empirical semivariograms and fitted Matérn kernel *with* a nugget component at three different noise levels for terms Z_1 and Z_2 and their cross-covariance in the high sampling regime (N = 3200) for a single run.

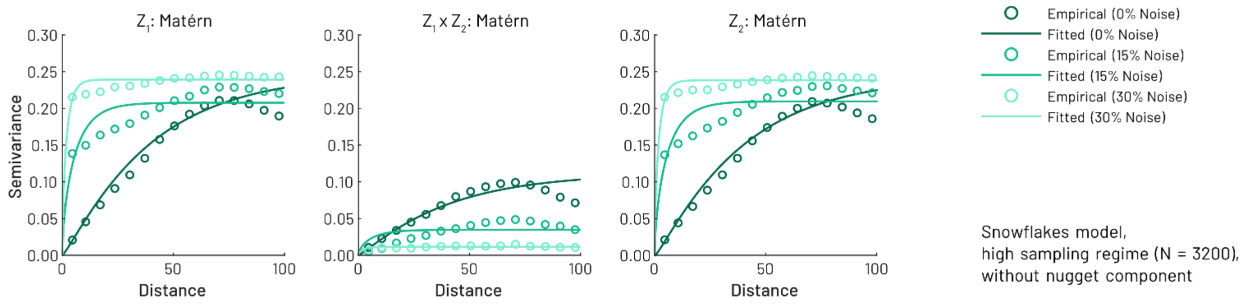

Figure S47. Synthetic snowflakes model: Empirical semivariograms and fitted Matérn kernel *without* a nugget component at three different noise levels for terms Z_1 and Z_2 and their cross-covariance in the high sampling regime (N = 3200) for a single run.

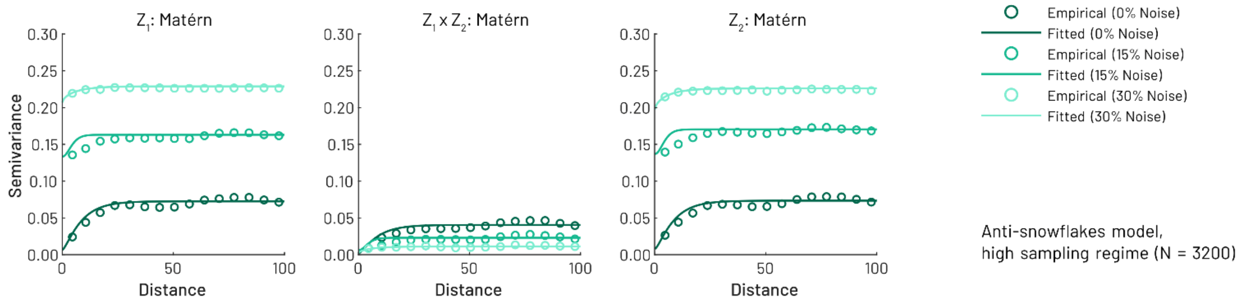

Figure S48. Synthetic anti-snowflakes model: Empirical semivariograms and fitted Matérn kernel *with* a nugget component at three different noise levels for terms Z_1 and Z_2 and their cross-covariance in the high sampling regime (N = 3200) for a single run.

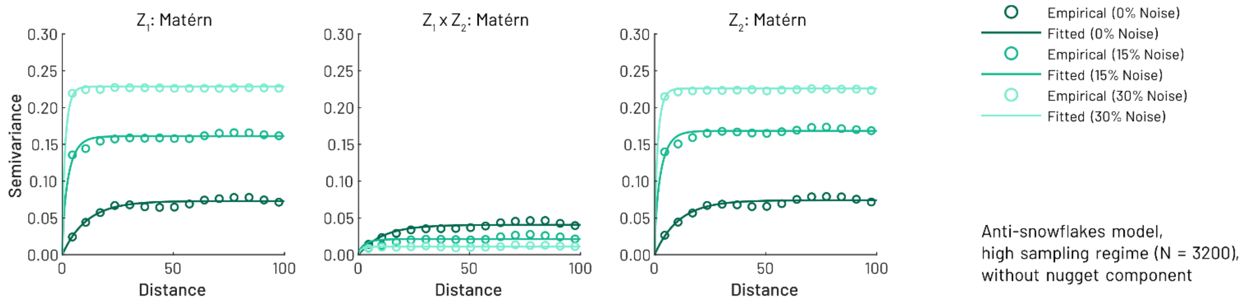

Figure S49. Synthetic anti-snowflakes model: Empirical semivariograms and fitted Matérn kernel *without* a nugget component at three different noise levels for terms Z_1 and Z_2 and their cross-covariance in the high sampling regime (N = 3200) for a single run.

S3.7.6 Spherical Kernel

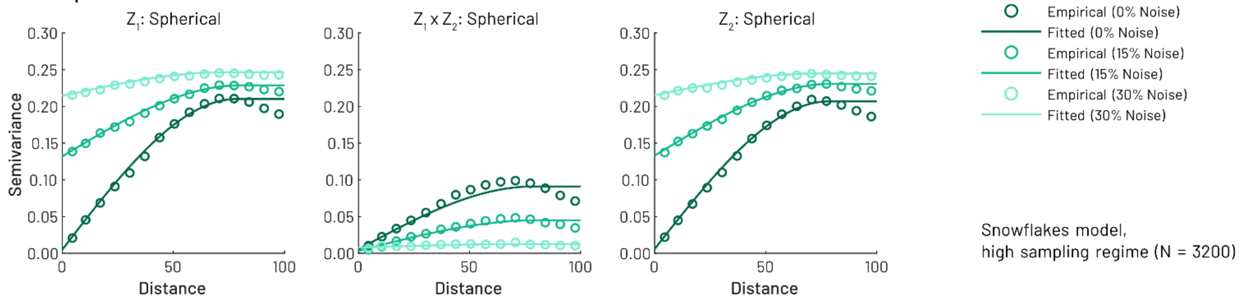

Figure S50. Synthetic snowflakes model: Empirical semivariograms and fitted spherical kernel *with* a nugget component at three different noise levels for terms Z_1 and Z_2 and their cross-covariance in the high sampling regime (N = 3200) for a single run.

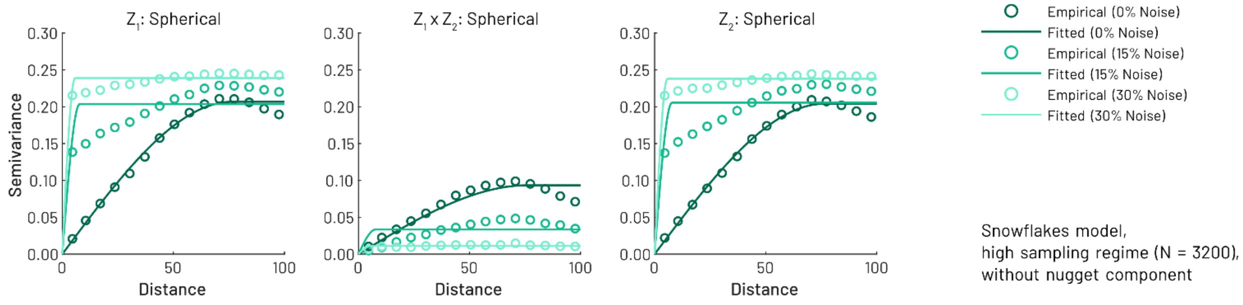

Figure S51. Synthetic snowflakes model: Empirical semivariograms and fitted spherical kernel *without* a nugget component at three different noise levels for terms Z_1 and Z_2 and their cross-covariance in the high sampling regime (N = 3200) for a single run.

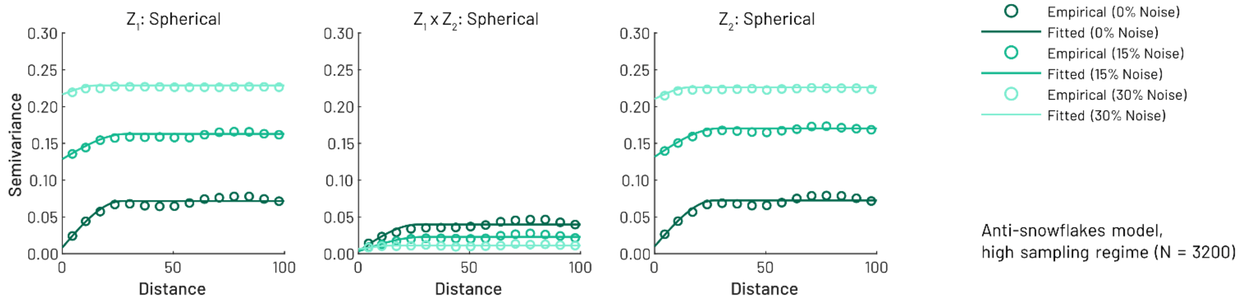

Figure S52. Synthetic anti-snowflakes model: Empirical semivariograms and fitted spherical kernel *with* a nugget component at three different noise levels for terms Z_1 and Z_2 and their cross-covariance in the high sampling regime (N = 3200) for a single run.

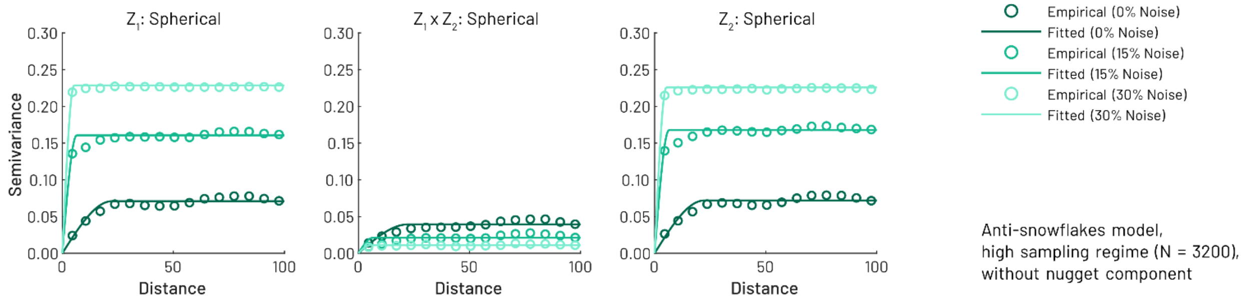

Figure S53. Synthetic anti-snowflakes model: Empirical semivariograms and fitted spherical kernel *without* a nugget component at three different noise levels for terms Z_1 and Z_2 and their cross-covariance in the high sampling regime (N = 3200) for a single run.

S3.7.7 Wave Kernel

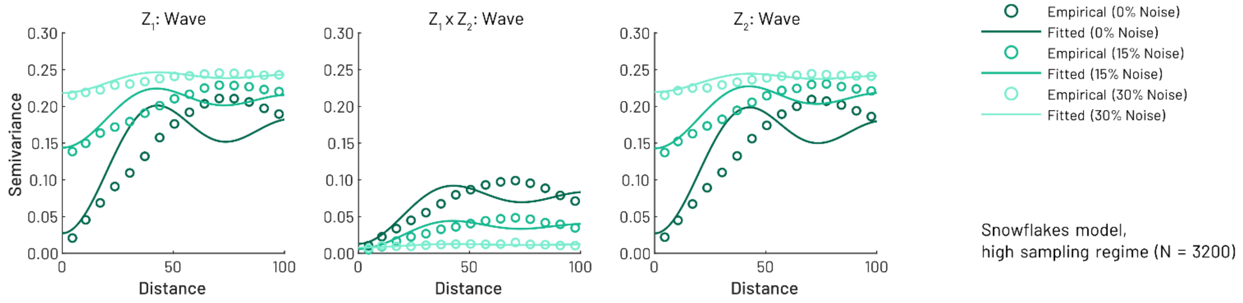

Figure S54. Synthetic snowflakes model: Empirical semivariograms and fitted wave kernel *with* a nugget component at three different noise levels for terms Z_1 and Z_2 and their cross-covariance in the high sampling regime (N = 3200) for a single run.

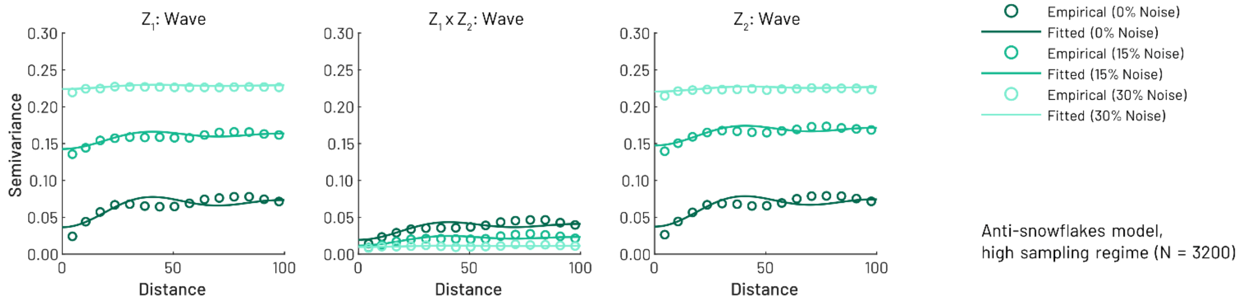

Figure S55. Synthetic anti-snowflakes model: Empirical semivariograms and fitted wave kernel *with* a nugget component at three different noise levels for terms Z_1 and Z_2 and their cross-covariance in the high sampling regime (N = 3200) for a single run.

ICD-9 codes

ICD-9 Group	Description	ICD-9 Codes in Group
250.0	Diabetes mellitus without mention of complication	250.00, 250.02
250.1	Diabetes with ketoacidosis	250.10, 250.12
250.2	Diabetes with hyperosmolarity	250.20, 250.22
250.3	Diabetes with other coma	250.30, 250.32
250.4	Diabetes with renal manifestations	250.40, 250.42
250.5	Diabetes with ophthalmic manifestations	250.50, 250.52
250.6	Diabetes with neurological manifestations	250.60, 250.62
250.7	Diabetes with peripheral circulatory disorders	250.70, 250.72
250.8	Diabetes with other specified manifestations	250.80, 250.82
250.9	Diabetes with unspecified complication	250.90, 250.92

Table S7. ICD-9 codes used in the extraction of the type II diabetes indicator variable.

S4 GeoSPM Software Overview

GeoSPM is implemented as a well-structured collection of MATLAB classes and packages in the "geospm" and "hdng" namespaces, preventing name-collisions with a user's existing MATLAB installation. It makes use of a separately provided SPM toolbox (synthetic_volumes_toolbox) to allow in-memory generation of SPM scan files, which we hope to integrate into SPM proper in the future. An overview of key classes and packages is shown in Figure S56. A potential user of GeoSPM invokes a single function – geospm.compute() – to initiate an analysis, passing a path to a working directory, a SpatialData object and a set of name-value options. All results will be stored as files in the given directory, including images of all regression coefficients and vector-based shape files demarking regions of significance for any applied thresholds. A SpatialData object can be constructed manually or obtained via loading a comma-separated value (CSV) file from disk via geospm.load_data(). In order to produce geo-referenced TIFF images, GeoSPM requires a SpatialData object to have an attached co-ordinate reference system. This can be specified when calling geospm.load_data() or manually, by creating a hdng.SpatialCRS object from an appropriate identifier. For example, 'EPSG:27700' is the identifier for the Ordnance Survey National Grid used by UK Biobank.

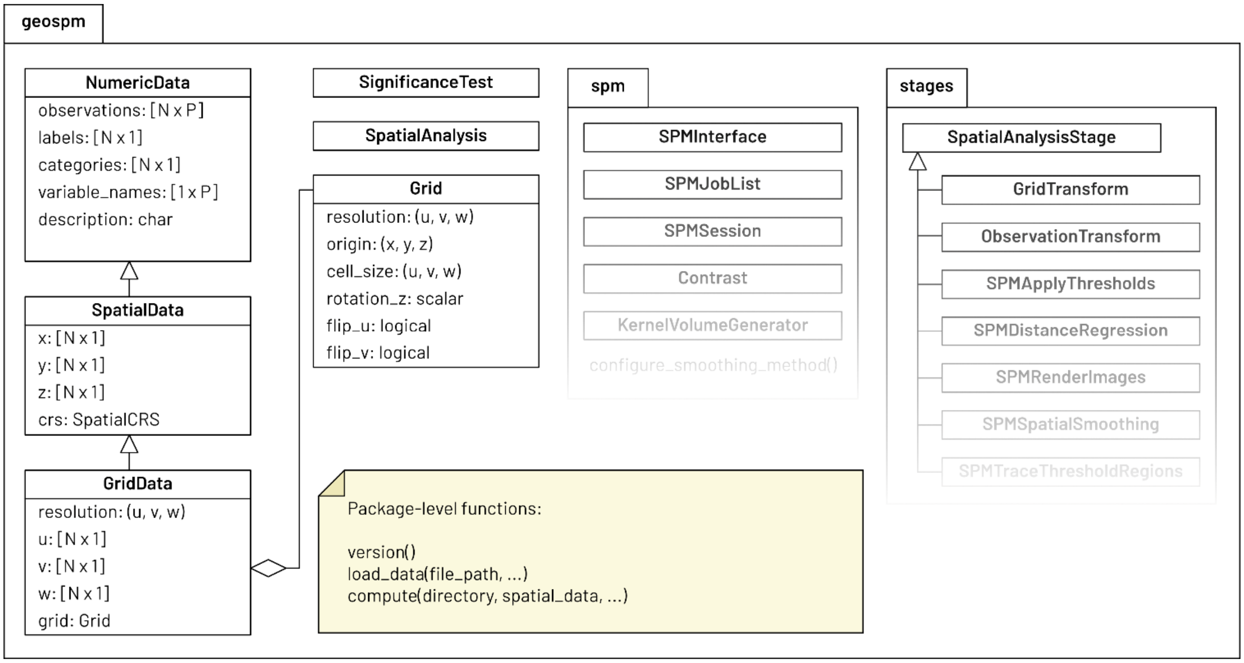

Figure S56. Class diagram of GeoSPM.

Internally, geospm.compute() uses a SpatialAnalysis object to define a pipeline comprising a number of successive processing stages, each concerned with a clearly de-lineated task, such as transforming continuous locations to discrete grid co-ordinates, rendering a Gaussian kernel of desired size at each location of the data, running SPM itself, colour-mapping output images, and extracting vector-based areas of significance for each threshold.

References

S1. Makalic, E. & Schmidt, D. F. (2016). High-dimensional Bayesian regularised regression with the BayesReg package. arXiv. DOI: 10.48550/arXiv.1611.06649.

S2. Friston, K. J., Holmes, A. P., Price, C. J., Büchel, C. & Worsley, K. J. (1999). Multisubject fMRI studies and conjunction analyses. Neuroimage *10*, 385–396. DOI: 10.1006/nimg.1999.0484.

S3. Goovaerts, P. (1997). Geostatistics for natural resources evaluation (Oxford University Press on Demand).

S4. Matérn, B. (1960). Spatial variation, Technical Report. Statens Skogsforsningsinstitut, Stockholm. https://pub.epsilon.slu.se/10033/1/medd_statens_skogsforskningsinst_049_05.pdf.

S5. Diggle, P. J. & Ribeiro, P. J. (2007). Model-based geostatistics (Springer). DOI: 10.1007/978-0-387-48536-2.

S6. Stein, M. L. (1999). Interpolation of spatial data: some theory for kriging (Springer Science & Business Media). DOI: 10.1007/978-1-4612-1494-6.
