# Supplementary material for: GeoSPM: Geostatistical parametric mapping for medicine
Source: Patterns (N Y). 2022 Dec 9;3(12):100656. doi: 10.1016/j.patter.2022.100656 (PMC9768692; doi:10.1016/j.patter.2022.100656)
Supplement: Document S2. Article plus supplemental information [file mmc2.pdf]

Patterns

Article

GeoSPM: Geostatistical parametric mapping for medicine

Highlights

- A framework for topological inference applicable to diverse clinical data is proposed
- Superior robustness to noise and under-sampling is observed compared with kriging
- Application to UK Biobank data is demonstrated

Authors

Holger Engleitner, Ashwani Jha, Marta Suarez Pinilla, ..., Karl Friston, Martin Rossor, Parashkev Nachev

Correspondence

h.engleitner@ucl.ac.uk (H.E.),
p.nachev@ucl.ac.uk (P.N.)

In brief

We present GeoSPM, an approach to the spatial analysis of diverse clinical data that extends a framework for topological inference, well established in neuroimaging, based on differential geometry and random field theory. We evaluate GeoSPM with extensive synthetic simulations, and apply it to large-scale data from UK Biobank. Our approach is readily interpretable, easy to implement, enables flexible modeling of complex spatial relations, exhibits robustness to noise and under-sampling, offers principled criteria of statistical significance, and is scalable to large datasets.

Engleitner et al., 2022, Patterns *3*, 100656
December 9, 2022 © 2022 The Author(s).
https://doi.org/10.1016/j.patter.2022.100656

CellPress

Patterns

CellPress
OPEN ACCESS

Article

GeoSPM: Geostatistical parametric mapping for medicine

Holger Engleitner,[1,*] Ashwani Jha,[1] Marta Suarez Pinilla,[1] Amy Nelson,[1] Daniel Herron,[2] Geraint Rees,[1] Karl Friston,[1] Martin Rossor,[1] and Parashkev Nachev[1,3,*]

[1]UCL Queen Square Institute of Neurology, University College London, London WC1N 3BG, UK
[2]Research & Development, NIHR University College London Hospitals Biomedical Research Centre, London W1T 7DN, UK
[3]Lead contact
*Correspondence: h.engleitner@ucl.ac.uk (H.E.), p.nachev@ucl.ac.uk (P.N.)
https://doi.org/10.1016/j.patter.2022.100656

THE BIGGER PICTURE Many aspects of health and disease are distributed in space, requiring models of topological organization. The complexity of the task, however, makes spatial analysis comparatively rare in medicine. Here, we introduce GeoSPM, a platform for topological inference from clinical data based on a mature mathematical framework—statistical parametric mapping—validated by decades of use in neuroimaging. We provide comprehensive synthetic evaluation of the approach, and illustrate its application on large-scale data from UK Biobank. The interpretability, flexibility, scalability, ease of implementation, robustness to noise and under-sampling, computational efficiency, and provision of principled criteria of statistical significance, provided by our open-source platform should catalyze wider use of spatial analysis across medicine.

1 2 3 4 5 **Development/Pre-production:** Data science output has been rolled out/validated across multiple domains/problems

SUMMARY

The characteristics and determinants of health and disease are often organized in space, reflecting our spatially extended nature. Understanding the influence of such factors requires models capable of capturing spatial relations. Drawing on statistical parametric mapping, a framework for topological inference well established in the realm of neuroimaging, we propose and validate an approach to the spatial analysis of diverse clinical data—GeoSPM—based on differential geometry and random field theory. We evaluate GeoSPM across an extensive array of synthetic simulations encompassing diverse spatial relationships, sampling, and corruption by noise, and demonstrate its application on large-scale data from UK Biobank. GeoSPM is readily interpretable, can be implemented with ease by non-specialists, enables flexible modeling of complex spatial relations, exhibits robustness to noise and under-sampling, offers principled criteria of statistical significance, and is through computational efficiency readily scalable to large datasets. We provide a complete, open-source software implementation.

INTRODUCTION

Human beings vary along a rich multiplicity of social and biological dimensions, whose complex interactions across health and disease present a challenge for medical science and systems biology in general. The combination of large-scale data with machine learning promises to cast brighter light on this complexity than conventional inferential techniques, illuminating distributed, long-range dependencies hitherto obscured. Our interventions are increasingly grounded in an understanding of the factors that shape disease trajectories and determine individual responses to treatment.

One comparatively neglected dimension is the literal dimension of space: each of us inhabits a particular location that may reflect or modify our individual biological characteristics and the influence of (and on) other spatially distributed variables. Spatial factors may be static or vary over time, arising at multiple scales, ranging from the domestic to the inter-continental. Their reference frames may be set by internal communities, by external geographies, or by a complex blend

This is an open access article under the CC BY license (http://creativecommons.org/licenses/by/4.0/).

of the two. Their spatial organization may be linear or consistently distorted by individual or environmental movement within these frames of reference. Spatial factors may disclose or alter characteristics of biology directly, or render them more or less clinically accessible or actionable. Space arises not only in epidemiology, environmental medicine, healthcare policy, and public health, but in the fundamental organization of biology itself.

Yet outside a few specialist areas spatial analysis is comparatively rare in medicine. An indicative survey of published paper titles and abstracts in Microsoft Academic Graph, spanning 30 years of medical research, reveals only 1,897 journal papers at the intersection of geospatial analysis and medicine, with an annual citation distribution for those cited more than once nonetheless substantially higher than a matched biomedical sample (mean 2.75 versus 2.13, Mann-Whitney U test, $p < 0.001$, Figure S1, see supplemental note). The comparative scarcity is arguably in part explained by the difficulty of the task. The spatial factors arising in a medical context are often entangled, their sampling is sparse and frequently corrupted by noise, and the underlying signals tend to be weak. But spatial analysis is hard even where the data regime is benign, for the problem is essentially multidimensional and is rarely, if ever, open to analytic solutions.

The fundamental challenge is reflected in the wide array of techniques in current use. A survey of 397 papers published since January 1, 2017, in the joint domains of health and spatial modeling identifies local indicators of spatial association,[1] spatial scan statistics,[2] inverse distance weighting,[3] kernel density estimation,[4,5] spatial regression in terms of spatial lag and spatial error models,[6] geographically weighted regression (GWR),[7] land-use regression,[8] kriging,[9,10] generalized linear mixed models,[11] generalized (geo-)additive models,[12,13] hierarchical Bayesian spatial analysis,[14,15] and model-based geostatistics,[16,17] among others.

This methodological diversity reflects differing demands on the spatial aspects of the model and the breadth of specific questions that arise in a spatial setting. With the question may vary the modeling objective, and the theoretical assumptions that underpin it. Common objectives include spatial prediction, the analysis and regression of spatially varying or spatially confounded associations, and the investigation of spatial point patterns. Arguably the most general and taxing research questions involve inference—whether explicit or not—to a topological organization, for example, identifying the location and extent of a spatially organized signal buried in noise. Such questions typically—if not always—require methods that treat space as a continuity, produce spatially continuous estimates, and provide principled measures of spatial uncertainty. Dominant in this category are methods that adopt a nonlinear multivariate approach, taking advantage of the flexibility and expressivity it offers. Although potentially powerful, they require joint expertise in the method and the domain of its application, depend on prior specification of model parameters, and tend to demand substantial computational resource even for data of moderate scale. Furthermore, in the generalized linear framework, space commonly enters the model as a latent random effect—usually derived from a suitable Gaussian process. This approach adjusts for spatially correlated variance within an otherwise non-spatial framework, with the fixed effects remaining constant across the spatial field.[18]

These obstacles motivate the pursuit of alternatives outside the multivariate paradigm for the task of topological inference. The direct counterpoint is a mass-univariate approach, where a complex multivariate model is replaced by a spatially indexed ensemble of simpler models. GWR modifies the predictors in a regression model through a spatially localized weight matrix, so that a variation of the model is estimated at each location and the resulting estimates exhibit spatial smoothness. Although GWR estimates can be derived from a prespecified grid, in practice only sampled locations or grids of modest size tend to be evaluated owing to the difficulty of correcting for multiple comparisons in a topologically informed manner.[19] Spatial inference with GWR is commonly limited to regression coefficient or coefficient of determination maps that simply indicate the local goodness of fit,[20] without employing formal tests of significance.[21,22] Finally, these are regression models relating a response to a set of spatially organized predictors, not models of the spatial variation of a set of variables within a topological framework of uncertainty: our primary concern.

Here, we propose, implement, and validate an approach to the spatial analysis of diverse clinical or public health data that draw upon differential geometry and random field theory, with the topological objective of identifying connected neighborhoods and peaks of spatial significance. In particular, we leverage the procedures used in statistical parametric mapping (SPM): a framework for making topological inferences about spatially structured effects, with well-behaved spatial dependencies.[23] This approach has been established for decades in the realm of (structural and functional) volumetric neuroimaging.

The core idea is to transform sparse spatial signals into a form suited to mass-univariate statistical testing on a chosen point grid: for example, testing that the spatial or regional expression of a particular variable is greater than would be expected under the null hypothesis of no regional effect. The probability of observing topological features in the observed map, such as peaks or clusters (i.e., level sets above some threshold), can then be evaluated with classical inference based on random field theory, and used to ascribe a p value to spatially organized effects. This principled approach radically simplifies one important domain of spatial analysis, rendering it potentially more sensitive and robust to noise, and places it on a formal inferential footing, yielding a general-purpose geostatistical tool readily deployable across a multitude of medical fields where the modeling objective requires inference to the topological organization of a set of signals of interest. For example, we may use the approach to infer the location and extent of regional expression of spatially organized variables—taken alone or in conjunction—such as disease prevalence in a community, while accounting for multiple potentially interacting confounding factors, and without relying on any *a priori* parcellation of the space.

In what follows, we (1) offer a detailed rationale for our approach; (2) proceed to evaluate it across an extensive array of synthetic simulations where the nature of the spatial relationships, sampling, and corruption by noise are prespecified; and (3) demonstrate its application on large-scale data from UK Biobank (https://www.ukbiobank.ac.uk/).[24] The numerical analyses serve to establish face validity; the empirical analysis to

CellPress
OPEN ACCESS

demonstrate predictive validity. We provide a complete, open-source software implementation of our framework (https://github.com/high-dimensional/geospm), released as an extension to SPM; namely, geospatial SPM or "GeoSPM." Supplemental note S4 and Figure S56 provide an overview of GeoSPM's class structure as implemented in MATLAB.

EXPERIMENTAL PROCEDURES

Resource availability

Lead contact

Further information and requests for resources should be directed to and will be fulfilled by the lead contact, Holger Engleitner (h.engleitner@ucl.ac.uk).

Materials availability

This study did not generate new unique reagents and did not use any additional materials aside from the data and code cited below.

Data and code availability

The data analyzed in this study are available on application to UK Biobank (https://www.ukbiobank.ac.uk). The open-source software implementation of GeoSPM presented in this study is available on GitHub: https://github.com/high-dimensional/geospm (https://doi.org/10.5281/zenodo.7258971).

Overview

Our approach builds on the well-established regression analysis framework implemented in SPM12 (http://www.fil.ion.ucl.ac.uk/spm/), the most widely used platform for spatial inference in brain imaging. Within this framework, a set of explanatory variables is associated with a multivariate, spatially structured response, whose components represent measurements taken at regular locations in a spatial domain. The association between explanatory variables and response is estimated at each location separately, using the same general linear model (GLM). This yields a collection of univariate multiple regression models that share the same model architecture and design matrix but differ in the response variable and the estimated parameter values. Crucially, random fluctuation, or variations in the response variable that are not explained by the GLM, are treated as realizations of a random (spatial) field with certain contiguity or smoothness properties. This is *mass-univariate* inference from a spatial perspective.

A distinguishing feature of SPM is the manner of correcting for multiple comparisons when testing mass-univariate model parameters (i.e., regression coefficients) for significance. The large number of tests, performed simultaneously, gives rise to a proportionally large number of false positives by chance alone. Conversely, the strong spatial correlations among the components of the response violate assumptions of mutual independence, and render simple Bonferroni correction inappropriately strict. SPM applies a more suitable correction by modeling the residuals as a random Gaussian field, so that p values are meaningful in terms of identifying significant peaks and clusters in a discretized spatial domain. Heuristically, topological inference of this kind automatically accounts for spatial dependencies; in the sense that smooth random fluctuations will produce a smaller number of maxima than rough random fields with less spatial dependence (even though the total area above some threshold could be the same). It can be shown that the smoothness of the residual fields is a suitable approximation to the smoothness of a t statistic map derived from the model, which in turn reflects the spatial dependence of the covariates.[25,26]

The kind of data we are concerned with comprise variables of interest observed at locations in a continuous spatial domain D. D is usually a subset of $\mathbb{R}^2$ representing coordinates of a geographic space. More precisely, every element in a spatially referenced dataset associates a vector $\boldsymbol{y}_i$ of P variable observations $(y_{i1}, \dots, y_{iP})^\top \in \mathbb{R}^P$ with a location $\boldsymbol{x}_i \in D$

$$(\boldsymbol{y}_i, \boldsymbol{x}_i) : i = 1, \dots, N.$$

SPM typically requires data sampled at regular locations across a grid, spanning the spatial domain. However, we wish to analyze data that are irregularly and sometimes sparsely sampled. This can be resolved by distributing each data point locally—over regular grid locations—using a spatial Gaussian kernel of suitable and fixed variance.

From a data-centric point of view, we can interpret this spatial transformation as estimating the contribution of an individual observation to regular sample points, where the contribution has a maximum value at the observation location and then diminishes with increasing distance. In this way, the dependent variable in the univariate regression at any location of space is essentially a weighting of individual observations according to their proximity to that location: the higher the local response, the closer the observation. We can do this with impunity because we are interested in the explainable differences in these contributions at prespecified (grid point) locations. These explainable differences are assessed with normalized effect sizes (i.e., classical statistics), which are not affected by the total contribution or variance.[23,27]

The chosen variance of the Gaussian kernel is a parameter—hereafter called the *smoothing* parameter—deliberately left open to the analyst to specify the appropriate degree of spatial coarse graining (i.e., spatial smoothness of the data features in question). Since SPM naturally handles volumetric data, we are free to use the third dimension to model multiple smoothing values on a continuous positive scale, rendering them as different spatial "scales" or "features" of a response variable.[28] Here, two coordinates represent the location in space (i.e., location space), and the third coordinate tracks spatial spread (i.e., scale space), allowing the regression analysis to operate at different scales simultaneously. It is appropriate to permit inference under varied assumptions of uncertainty, allowing the analyst to draw conclusions from the similarities and differences obtained across the range of plausible spatial scales. The analyst is also free to implement mechanisms that select an optimal parameter under some criterion: here we suggest one pragmatic method of doing this. Note that this scale-space implementation of topological inference automatically accounts for dependencies in moving from one scale to another and enables topological inference in terms of maxima or clusters in both location and scale space (i.e., a particular effect can be declared significant at this location and this spatial scale). For simplicity, we will focus on topological inference at a given spatial scale.

Downstream of the above spatial transformation of data features, the statistical approach is formally identical to a standard SPM analysis. The output comprises a series of volumes representing regression coefficients, statistical contrasts derived from these model parameters, the statistical parametric maps—of classical statistics based on these contrasts—and, finally, thresholded binary maps that indicate whether the voxels in the corresponding statistical map are significant at the chosen (suitably corrected) p value.

Synthetic data and generative models

The statistical validity of the proposed approach is underwritten by the assumptions on which SPM rests. Nonetheless, it is helpful to examine its construct validity, in comparison with alternative methods (e.g., kriging), and face validity, in terms of its ability to recover known effects in different situations. Such validation is best performed with a known (spatial) ground truth, under manipulations of sampling and noise traversing the plausible space of possibility as far as is practicable. Note, however, that no aspect of the modeling approach—as opposed to its validation—may be allowed to rely on a ground truth, for in topological inference—as opposed to prediction—no ground truth is generally available. We cannot, for example, use a ground truth to tune a hyperparameter without excluding precisely the inferential context we are interested in.

For maximum flexibility and control over the evaluation process, here we use synthetic data drawn from a generative model with a spatially varying distribution of one or two joint binary variables. The spatial variability of the distribution is determined by the locale and extent of shapes with a fractal boundary. Fractals characteristically exhibit detail across an infinite range of spatial scales, which makes them ideal candidates for a spatially structured ground truth with sensitivity to the widest possible range of spatial scales. The use of binary variables to generate two distinct signal levels for the response allows us to focus on data that are generated in a spatially structured way; namely, in a regionally specific fashion under various levels of noise or stochasticity. A full description of the process is provided in supplemental note S2.

Demonstration with UK Biobank data

To demonstrate the application of GeoSPM to real data, we chose to explore the potential association between a common disease—type 2

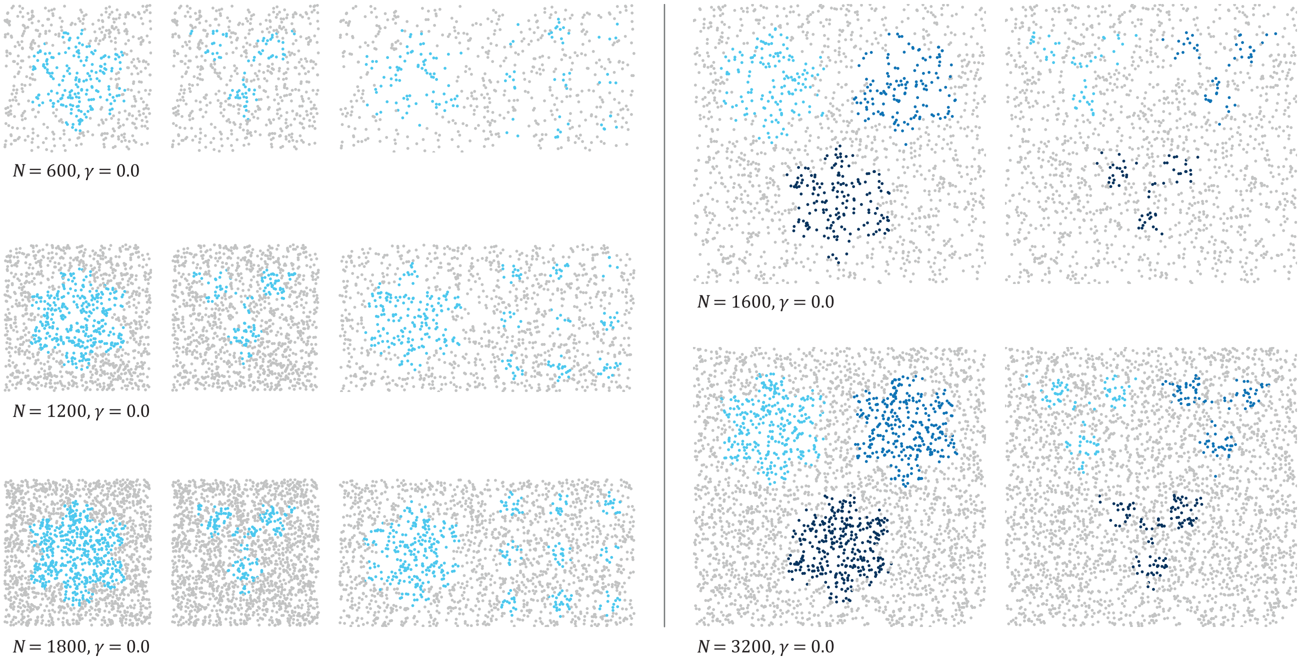

Figure 1. Sampling levels (noise-free, $\gamma = 0.0$) for the univariate models on the left ($N = 600, 1200, 1800$), and for the bivariate models on the right ($N = 1600, 3200$)

diabetes—and a small number of demographic variables in UK Biobank drawn from the area of Greater Birmingham. It should be stressed that the sole purpose of this analysis was to illustrate the application of the method, not to make inferences about the data itself, which would require more detailed investigation than our foundational focus here permits. The objective instead is to illustrate how spatial variation of a variable of interest may be examined, with specific attention to two important contexts: where the effect of the variable must be isolated from a set of known potential confounders, and where the joint effects of two or more variables are of interest. A detailed description of the variable selection and preprocessing is given in supplemental note S2.2.

Numerical experiments

Kriging

We evaluate GeoSPM in comparison with the well-established multivariate geostatistical method of kriging, described in detail in supplemental note S2.3. All kriging computations were done in R using the gstat package,[29] which is available at https://cran.r-project.org/web/packages/gstat/index.html. For each variable of interest kriging produced an image of the predicted mean and an image of the corresponding prediction variance, which is derived solely from the arrangement of positions in the data, i.e., the prediction variance does not depend on the values of the observations, only on their locations.

Synthetic experiments: Noise parameterization

The numerical face validation experiments are based on three univariate models (snowflake, anti-snowflake, snowflake field) and two bivariate models (snowflake, anti-snowflake) as depicted in Figures S2 and S3. For all models, we ran experiments at different sampling levels, $N_{univariate} \in \{600, 1200, 1800\}$ and $N_{bivariate} \in \{1600, 3200\}$, and increased the noise parameter γ from 0.0 to 0.35 in 0.01 increments (Figure 1). For each triplet (model, N, γ), 10 independent datasets were randomly generated.

Each generated dataset was processed by GeoSPM as well as gstat. For GeoSPM, the spread of the spatial response at locations $\boldsymbol{x}_i$, i.e., the spatial distribution of the response following smoothing, was modeled at increasing smoothing parameter values ($\ell = 10$) using the 95% iso-density diameters of the bivariate normal distribution, $\boldsymbol{s} = (10, 15, 20, 25, 30, 35, 40, 45, 50, 55, 60)^{\mathsf{T}}$. This measure of spread is the diameter of a circle that contains 95% of the probability mass of a two-dimensional Gaussian distribution at its center. The largest value of the smoothing parameter, 60, was chosen to be half the height of the grid for the univariate models. The regression coefficients estimated by GeoSPM were tested using a one-tailed t test at $p < 0.05$ FWE (voxel-level, family-wise correction), producing a stack of ℓ binary maps of significant areas for every variable of interest. To derive corresponding maps—one per variable—for kriging, we compared a standardized form of the kriging prediction $\hat{y}_{std}(j,k)$ with the critical value of the upper tail probability $p < 0.05$ of the normal distribution. We standardized $\hat{y}(j,k)$ at each grid cell (j,k) using its estimated (positional) variance $\hat{\sigma}(j,k)$ and assuming a null mean of 0.5 to produce $\hat{y}_{std}(j,k)$:

$$\hat{y}_{std}(j,k) = \frac{\hat{y}(j,k) - \mu_{null}}{\hat{\sigma}(j,k)}, where (j,k) \in D', \mu_{null} = 0.5.$$

For a fair comparison with kriging, one of the ℓ smoothing values and its associated maps produced in a run of GeoSPM had to be chosen. We based this choice on maximizing the spatial coverage by the significant areas at each spatial scale (see Figure 2), while minimizing the spatial overlap between them. A spatial condition in the context of the observed variables $\boldsymbol{Y} \in \mathbb{R}^P$ in our models is obtained by applying a threshold of 0.5 to all observations, recording as 1 if an observed variable value exceeds the threshold or 0 if it does not. Each observation of a univariate model can thus be assigned one of two spatial conditions, or one of four conditions in the case of a bivariate model. We obtain the significant areas for each spatial condition by running a separate analysis in GeoSPM on a set of data that represents the spatial condition of each observation as a one-hot encoding, i.e., with each category represented as a set of binary dummy variables.

This approach enabled us to derive a score for each of the ℓ smoothing values, which simply comprised the total number of significant grid cells that appeared for exactly one of the spatial conditions, thereby ignoring any overlap. The smoothing value with the highest score was selected, together with the binary maps of significant areas computed from it. Ties were broken by choosing the smallest scale.

The binary maps for each variable were assessed relative to their respective target maps, which were derived by thresholding the corresponding marginal distribution of the model, adding grid cells with a probability greater than 0.5 to the target. We applied a number of representative image

CellPress
OPEN ACCESS

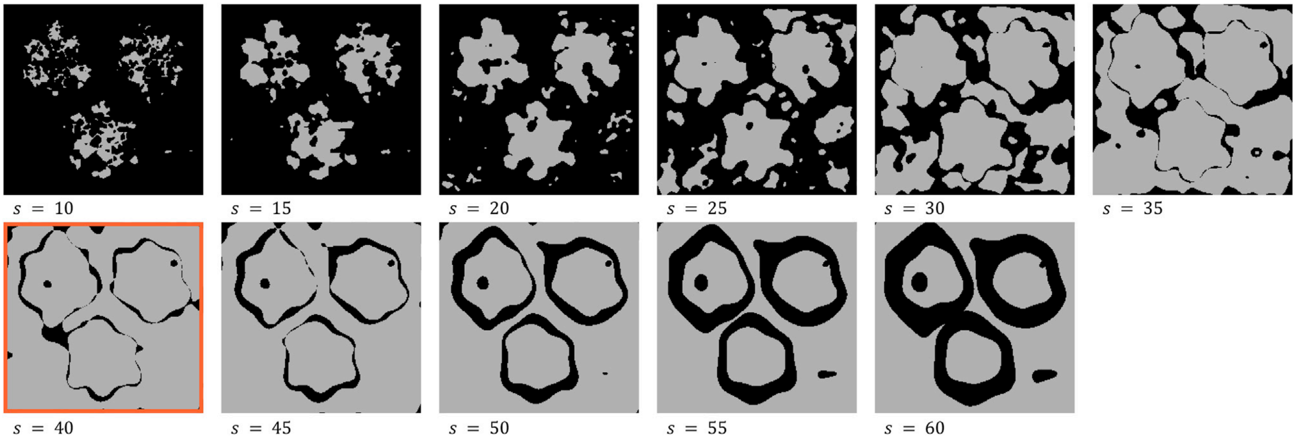

Figure 2. Example of a coverage computation for an instance of the bivariate snowflake model with noise $\gamma = 0.1$ and $N = 1600$

For each value s of the smoothing parameter, the combined significant areas for all four spatial conditions $(Z_1, Z_2) \in \{(0, 0), (1, 0), (0, 1), (1, 1)\}$ as determined by a separate run of GeoSPM are shaded in light gray. The maximum number of significant grid cells is obtained for $s = 40$, highlighted in red.

segmentation metrics to each pair of maps, computing a mean score over the 10 repetitions of each unique triplet (model, N, γ) and variable. The following metrics were used[30,31]: Jaccard index, Dice score, Matthew's correlation coefficient, symmetric uncertainty and the modified Hausdorff distance (as a fraction of the length of the model diagonal). The mean score and deviation for each metric and computation method were aggregated into the plots reported below.

Numerical experiments: Interaction parameterization

These experiments used the snowflake interaction model above and comprise observations of its variables, augmented by an interaction term. The

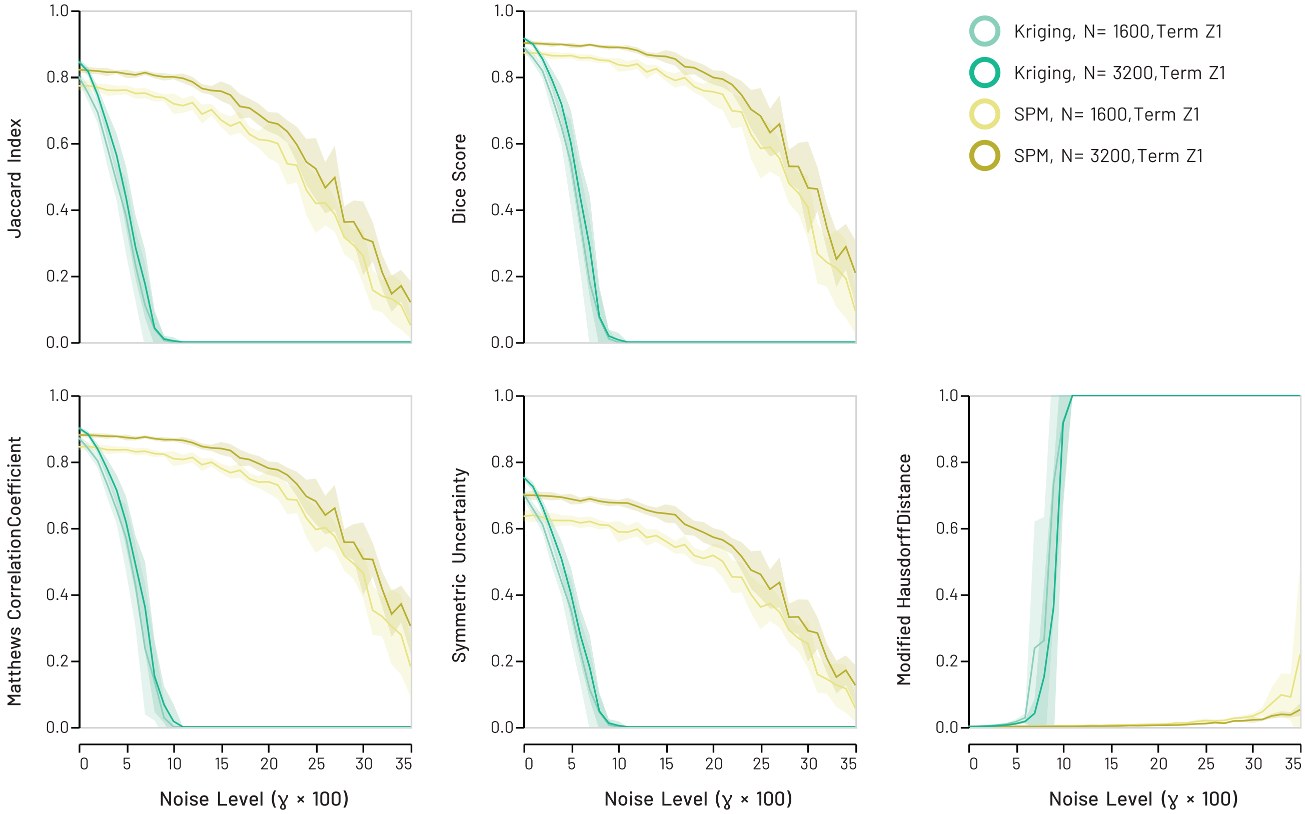

Figure 3. Synthetic snowflake models: recovery scores for GeoSPM and kriging of model term Z_1 in the low (N = 1600) and high (N = 3200) sampling regimes

Lines denote the mean score across 10 random model realizations, shaded areas its SD to either side of the mean. Areas of overlapping performance are identified by additive shading. GeoSPM degrades more slowly and gracefully as noise increases compared with kriging. Comparable results for model term Z_2 are shown in Figure S10.

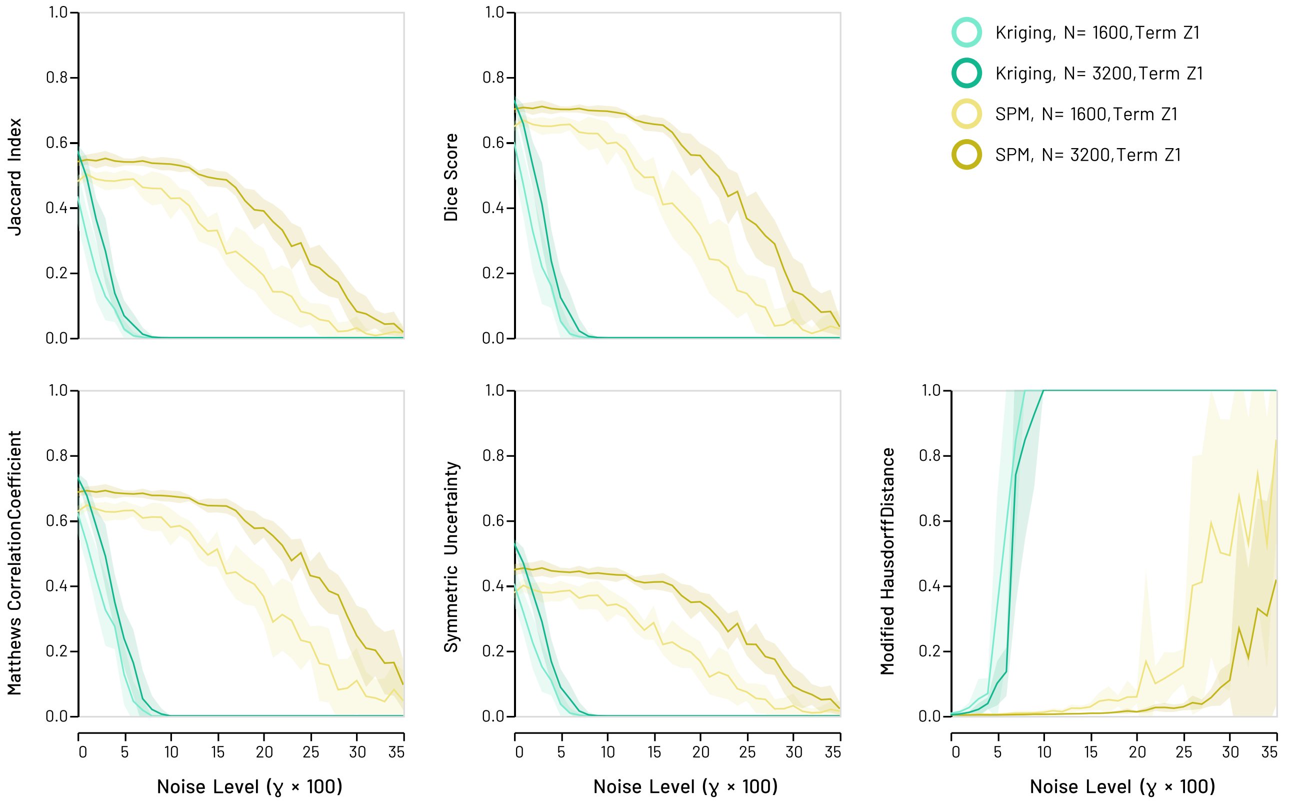

Figure 4. Synthetic anti-snowflake models: recovery scores for GeoSPM and kriging of model term Z_1 in the low (*N* = 1600) and high (*N* = 3200) sampling regime

Lines denote the mean score across 10 random model realizations, shaded areas its SD to either side of the mean. Areas of overlapping performance are identified by additive shading. As is the case with the snowflake models, GeoSPM degrades more slowly and gracefully as noise increases compared with kriging. Comparable results for model term Z_2 are shown in Figure S11.

interaction term is formed in the usual manner, by multiplying the observed values for both variables, yielding augmented observations: $\mathbf{y}' = (y_1, y_2, y_1 \cdot y_2)^T$. The regional arrangement of the model is the same as the one employed for the bivariate snowflake model shown on the left of Figure S2. A single sampling level $N_{interaction} = 15000$ was used and the interaction parameter c_3 was increased from 0.25 to 0.5 in steps of 0.05. For each level of c_3, $R = 10$ independent datasets were randomly generated. We set a single value for the smoothing parameter $s = 60$, which was the highest value for the noise experiments. As before, a one-tailed t test at $p < 0.05$ FWE (voxel-level family-wise correction) determined areas of significance and the same set of image segmentation metrics was computed for the binary maps.

UK Biobank experiments

Results for the UK Biobank data were obtained by a single invocation of GeoSPM for each of the four models listed in Table S6. We choose a smoothing value of 7 km, specified as the diameter of a patch enclosing 95% of the density the bivariate normal distribution with equal variances. This represents 20% of the width and height of our Birmingham analysis area, and seemed appropriate for identifying local variation sensitive to the plausible spatial scale of distinct geographically defined communities. This time, a two-tailed t test at $p < 0.05$ FWE (voxel-level family-wise correction) was used for thresholding the statistic maps. Analysis is restricted to areas where the combined smoothing density of all observations is at least 10 times the kernel peak value.

Ethical approval

UK Biobank has approval from the North West Multi-centre Research Ethics Committee as a Research Tissue Bank (RTB) approval. This approval means that researchers do not require separate ethical clearance and can operate under the RTB approval.

RESULTS

Our numerical experiments with a known generative model enabled us to measure performance against a known ground truth under circumstances varying in density of sampling and contamination with noise, enclosing the range likely to obtain in real-world scenarios. It also permits robust evaluation of graded interaction effects. In total, 2,160 independent simulations with synthetic data were performed for the univariate models, 1,440 for the bivariate models and 60 for the interaction model. Summarizing scores within the three sets of simulations, we derive performance curves for GeoSPM and kriging solutions in each case. We then proceed to illustrate the application of GeoSPM to real world data from UK Biobank.

Synthetic models

Displayed in the following figures are sets of independent simulations comparing the performance of GeoSPM (in yellow) versus kriging (in green) as a function of contaminating noise, measured by five different indices of retrieval fidelity, using the snowflake (Figure 3) or anti-snowflake (Figure 4) bivariate ground truths, and low or high data sampling regimes (similar results for the univariate ground truths are reported in Figures S7–S9 in

CellPress
OPEN ACCESS

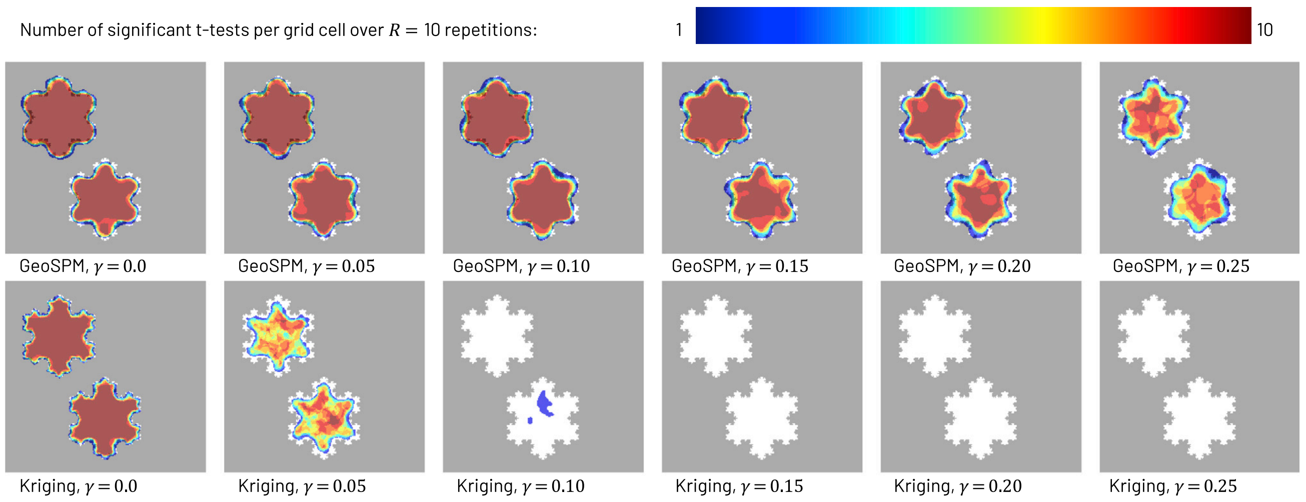

Figure 5. Recoveries of variable Z_1 in the synthetic bivariate snowflake model across $R = 10$ repetitions for GeoSPM in the top row and kriging with a Matérn kernel and nugget component in the bottom row, both in the high sampling regime ($N = 3200$)

Grid cells that lie in the target region are shown in white, those outside in gray. The number of significant tests out of 10 repetitions is superimposed in color for each grid cell: dark blue indicates at least one significant test and dark red indicates the maximum number of 10, while cells with no significant test did not receive any color. Kriging only produces recoveries up to a γ value of 0.10, whereas GeoSPM still produces recoveries for much higher values of γ. GeoSPM used t tests with a family-wise error corrected p value of 0.05, for kriging we applied a z-test with an uncorrected p value of 0.05, a null mean of 0.5 and a sample deviation obtained from the (positional) kriging variance estimate, as described in the section on "synthetic experiments: noise parameterization". Additional kriging recoveries are shown in Figures S21–S25 of supplemental note S3.5.

supplemental note S3.1, as are the results for the second term in the bivariate models in Figures S10 and S11 of supplemental note S3.2). A visual summary of the recovered binary maps underlying these performance curves—for the bivariate snowflake model and the high sampling regime—affords a further qualitative comparison between the two methods (Figure 5).

It is evident that GeoSPM offers superior efficiency across most of the noise range in all models and on all metrics.

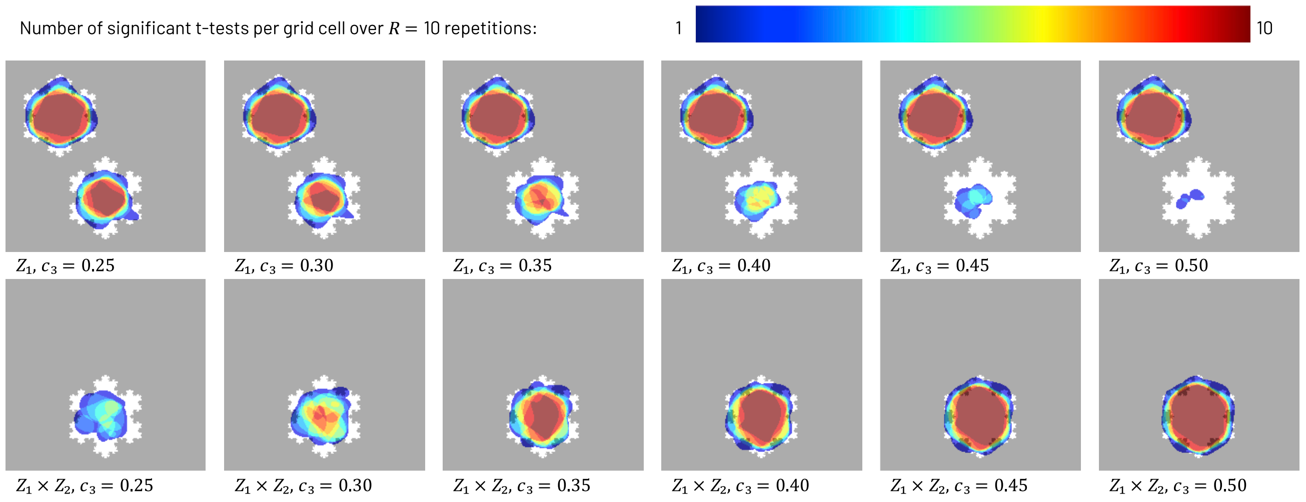

Figure 6. Recoveries produced by GeoSPM for the synthetic interaction model across $R = 10$ repetitions for variable Z_1 in the top row and term $Z_1 \times Z_2$ in the bottom row, with $N = 15{,}000$ samples

Grid cells that lie in the target region are shown in white, those outside in gray. The number of significant tests out of 10 repetitions is superimposed in color for each grid cell: dark blue indicates at least one significant test and dark red indicates the maximum number of 10, while cells with no significant test did not receive any color. Starting with a low value for the interaction effect c_3 on the left, recovery of the interaction term $Z_1 \times Z_2$ in region R_3 is weak, while recovery for variable Z_1 in the same region is stronger. This correlates with the fact that observations $(1,\ 1)$ occur with only a slightly elevated probability $p_3 = 0.6$ compared with their null probability of 0.525 when c_3 equals 0 in the same setting. As c_3 increases toward the right, recovery in the same region for term $Z_1 \times Z_2$ increases ($p_3 = 0.725$ at the right), while recovery for variable Z_1 decreases (probability $p_1 = 0.125$ at the right for observing $(1,\ 0)$, which is half of what it would be if there was no interaction effect). GeoSPM used t tests with a family-wise error corrected p value of 0.05.

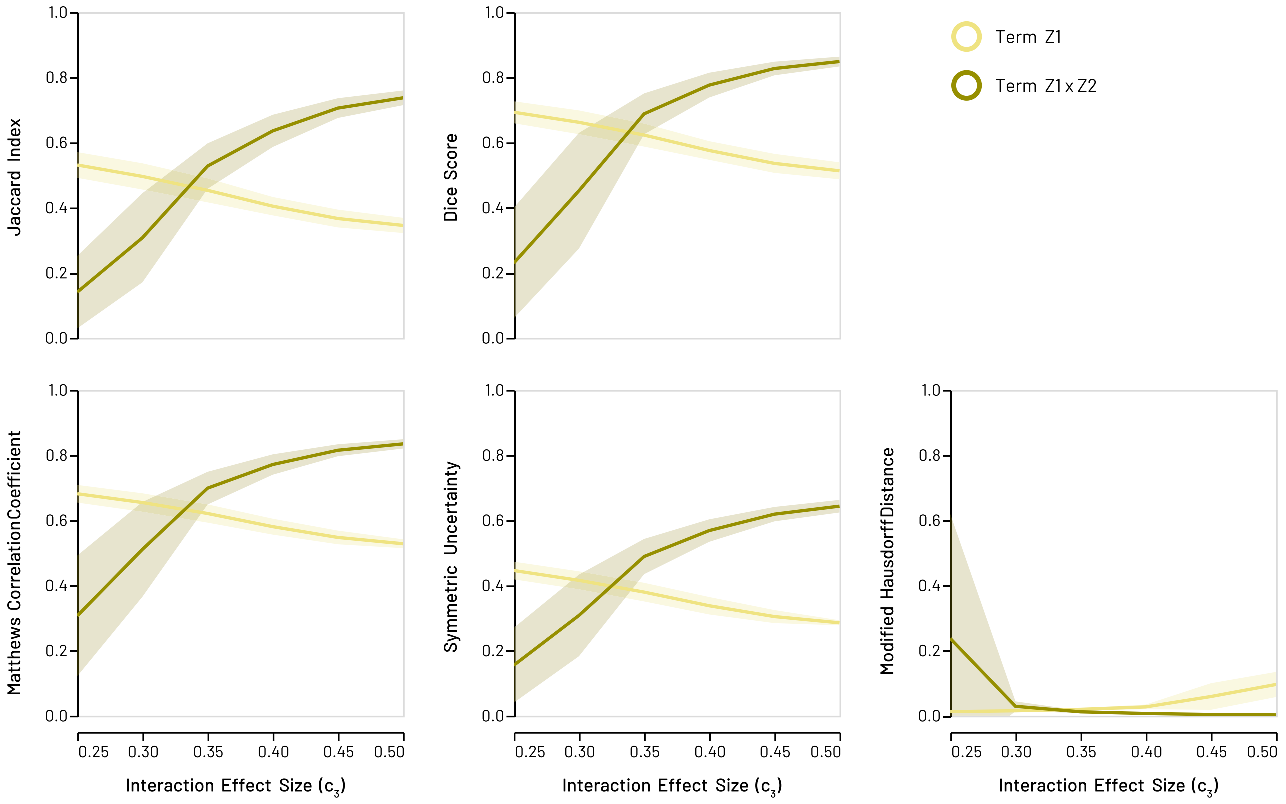

Figure 7. Synthetic snowflake interaction model: recovery scores for SPM model variable Z_1 and term $Z_1 \times Z_2$ with N = 15,000 samples
Lines denote the mean score across 10 random model realizations, shaded areas its SD to either side of the mean. We increase the approximate interaction effect in region R_3 of the grid from left to right, so that the probability of observing $(1, 1)$ grows while the probability of observing $(1, 0)$ or $(0, 1)$ shrinks (the probability of observing $(0, 0)$ stays the same). As a result, scores increase for the interaction term $Z_1 \times Z_2$ as it captures more of the overall variance, whereas scores for variable Z_1 decrease, until the only significant recovery occurs in region R_1, which represents half of the target for Z_1 and explains why the overall decrease saturates.

GeoSPM models generally remain stable at higher levels of noise than kriging. Both GeoSPM and kriging exhibit sensitivity to the sampling regime, both in terms of variability and stability, but the effects are dwarfed by the difference between the two approaches. The type of ground truth has negligible impact. In addition, neither changing the (cross-) covariance function used for kriging from a Matérn function to a Gaussian nor applying a different regime for dealing with coincident observations—such as averaging—yields a discernible improvement to the performance of kriging in this context (see Figure S12 in supplemental note S3.3).

Additional results based on an extended selection of covariance models for kriging show comparable outcomes and are similar to those presented in Figures 3, 4, and 5, as documented in Figures S13–S20 in supplemental note S3.4 and Figures S21–S25 in supplemental note S3.5. For a more in-depth view of the behavior of kriging parameters and variograms, refer to supplemental note S3.6 and S3.7.

The recoveries obtained from simulations of the interaction model clearly show GeoSPM's ability to detect an interaction between two spatially distributed factors, even toward the lower end of the approximate interaction effect size range (Figure 6). Plots of the same five indices above demonstrate successful retrieval for these interaction simulations quantitatively (Figure 7). As we increase the size of the approximate interaction effect c_3, retrieval results for the interaction term $Z_1 \times Z_2$ approach those of the previous, noise-free bivariate snowflake model (setting aside the different sampling regimes). At the same time, recovery for variable Z_1 decreases in the interaction region R_3 (but not elsewhere), as the interaction term explains more variance. Once the recovery for variable Z_1 in region R_3 has vanished, the corresponding retrieval scores are about half of those for the same term in the noise-free model, which agrees with our expectation, because only one of two snowflake shapes in the target are still retrieved at that stage.

UK Biobank models

In real-world scenarios there is usually no explicit ground truth against which an inference can be tested: the conclusion rests on the integrity of the underlying statistical assumptions. Our illustrative analysis of UK Biobank data[24] therefore does not seek to quantify GeoSPM's fidelity but to demonstrate its potential utility in the medical realm. We focus on two aspects: the derivation of marginalized spatial maps that disentangle a factor of interest from a set of (interacting) confounders, and the use of

CellPress
OPEN ACCESS

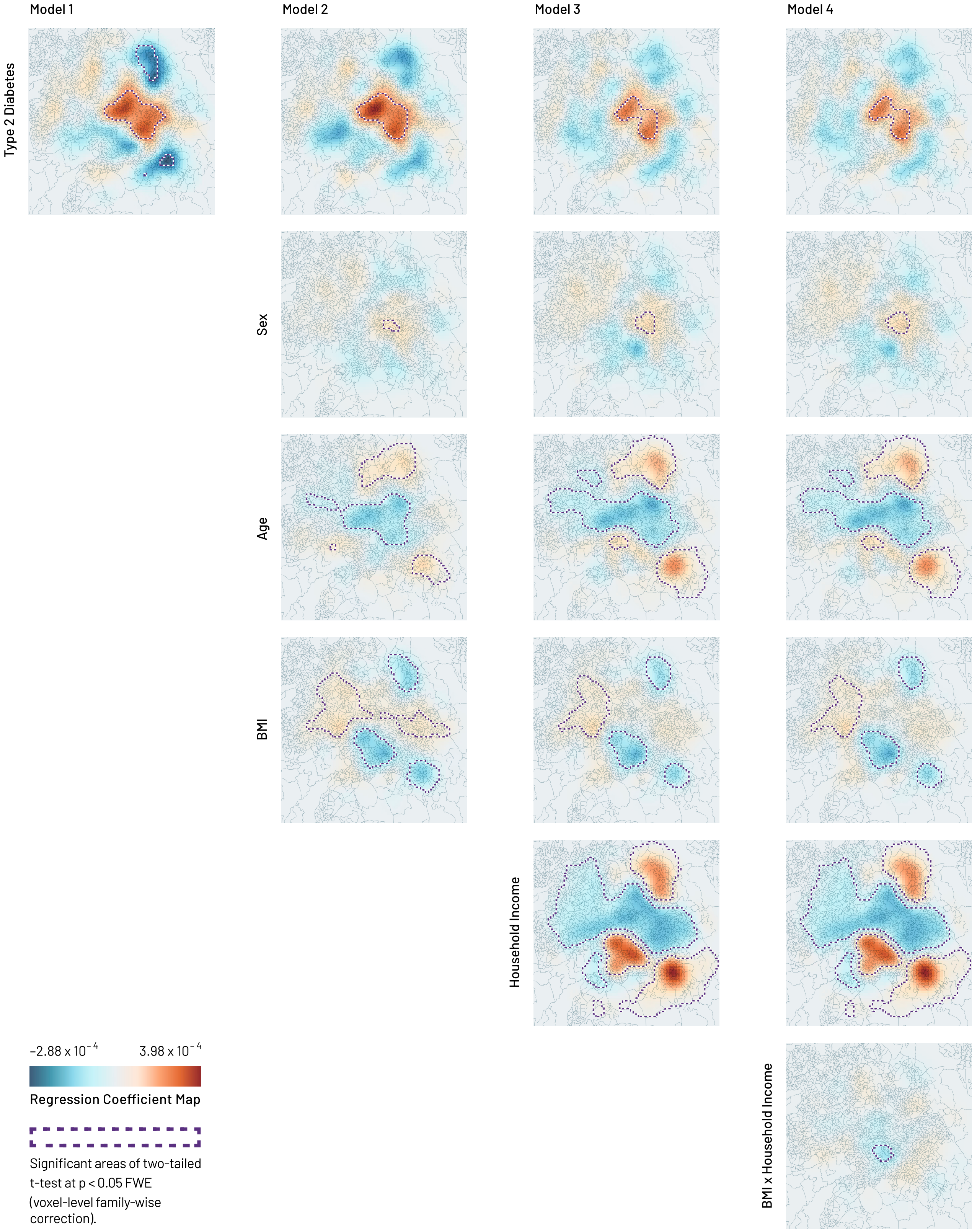

Figure 8. GeoSPM results for the four UK Biobank models of Birmingham (one column per model)
Geographic regression coefficient maps are shown with outlines of significant areas in the corresponding two-tailed t test at $p < 0.05$ FWE (voxel-level family-wise correction). The smoothing parameter value is 7,000 m.

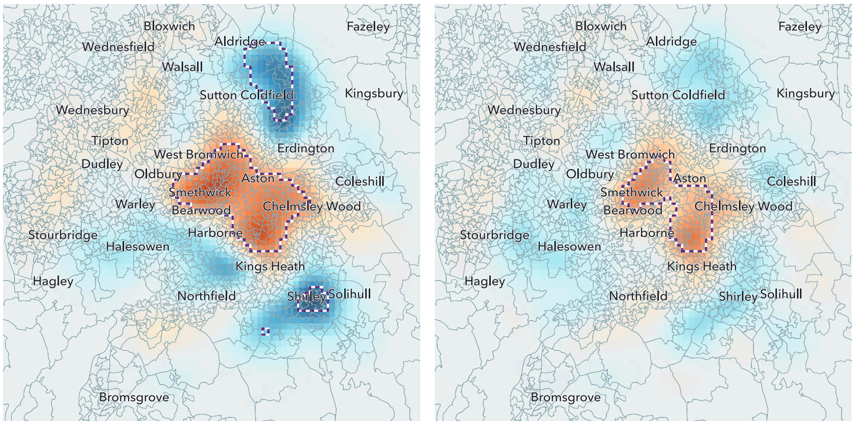

Diabetes regression coefficient map (model 1)

Diabetes regression coefficient map (model 4)

Figure 9. Geographic regression coefficient maps with location names for a single run of UK Biobank models 1 and 4

Model 1 is a univariate model of diabetes, model 4 adds sex, age, BMI, household income, and an interaction term BMI × household income. Outlines show significant areas in the corresponding two-tailed t test at $p < 0.05$ FWE (voxel-level family-wise correction). The smoothing parameter value is 7,000 m. The color map scale is the same as in Figure 8.

conjunction analysis to identify regions jointly modulated by multiple spatially organized factors.

The propensity to develop type 2 diabetes is related to age, sex, BMI, and household income, among other factors: a known pattern clearly replicated in UK Biobank. A map of diabetes may therefore reflect not just the propensity to develop the disease but also the spatial structure of associated factors, both causal and incidental. If we are pursuing a previously unknown spatial factor—pollution, for example,[32–34]—we would wish to void our diabetes map of known confounders, yielding a spatial distribution of fully marginalized propensity.

We demonstrate GeoSPM on individual-level UK Biobank data drawn from Birmingham. Figure 8 presents the regression coefficient maps and significant t test areas for four separate models of diabetes with incrementally greater numbers of covariates. The first, univariate, model of diabetes (model 1) reveals an extensive concentric organization, positive in the center and negative in the periphery, especially in the north and south. The map becomes more tightly circumscribed with the addition of sex, age, and BMI in model 2: the two negative areas in the north and south are no longer significant, and a stronger negative region emerges west of the center. With the addition of further covariates and their interactions, the spatial structure of diabetes that remains unexplained converges on a set of focal, central regions, displayed in detail in comparison with the univariate model in Figure 9. Here the regional expression of diabetes is not explained by the modeled covariates, suggesting the presence of other factors in play to be subsequently investigated. In general, the ensemble of significant areas for each model indicates the spatial structure that remains unexplained for the corresponding set of covariates, while the intensity and sign of each regression coefficient map represent the degree of spatial association of its covariate in the ensemble. With this in mind, the individual maps for diabetes represent a spatial distribution of propensity marginalized against the other covariates, but not an absolute rate of disease.

We can now also examine the *conjunctions* of multiple maps, not necessarily derived from the same model, within a second-level analysis. Conjunctions are here simply the intersections of two or more thresholded t maps, identifying areas where the regression coefficients and their associated variables are jointly significant. Applied to the outputs of our most complex model above, the approach and resulting conjunctions are shown in Figure 10. Pairwise conjunctions show a single region where diabetes and male sex are colocalized; a distinct region where diabetes and age are inversely associated; a very narrow region with an inverse relation between diabetes and BMI; and a single region where diabetes is inversely related to household income. Finally, a three-way conjunction identifies a region where diabetes is spatially associated with younger age, male sex, and lower income (Figure 11). Such conjunction maps identify regions where two or more variables of interest are significantly expressed together, representing subpopulations whose intersectionally characteristic features may inform responsive action or further investigation.

This concludes our illustration of GeoSPM. Note that the fact that GeoSPM was able to identify significant regionally specific effects provides a provisional form of predictive validity; under the assumption that these effects were present in the population—and could therefore be used to predict response variables.

DISCUSSION

We propose, implement, and validate an approach to drawing spatial inferences from sparse clinical data, extending to geostatistics a mature, principled framework for topological inference—SPM—that is well established in the realm of brain imaging. Compared with kriging, GeoSPM combines similar fidelity under optimal conditions with substantially less sensitivity to noise and under-sampling, greater robustness to failure, faster computation, graceful handling of multiple scales of spatial variation, and formal inferential support. Its simplicity and accessibility facilitate widespread application of the comprehensive software implementation we have provided, built on the validated SPM open-source codebase, across a wide range of applications in medicine and beyond. Here, we consider six points concerning the application, extension, and limitations of our approach.

First, GeoSPM is applicable to problems of topological spatial inference, whose formulation conforms to the minimal assumptions of the underlying statistical framework. The types of data, the choice of model evaluated at each point, and the size and density of the evaluated grid are not under

CellPress
OPEN ACCESS

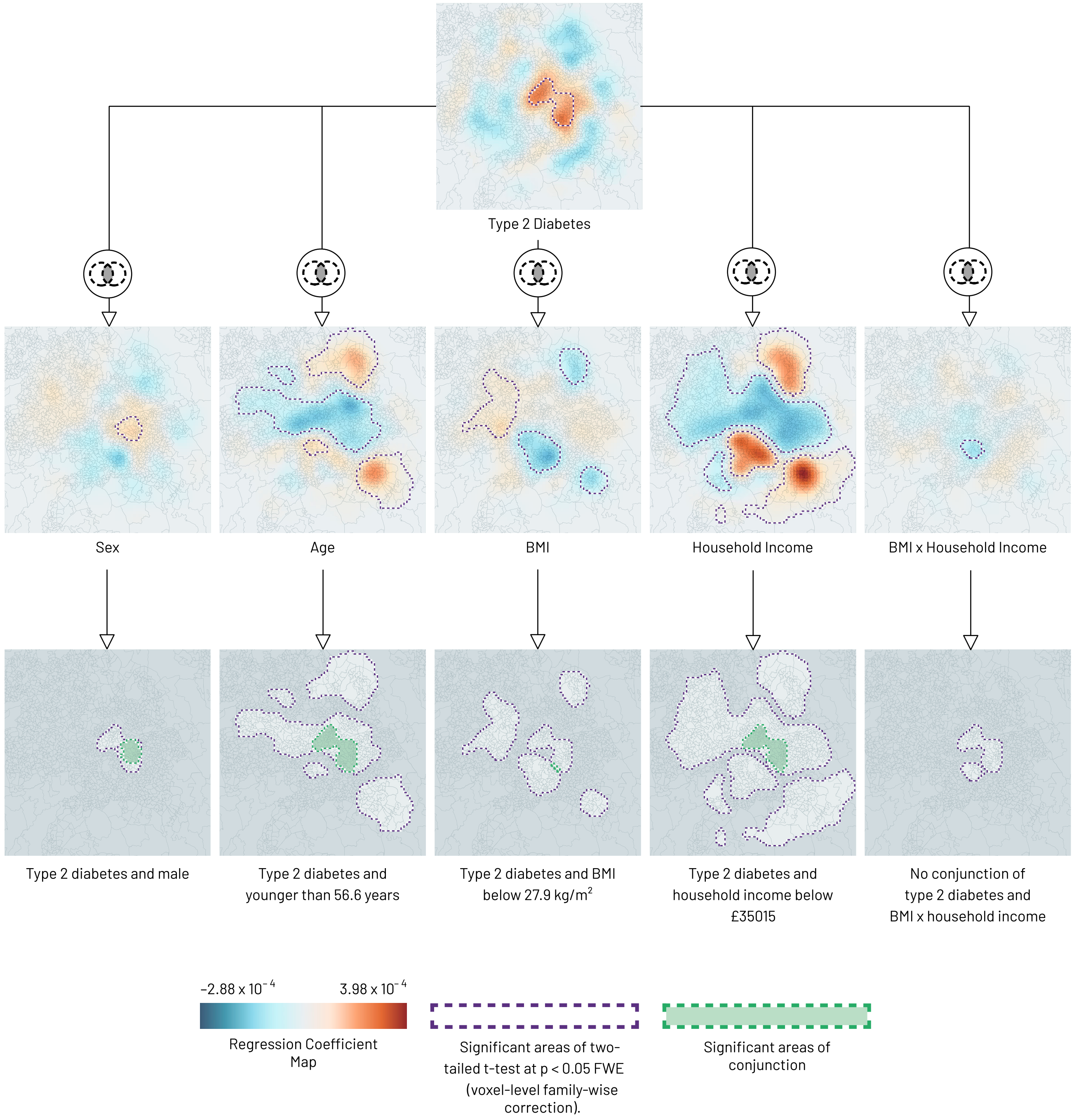

Figure 10. Binary conjunctions of geographic regression significance maps for a single run of UK Biobank model 4

A binary conjunction is formed of the significant areas of a two-tailed t test at $p < 0.05$ FWE (voxel-level family-wise correction) between type 2 diabetes and, in turn, sex, age, BMI, household income, and BMI × household income. Purple outlines show significant areas in the two-tailed t test of each variable, green outlines show significant areas of conjunction: significant areas of conjunction arise in diabetes combined with each of sex (male), age (younger than 56.6 years), BMI (below 27.9 kg/m^2), and household income (below £35,015). No significant areas of conjunction exist for diabetes and BMI × household income. Locations shown in darker gray tone are not significant for any of the variables. The smoothing parameter value is 7,000 m.

any strong constraint. Eliminating the spatial dimension allows each point-wise model to be more flexible than the data or computational resource could otherwise sustain. The model could even be complicated spatially, extending to encompass a local patch within otherwise the same framework. This is a key strength in medical applications, where a spatial effect typically needs to be disentangled from a wide array of others.

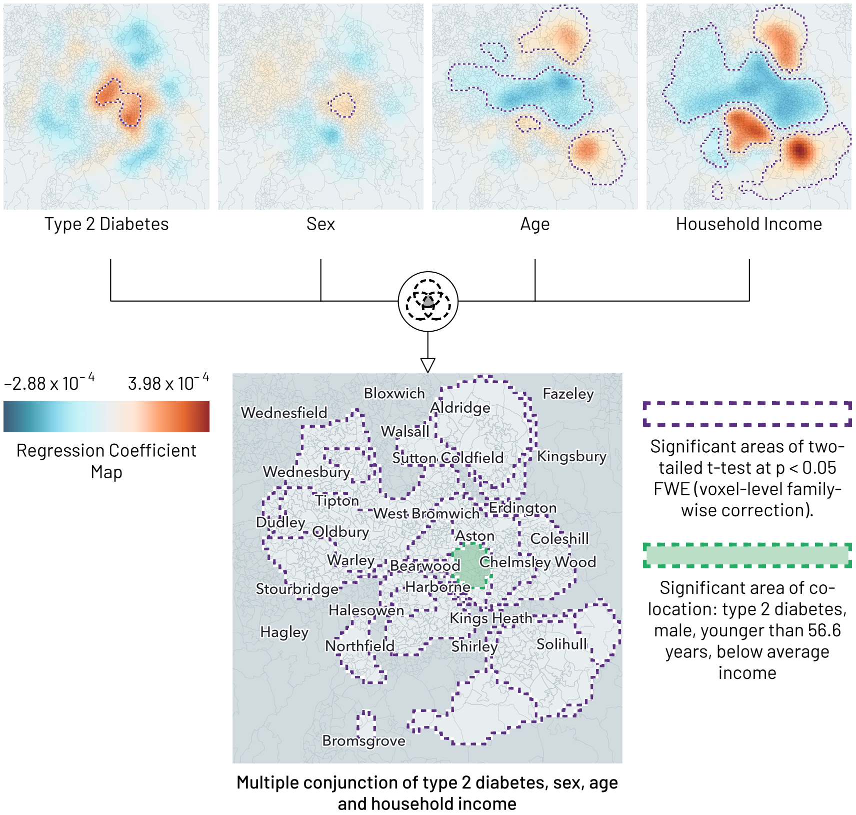

Figure 11. Example of a multiple conjunction (here quaternary) of geographic regression significance maps for a single run of UK Biobank model 4

A binary conjunction is formed of the significant areas of a two-tailed t test at $p < 0.05$ FWE (voxel-level family-wise correction) between type 2 diabetes and, in turn, sex, age, BMI, household income, and BMI × household income. Purple outlines show significant areas in the two-tailed t test of each variable, green outlines show significant areas of conjunction: we can identify a significant area where younger males of lower income are associated with having type 2 diabetes in Birmingham. The smoothing parameter value is 7,000 m.

Second, although here prototyped on temporally stationary data, GeoSPM can be configured with time instead of the spatial scale in the third dimension, enabling graceful modeling of both spatial and temporal correlations. This has been used, for example, in the context of electrophysiology[35] where extra dimensions can include peristimulus time or, indeed, fast oscillatory frequencies. The effects of manipulating noise and spatial dependencies can then be evaluated across individual time series. Equally, the third dimension could be used for multimodal data projected within the same grid, informing the inference by multiple sampling modalities.

Third, the smoothing parameter may be constrained by prior knowledge or independent estimation from the data, even if evaluating a set of models over a plausible range is arguably the most robust approach. One may alternatively rely on the properties of the inferred maps, as suggested in our validation analyses. All competing spatial modeling frameworks rely on chosen parameters to some degree; ours is reduced to a single readily interpretable one.

Fourth, no model could perfectly remedy defects in the data itself, such as inadequate or biased coverage. The former can be mitigated by confining inference to spatial locations exhibiting sufficient sampling density; the latter, analogously to structured missingness, is not easily remediable within this or any other inferential framework, and presents no more or less of a problem.

Fifth, GeoSPM, like SPM itself, is a platform for standard frequentist statistical inference, revealing the organization of spatially structured variables without causal implications of any kind. But, also like SPM, it is open both to Bayesian extensions, and causal modeling upstream or downstream of the core framework. There are many ways of querying data, both with classical mass univariate and Bayesian analyses of this kind. Although not illustrated here, model comparison using the *F*-statistic is a common application that could be enabled by GeoSPM. For example, one could ask whether household income has an effect on the regional prevalence of diabetes, having accounted for other demographic variables, by comparing (general linear) models that do and do not include household income as an explanatory variable.

Finally, the SPM approach, in any formulation, is designed for topological inference, not discrimination between distributed spatial patterns, which may also arise in healthcare, and requires explicit modeling of spatial interactions that only a multivariate model could conceivably deliver. Indeed, such use would violate the underlying assumption of benign regional dependence, as do analogous attempts in the domain of lesion-deficit mapping of the brain.[36] GeoSPM maps may nonetheless be used to select features where the fragility of the multivariate model, or the applicable data regime, compel it.

SUPPLEMENTAL INFORMATION

Supplemental information can be found online at https://doi.org/10.1016/j.patter.2022.100656.

ACKNOWLEDGMENTS

This work is aligned with a project on ''Novel methods to explore the value of cognitive health in a place'' supported by the Health Foundation, an independent charity committed to bringing about better health and health care for people in the UK. H.E., A.N., D.H., and P.N. are supported by the NIHR UCLH Biomedical Research Centre. M.S.P. is supported by the Health Foundation. M.R. is supported by the National Institute for Health Research (NIHR) Senior Investigator Award/NF-SI-0512-10033. A.J., G.R., and P.N. are supported by Wellcome (213038). The views expressed are those of the authors and not necessarily those of the NIHR, the Department of Health and Social Care, the Health Foundation, or Wellcome. The funders had no role in study design,

data collection and analysis, decision to publish, or preparation of the manuscript.

AUTHOR CONTRIBUTIONS

Conceptualization, P.N., H.E., and A.J.; methodology, P.N., H.E., A.J., and K.F.; software, H.E.; validation, P.N., H.E., A.J., and M.S.P.; formal analysis, H.E.; investigation, H.E. and A.N.; resources, P.N.; data curation, H.E.; writing – original draft, P.N. and H.E.; writing – review & editing, P.N., H.E., A.J., K.F., M.R., M.S.P., D.H., and G.R.; visualization, H.E. and P.N.; supervision, P.N.; funding acquisition, P.N. and M.R.

DECLARATION OF INTERESTS

The authors declare no competing interests.

Received: May 31, 2022
Revised: July 1, 2022
Accepted: November 11, 2022
Published: December 9, 2022

REFERENCES

1. Anselin, L. (1995). Local indicators of spatial association—LISA. Geogr. Anal. *27*, 93–115. https://doi.org/10.1111/j.1538-4632.1995.tb00338.x.
2. Kulldorff, M. (1997). A spatial scan statistic. Commun. Stat. Theory *26*, 1481–1496. https://doi.org/10.1080/03610929708831995.
3. Shepard, D. (1968). A two-dimensional interpolation function for irregularly-spaced data. In Proceedings of the 1968 23rd ACM National Conference, pp. 517–524. https://doi.org/10.1145/800186.810616.
4. Rosenblatt, M. (1956). Remarks on some nonparametric estimates of a density function. Ann. Math. Stat. *27*, 832–837. https://doi.org/10.1214/aoms/1177728190.
5. Wand, M.P., and Jones, M.C. (1994). Kernel Smoothing (CRC Press). https://doi.org/10.1007/978-1-4899-4493-1.
6. Anselin, L. (1988). Spatial Econometrics: Methods and Models (Springer Science & Business Media). https://doi.org/10.1007/978-94-015-7799-1.
7. Brunsdon, C., Fotheringham, S., and Charlton, M. (1998). Geographically weighted regression. J. Roy. Stat. Soc. D-Sta *47*, 431–443. https://doi.org/10.1111/1467-9884.00145.
8. Briggs, D.J., Collins, S., Fischer, P., Kingham, S., Lebret, E., Pryl, K., Van Reeuwijk, H., Smallbone, K., and Van Der Veen, A. (1997). Mapping urban air pollution using GIS: a regression-based approach. Int. J. Geogr. Inf. Sci. *11*, 699–718. https://doi.org/10.1080/136588197242158.
9. Goovaerts, P. (1997). Geostatistics for Natural Resources Evaluation (Oxford University Press on Demand).
10. Stein, M.L. (1999). Interpolation of Spatial Data: Some Theory for Kriging (Springer Science & Business Media). https://doi.org/10.1007/978-1-4612-1494-6.
11. Breslow, N.E., and Clayton, D.G. (1993). Approximate inference in generalized linear mixed models. J. Am. Stat. Assoc. *88*, 9–25. https://doi.org/10.1080/01621459.1993.10594284.
12. Hastie, T., and Tibshirani, R. (1986). Generalized additive models. Stat. Sci. *1*, 297–310. https://doi.org/10.1214/ss/1177013604.
13. Kammann, E.E., and Wand, M.P. (2003). Geoadditive models. J. Roy. Stat. Soc. C.–App. *52*, 1–18. https://doi.org/10.1111/1467-9876.00385.
14. Banerjee, S., Carlin, B.P., Gelfand, A.E., and Banerjee, S. (2003). Hierarchical Modeling and Analysis for Spatial Data (Chapman and Hall/CRC). https://doi.org/10.1201/9780203487808.
15. Lawson, A.B. (2018). Bayesian Disease Mapping: Hierarchical Modeling in Spatial Epidemiology, Third edition (CRC Press). https://doi.org/10.1201/9781351271769.
16. Diggle, P.J., Tawn, J.A., and Moyeed, R.A. (1998). Model-based geostatistics. J. Roy. Stat. Soc. C.–App. *47*, 299–350. https://doi.org/10.1111/1467-9876.00113.
17. Diggle, P.J., and Ribeiro, P.J. (2007). Model-based Geostatistics (Springer). https://doi.org/10.1007/978-0-387-48536-2.
18. Gelfand, A.E., Kim, H.-J., Sirmans, C.F., and Banerjee, S. (2003). Spatial modeling with spatially varying coefficient processes. J. Am. Stat. Assoc. *98*, 387–396. https://doi.org/10.1198/016214503000170.
19. Da Silva, A.R., and Fotheringham, A.S. (2016). The multiple testing issue in geographically weighted regression. Geogr. Anal. *48*, 233–247. https://doi.org/10.1111/gean.12084.
20. Yuan, Y., Cave, M., Xu, H., and Zhang, C. (2020). Exploration of spatially varying relationships between Pb and Al in urban soils of London at the regional scale using geographically weighted regression (GWR). J. Hazard Mater. *393*, 122377. https://doi.org/10.1016/j.jhazmat.2020.122377.
21. Brunsdon, C., Fotheringham, A.S., and Charlton, M.E. (1996). Geographically weighted regression: a method for exploring spatial non-stationarity. Geogr. Anal. *28*, 281–298. https://doi.org/10.1111/j.1538-4632.1996.tb00936.x.
22. Stewart Fotheringham, A., Charlton, M., and Brunsdon, C. (1996). The geography of parameter space: an investigation of spatial non-stationarity. Int. J. Geogr. Inf. Syst. *10*, 605–627. https://doi.org/10.1080/02693799608902100.
23. Friston, K.J., Worsley, K.J., Frackowiak, R.S., Mazziotta, J.C., and Evans, A.C. (1994). Assessing the significance of focal activations using their spatial extent. Hum. Brain Mapp. *1*, 210–220. https://doi.org/10.1002/hbm.460010306.
24. Sudlow, C., Gallacher, J., Allen, N., Beral, V., Burton, P., Danesh, J., Downey, P., Elliott, P., Green, J., Landray, M., et al. (2015). UK biobank: an open access resource for identifying the causes of a wide range of complex diseases of middle and old age. PLoS Med. *12*, e1001779. https://doi.org/10.1371/journal.pmed.1001779.
25. Worsley, K.J. (2000). An Unbiased Estimator for the Roughness of a Multivariate Gaussian Random Field (Department of Mathematics and Statistics, University of McGill). https://www.math.mcgill.ca/keith/smoothness/techrept.pdf.
26. Kiebel, S.J., Poline, J.-B., Friston, K.J., Holmes, A.P., and Worsley, K.J. (1999). Robust smoothness estimation in statistical parametric maps using standardized residuals from the general linear. Neuroimage *10*, 756–766. https://doi.org/10.1006/nimg.1999.0508.
27. Friston, K.J., Josephs, O., Zarahn, E., Holmes, A.P., Rouquette, S., and Poline, J. (2000). To smooth or not to smooth? Bias and efficiency in fMRI time-series analysis. Neuroimage *12*, 196–208. https://doi.org/10.1006/nimg.2000.0609.
28. Worsley, K.J., Marrett, S., Neelin, P., and Evans, A.C. (1996). Searching scale space for activation in PET images. Hum. Brain Mapp. *4*, 74–90. https://doi.org/10.1002/(SICI)1097-0193(1996)4:1<74::AID-HBM5>3.0.CO;2-M.
29. Pebesma, E.J. (2004). Multivariable geostatistics in S: the gstat package. Comput. Geosci. *30*, 683–691. https://doi.org/10.1016/j.cageo.2004.03.012.
30. Dubuisson, M.-P., and Jain, A.K. (1994). A modified Hausdorff distance for object matching. In Proceedings of 12th International Conference on Pattern Recognition, *1*, pp. 566–568. https://doi.org/10.1109/ICPR.1994.576361.
31. Taha, A.A., and Hanbury, A. (2015). Metrics for evaluating 3D medical image segmentation: analysis, selection, and tool. BMC Med. Imag. *15*, 1–28. https://doi.org/10.1186/s12880-015-0068-x.
32. Yang, B.-Y., Fan, S., Thiering, E., Seissler, J., Nowak, D., Dong, G.-H., and Heinrich, J. (2020). Ambient air pollution and diabetes: a systematic review and meta-analysis. Environ. Res. *180*, 108817. https://doi.org/10.1016/j.envres.2019.108817.
33. Bowe, B., Xie, Y., Li, T., Yan, Y., Xian, H., and Al-Aly, Z. (2018). The 2016 global and national burden of diabetes mellitus attributable to $PM_{2\cdot5}$ air pollution. Lancet Planet. Health *2*, E301–E317. https://doi.org/10.1016/S2542-5196(18)30140-2.

34. Yongze, L., Xu, L., Shan, Z., Teng, W., and Han, C. (2019). Association between air pollution and type 2 diabetes: an updated review of the literature. Ther. Adv. Endocrinol. Metab. *10*, 1–15. https://doi.org/10.1177/2042018819897046.

35. Litvak, V., Mattout, J., Kiebel, S., Phillips, C., Henson, R., Kilner, J., Barnes, G., Oostenveld, R., Daunizeau, J., Flandin, G., et al. (2011). EEG and MEG data analysis in SPM8. Comput. Intel. Neurosc. *2011*, 852961. https://doi.org/10.1155/2011/852961.

36. Mah, Y.-H., Husain, M., Rees, G., and Nachev, P. (2014). Human brain lesion-deficit inference remapped. Brain *137*, 2522–2531. https://doi.org/10.1093/brain/awu164.

Patterns, Volume *3*

Supplemental information

GeoSPM: Geostatistical parametric mapping for medicine

Holger Engleitner, Ashwani Jha, Marta Suarez Pinilla, Amy Nelson, Daniel Herron, Geraint Rees, Karl Friston, Martin Rossor, and Parashkev Nachev

Supplemental Note

S1 Bibliographic analysis

Geospatial field bibliometrics were computed through a search of the titles and abstracts of the entire medical corpus from Microsoft Academic Graph from January 1990-March 2019 cited at least once (17.1 million papers), filtered by keyword string matching (non case-sensitive) within abstracts on the following terms: "geo*" & "map*" & "illness*|disease*|health*". This returned 1897 papers, with mean citations (normalised by the average citation count of a paper in that journal) 1.67 (sd 2.87).

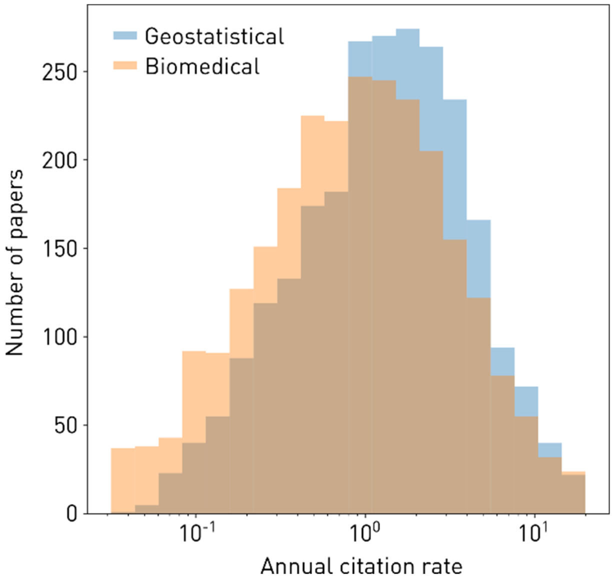

Figure S1. Overlapped histograms of the decimal log-transformed annual citation rates of 1897 identified journal papers at the intersection of spatial analysis and medicine cited more than once (blue), and an identically filtered random sample of non-spatial biomedical papers (orange), published between 1990 and 2019. The untransformed distributions are significantly different on a Mann-Whitney U test, p<0.001.

S2 Supplemental methods

S2.1.1 Synthetic data and generative models

We start by defining a spatial domain as a rectangular subset $D \coloneqq [0, a) \times [0, b) \subset \mathbb{R}^2$ for some $a, b \in \mathbb{N}^+$, and restrict a and b to positive integers so that

$$D = [0, a) \times [0, b) = \bigcup_{j=1}^{a} \bigcup_{k=1}^{b} [j-1, j) \times [k-1, k)$$

has a natural decomposition into $a \times b$ grid cells with coordinates $(j, k) \in D' \coloneqq \{1, \dots, a\} \times \{1, \dots, b\}$. Assuming that there are P binary factors in the generative model, the simulated response variables can be written as the components of a random vector $\boldsymbol{Z} = (Z_1, \dots, Z_P)^{\mathrm{T}} \in \{0,1\}^P$, which is sampled at

random grid cells $\boldsymbol{W} \in D'$ in the underlying space. Our data generation mechanism is based on the factorisation of the joint distribution of $\boldsymbol{Z}$ and $\boldsymbol{W}$ as

$$\Pr(\boldsymbol{Z}, \boldsymbol{W}; \boldsymbol{\theta}) = \Pr(\boldsymbol{Z} \mid \boldsymbol{W};\ \boldsymbol{\theta})\Pr(\boldsymbol{W})$$

so that the response variables $(Z_1, \ldots, Z_P)^{\mathrm{T}}$ are conditioned on location $\boldsymbol{W}$ with model parameters $\boldsymbol{\theta}$ fixed at $(\theta_1, \theta_2,\ \ldots)^{\mathrm{T}}$. $\boldsymbol{W}$ is distributed independently of $\boldsymbol{Z}$ and uniformly over D'.

The conditioning allows breaking down the distribution of $\boldsymbol{Z}$ spatially, by partitioning the grid D' into a small number of (not necessarily) continuous regions $R_k \subseteq D' : k = 0, \ldots, K-1$ for which local distributions $\mathrm{P}_k(\boldsymbol{Z}; \boldsymbol{\theta}) \coloneqq \Pr(\boldsymbol{Z} \mid \boldsymbol{W} \in R_k;\ \boldsymbol{\theta})$ can be specified for each region, k. As $P \in \{1,2\}$ for the models considered here, at most four probabilities are required for each of these local distributions. The partitions are based on arrangements of fractal shapes, shown in Figure S2 for the two bivariate models and in Figure S3 for the univariate models.

In these images, a single pixel represents a grid cell, and the shading indicates the distinct distributions $\mathrm{P}_k(\boldsymbol{Z}; \boldsymbol{\theta})$. The resolution of the bivariate models is 220 by 210 grid cells, whereas the resolution of the univariate models is 120 by 120 grid cells. Geometry for the fractal shapes is constructed by recursively substituting the edges of a (start) shape with a simple curve (Figure S4) and then rasterising the resulting polygon into the grid using MATLAB's poly2mask function. We wish to examine the effect of noise and interactions in our numerical experiments, which leads us to consider two distinct parameterisations of the distributions P_k.

S2.1 Parameterisation of $\mathbf{P}_k(\boldsymbol{Z}; \boldsymbol{\theta})$ for Examining Noise

The first parameterisation is expressed in terms of a function $\mathrm{p}_{noise}(\cdot)$ with parameters p and q:

$$\mathrm{P}_k(\boldsymbol{Z}; \boldsymbol{\theta}) = \mathrm{p}_{noise}(Z_1, Z_2;\ p, q), \qquad p = \theta_k, q = \theta_{k+K}$$

$\mathrm{p}_{noise}(\cdot)$ is summarised in Table S1. The parameters p and q are simply the values of the marginal probabilities $\mathrm{p}_{noise}(z_1 = 1)$ and $\mathrm{p}_{noise}(z_2 = 1)$ and are sufficient for defining $\mathrm{p}_{noise}(Z_1, Z_2;\ p, q)$ if $Z_1$and Z_2 are assumed to be independent.

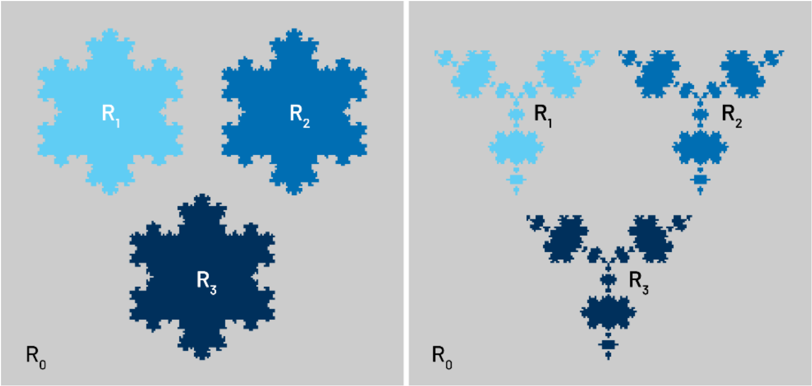

Figure S2. The four distinct regions $R_k : k = 0, \ldots, 3$, of the joint conditional probability $\Pr(\boldsymbol{Z} \mid \boldsymbol{W};\ \boldsymbol{\theta})$ for the snowflake model (left) and the anti-snowflake model (right).

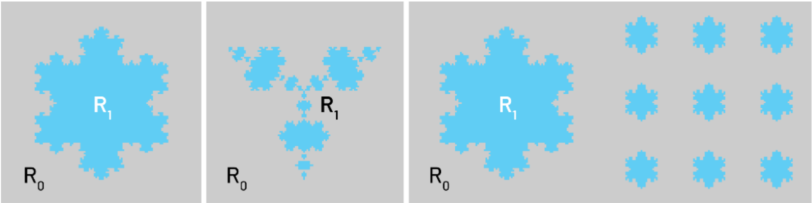

Figure S3. The two distinct regions $R_k : k = 0, 1$ of the conditional probability $\Pr(Z \mid \boldsymbol{W};\ \theta)$ for the univariate models: snowflake model (left), anti-snowflake model (middle) and snowflake field model (right).

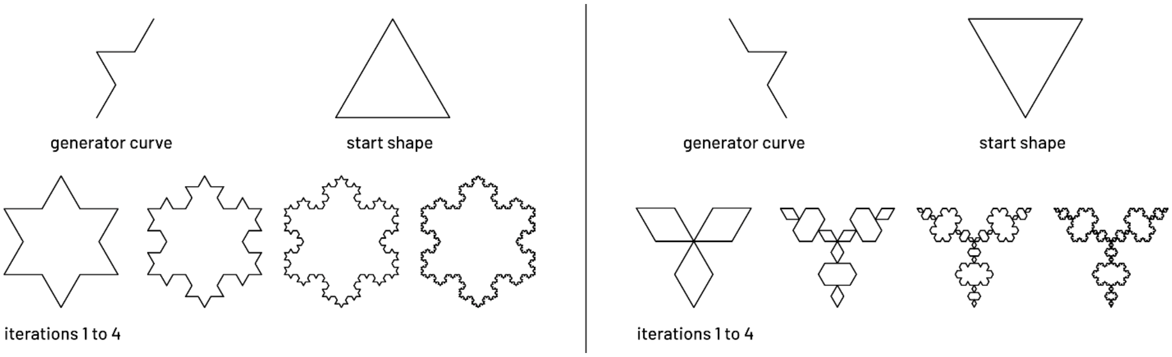

Figure S4. The geometric construction used for the fractal shapes. Koch snowflake on the left, Koch anti-snowflake on the right.

$\mathrm{p}_{noise}(Z_1, Z_2;\ p, q)$	$z_1 = 0$	$z_1 = 1$	$\mathrm{p}_{noise}(z_2)$
$z_2 = 0$	$(1-q)(1-p)$	$(1-q)p$	$1 - q$
$z_2 = 1$	$q(1-p)$	qp	q
$\mathrm{p}_{noise}(z_1)$	$1 - p$	p	1

Table S1. Probability table for the bivariate local distribution function $\mathrm{p}_{noise}(Z_1, Z_2;\ p, q)$

As we move in D from one region to the next, we simulate spatially distinct conditions of the variables $Z_1$and Z_2 by changing the regional expectations $\mathbb{E}_k[(Z_1, Z_2)]$ through $\mathrm{p}_{noise}(Z_1, Z_2;\ p, q)$: For the four regions of the bivariate models in Figure S2, the corresponding expectations are listed in Table S2, together with the respective values for p and q. In the absence of uncertainty, the models generate the expected values in each region exactly, thus R_0 *only*generates observations $(0,\ 0)$, R_1 *only* $(1,\ 0)$, and so on.

As more uncertainty (i.e., noise) is introduced—by adjusting the values for p and q—the overall pattern of observations still holds, but other values have a non-zero probability of occurrence: R_0 *mostly* generates observations $(0,\ 0)$, R_1 *mostly* $(1,\ 0)$, and so on, until a maximum level of uncertainty is reached and each observation is equally probable in every region. By expressing p and q in terms of a single parameter $\gamma \in [0, \dots, 0.5]$ (the last two columns in Table S2), we can easily vary the degree of observation noise from a spatially deterministic and regionally differentiated form, to one where all regional differentiation is lost (see Figure S5 for an example).

	$\mathbb{E}_k[(Z_1, Z_2)] \rightarrow \ldots$	$p \rightarrow \ldots$	$q \rightarrow \ldots$	$p(\gamma)$	$q(\gamma)$
R_0	(0,0)	0	0	γ	γ
R_1	(1,0)	1	0	$1-\gamma$	γ
R_2	(0,1)	0	1	γ	$1-\gamma$
R_3	(1,1)	1	1	$1-\gamma$	$1-\gamma$
$max.uncertainty$	(0.5,0.5)	0.5	0.5		

Table S2. Expected values of $Z_1$and Z_2 in each region of the Snowflake and Anti-Snowflake models shown in Figure 1 for the given values of p and q.

The parameter vector $\boldsymbol{\theta}$ in $\mathrm{P}_k(\boldsymbol{Z};\boldsymbol{\theta})$ for the bivariate models is determined by γ as shown in Table S2 and has the following structure:

$$\boldsymbol{\theta} = \boldsymbol{\theta}_\gamma, \qquad \boldsymbol{\theta}_\gamma \coloneqq [\gamma, 1-\gamma, \gamma, 1-\gamma, \gamma, \gamma, 1-\gamma, 1-\gamma]$$

We consider the deviation of observed values of $\boldsymbol{Z}$ when γ is non-zero from the expected values when γ is 0 as simulating noise induced by confounding variables that are not captured in the data. Its effect of degrading the observable spatial differentiation of the variables of interest is key in our analysis of the performance of GeoSPM, and so we treat γ as an independent variable in these numerical experiments.

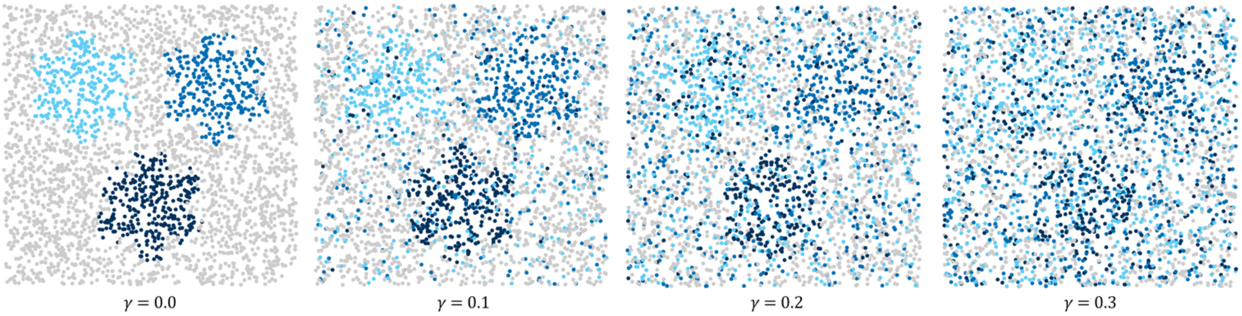

Figure S5. Random realisations of the bivariate Snowflake model for different levels of γ (from left to right).

Of course, real data also exhibit additive measurement noise. To simulate measurement noise an observation $\boldsymbol{Y} \in \mathbb{R}^P$ with location $\boldsymbol{X} \in D$ is derived from $\boldsymbol{Z}$ and $\boldsymbol{W}$ by adding random effects sampled from multidimensional uniform distributions:

$$\begin{aligned} &\boldsymbol{Y} = \boldsymbol{Z} + \boldsymbol{\zeta}, \qquad && \boldsymbol{\zeta} \sim \text{uniformly on } I_1 \times \ldots \times I_P, \qquad I_i = [0, 0.005] \\ &\boldsymbol{X} = \boldsymbol{W} + \boldsymbol{\omega}, && \boldsymbol{\omega} \sim \text{uniformly on } [0,1) \times [0,1) \end{aligned}$$

A practical benefit of applying 'spatial noise' $\boldsymbol{\omega}$ to $\boldsymbol{W}$ is that the probability of two randomly drawn elements $(\boldsymbol{y}_i, \boldsymbol{x}_i)$ and $(\boldsymbol{y}_j, \boldsymbol{x}_j)$ coinciding at the same location $\boldsymbol{x}_i = \boldsymbol{x}_j$ is minimised. This is relevant for the geostatistical method used for validation in these numerical experiments, because such collisions would produce singular, non-positive definite covariance matrices, when predicting observations and need to be removed from any data set prior to model estimation.

As GeoSPM only operates in terms of the discrete space D', it always applies the congruency $\boldsymbol{X} \equiv \boldsymbol{W}$ and so the added noise $\boldsymbol{\omega}$ has no effect. We will also consider an alternative method for resolving spatially coincident observations—by averaging observations from the same location—and provide corresponding results for kriging below.

We can now summarise the procedure for generating a spatially-referenced data set of size N and noise level γ_0 as follows:

1. Draw a uniform sample of N grid cell coordinates $\boldsymbol{w}_i \in \{1, \dots, a\} \times \{1, \dots, b\},\ i: 1, \dots, N$
2. For each grid cell coordinate $\boldsymbol{w}_i$ draw a sample $\boldsymbol{z}_i$ from $\mathrm{P}_k(\boldsymbol{Z}; \boldsymbol{\theta}_\gamma)$, where $\boldsymbol{w}_i \in R_k$
3. Obtain an observation $\boldsymbol{y}_i$ at location $\boldsymbol{x}_i$ by adding small amounts of random noise to $\boldsymbol{z}_i$ and $\boldsymbol{w}_i$:

$$\boldsymbol{y}_i = \boldsymbol{z}_i + \boldsymbol{\zeta}_i$$
$$\boldsymbol{x}_i = \boldsymbol{w}_i + \boldsymbol{\omega}_i$$

S2.1.2 Parameterisation of $\mathbf{P}_k(\boldsymbol{Z}; \boldsymbol{\theta})$ for Examining Interactions

A common feature of regression modelling is the inclusion of interaction terms, when there is reasonable belief that the marginal effect of one variable depends on the value of another. In a spatial setting, an interaction could be described as the degree to which the observation of a value of one variable is affected by the value of another variable at the same location. Therefore, GeoSPM's ability to detect interactions merits additional evaluation.

Here, a second parameterisation of the local distributions P_k can be motivated by interpreting the spatial response introduced earlier as a *concentration* instead of a measure of closeness. The constituent probabilities of the P_k are then the *regionally expected concentrations* of their respective observations $(Z_1, Z_2) \in \{(0,0), (1,0), (0,1), (1,1)\}$. As the response of local univariate regression models, these concentrations should have approximately *additive structure*, given the objective is to model interactions. To this end, we define the P_k as a function $\mathrm{p}_{interaction}(\cdot)$ with parameters p_0, c_1, c_2 and c_3, which we assign from a global parameter vector $\boldsymbol{\theta}$ for each region R_k:

$$\mathrm{P}_k(\boldsymbol{Z}; \boldsymbol{\theta}) = \mathrm{p}_{interaction}(Z_1, Z_2;\ p_0, c_1, c_2, c_3), \qquad p_0 = \theta_k, c_i = \theta_{k + iK}$$

Table S3 provides a definition of $\mathrm{p}_{interaction}(\cdot)$. The parameters c_i approximate the *effect sizes* induced by the observations of the variables (Z_1, Z_2) in the local univariate regression models, which—due to their binary values—correspond to the effect of Z_1, Z_2 and their interaction $Z_1 \times Z_2$ on the concentration or response. This is only an approximation of effect size, because of the non-linear fall-off of the Gaussian kernels used to synthesize the response.

$\mathrm{p}_{interaction}(Z_1, Z_2;\ p_0, c_1, c_2, c_3)$	$z_1 = 0$	$z_1 = 1$
$z_2 = 0$	p_0	$p_0 + c_1$
$z_2 = 1$	$p_0 + c_2$	$p_0 + c_1 + c_2 + c_3$

Table S3. Probability table for the bivariate local distribution function $\mathrm{p}_{interaction}(Z_1, Z_2;\ p_0, c_1, c_2, c_3)$

The parameters c_i define the respective probabilities relative to the parameter p_0, the probability of generating the observation $(0,\ 0)$. The c_i have a maximum range of $[-1, \ldots, 1]$, subject to the conditions that each p_i is a valid probability ($p_i \in [0, \ldots, 1]$) and that all the resulting probabilities add up to 1:

$$p_1 = p_0 + c_1$$

$$p_2 = p_0 + c_2$$

$$p_3 = p_0 + c_1 + c_2 + c_3$$

$$1 = 4p_0 + 2c_1 + 2c_2 + c_3$$

For the interaction experiments, we define the same four distinct regions $R_{0\ldots3}$ as for the bivariate snowflake model (shown on the left of Figure 1) in the noise parameterisation and vary the magnitude of the interaction effect c_3 in region R_3. In detail, in region R_0, all observations are equiprobable, representing the null state, as all three effects c_1, c_2 and c_3 are 0. R_1 and R_2 are regions where we either observe a non-zero effect c_1 for variable Z_1 or a non-zero effect c_2 for variable Z_2 but no effect that corresponds to their interaction.

Finally, we construct region R_3 to model an interaction effect c_3 at different intensities while keeping p_0 constant at a non-zero level and assuming $c_1 = c_2$. Based on (arbitrarily) setting p_0 to a small non-zero value of 0.025 and obeying all constraints, c_3 can range between 0 and 0.9. Given p_0 and a value for c_3 we can derive $c_1 = c_2 = \frac{1 - 4p_0 - c_3}{4}$. The regional probabilities chosen for the experiments are summarised in Table S4 (including null values for when $c_3 = 0$), where we picked 6 equally spaced settings between 0.25 and 0.5 for the interaction effect c_3 in R_3.

	R_0	R_1	R_2	$R_{3[null]}$	$R_{3[0.25]}$	$R_{3[0.3]}$	$R_{3[0.35]}$	$R_{3[0.4]}$	$R_{3[0.45]}$	$R_{3[0.5]}$
p_0	0.25	0.125	0.125	0.025	0.025	0.025	0.025	0.025	0.025	0.025
c_1	0	0.25	0	0.225	0.1625	0.15	0.1375	0.125	0.1125	0.1
c_2	0	0	0.25	0.225	0.1625	0.15	0.1375	0.125	0.1125	0.1
c_3	0	1	1	0	0.25	0.3	0.35	0.4	0.45	0.5
p_1	0.25	0.375	0.125	0.25	0.1875	0.175	0.1625	0.15	0.1375	0.125
p_2	0.25	0.125	0.375	0.25	0.1875	0.175	0.1625	0.15	0.1375	0.125
p_3	0.25	0.375	0.375	0.475	0.6	0.625	0.65	0.675	0.7	0.725

Table S4. Approximate effect sizes and derived probabilities for each region of the interaction experiments. Region $R_3$is the only region that is varied across experiments, by increasing the magnitude of the interaction effect c_3 specified in square brackets.

As with the noise parameterisation, a data set of N observations can be generated in a few simple steps. Again, we add random effects $\boldsymbol{\zeta}$ and $\boldsymbol{\omega}$ from multidimensional uniform distributions as defined above:

1. Draw a uniform sample of N grid cell coordinates $\boldsymbol{w}_i \in \{1, \ldots, a\} \times \{1, \ldots, b\},\ i: 1, \ldots, N$
2. For each grid cell coordinate $\boldsymbol{w}_i$ draw a sample $\boldsymbol{z}_i$ from $\mathrm{P}_k(\boldsymbol{Z}; \boldsymbol{\theta}_{interaction})$, where $\boldsymbol{w}_i \in R_k$ and $\boldsymbol{\theta}_{interaction}$ is the vector of combined regional parameters p_0, c_1, c_2 and c_3 for all four regions.

3. Obtain an observation $\boldsymbol{y}_i$ at location $\boldsymbol{x}_i$ by applying small amounts of random noise to $\boldsymbol{z}_i$ and $\boldsymbol{w}_i$:

$$\boldsymbol{y}_i = \boldsymbol{z}_i + \boldsymbol{\zeta}_i$$
$$\boldsymbol{x}_i = \boldsymbol{w}_i + \boldsymbol{\omega}_i$$

This completes our description of the synthetic data used to establish the face validity of GeoSPM.

S2.2 UK Biobank data

UK Biobank provides a large collection of health and genetic information for its prospective cohort of more than 500 000 participants recruited between 2006 and 2010 with assessment centres throughout Great Britain (https://www.ukbiobank.ac.uk/) [24].

We extracted a set of variables from UK Biobank in a region defined by a 35 km by 35 km square (spanning from 388000E, 423000N in the south-west corner to 269000E, 304000N in its north-east corner, in co-ordinates of the Ordnance Survey National Grid). The variables were sex (field 31), age (field 21022), body mass index (BMI, field 21001), household income (field 738) and the location of the participants (fields 20074 and 20075). Location information is based on the address to which the participants invitation was sent. Address verification and geo-coding was performed by UK Biobank using commercial software from Experian PLC and locations are provided at 100 metre and 1000 metre resolutions, the latter being the resolution available to us. All location co-ordinates use the Ordnance Survey reference. UK Biobank provides one or more temporal instances for certain fields. For such fields, the value of the earliest instance was chosen, which was the case for BMI and household income. In addition, ICD-10 and ICD-9 diagnosis codes were gathered from a separate hospital inpatient data table named HESIN_DIAG provided through field 41259. From these diagnosis codes we defined an indicator variable for type 2 diabetes, whose value was set to 1 whenever a participant had a record of either an ICD-10 code in block E11 ("type 2 diabetes mellitus") or at least one of a handful of relevant ICD-9 codes as specified in Table S7 in Supplemental Note. The number of participants with available data for all selected variables in the selected area of Birmingham was 18193, resulting in a collection of as many individual locations and associated individual observations that was used in the subsequently described analysis.

As a preliminary sanity check for the presence and degree of associativity, the diabetes indicator variable was entered as the response variable into a multiple Bayesian logistic regression model with a ridge prior. Sex, age, BMI, household income and the interaction between BMI and household functioned as predictors. Age, BMI and household income were centred at 0 and divided by their respective sample standard deviations. The interaction term was then formed as a simple multiplication. The model was evaluated by BayesReg version 1.9.1 [S1] in MATLAB. BayesReg uses a Markov Chain Monte Carlo (MCMC) Gibb's sampler. Posterior parameters were estimated from a single chain of 250000 samples (after a burn-in period of the same number of samples), of which only every 5th sample was used for computing the estimate. The posterior means of the regression coefficients and their credible intervals were as follows:

Predictor	Coefficient Posterior Mean ± SD	95% Credible Interval	t-Statistic	ESS
Sex	0.748 ± 0.054	(0.642 to 0.855)	13.79	82.2
Age	0.313 ± 0.029	(0.257 to 0.371)	10.73	83.5
BMI	0.719 ± 0.025	(0.670 to 0.769)	28.64	71.5
Household Income	-0.344 ± 0.036	(-0.416 to -0.274)	-9.50	61.9
BMI x Household Income	0.053 ± 0.028	(-0.001 to 0.108)	1.93	73.0

Table S5. Results of the preliminary Bayesian logistic ridge regression analysis of the UK Biobank diabetes data set extracted for Birmingham.

The results showed that there is a reasonably strong association between type 2 diabetes and all main terms, but evidence for an interaction between BMI and household income appears to be weak. On the basis of this preliminary analysis, we directed our attention to the spatial variability of diabetes and the question of how much of this spatial variability is driven by the other variables. We defined a progression of four models, listed in Table S6.

Model	Type 2 Diabetes	Sex	Age	BMI	Household Income	BMI x Household Income
1	■	–	–	–	–	–
2	■	■	■	■	–	–
3	■	■	■	■	■	–
4	■	■	■	■	■	■

Table S6. The four GeoSPM models used for the Birmingham data from UK Biobank.

It is important to keep in mind that unlike in this preliminary analysis, in these GeoSPM models, type 2 diabetes is no longer a response variable but an explanatory or independent variable, which means its effect is marginalised relative to the other variables in each model. By applying a single colour map to all regression coefficient maps across models, the intensity and nature of topological changes—in the marginalised contribution of each variable—become visible, not only within a single model but over the ensemble of four models. Similarly, changes in the extent and location of significant areas, due to the addition of variables as we move from one model to the next, allow us to assess patterns of spatial variability. Lastly, using intersections between significant areas of multiple variables, we can identify areas of significant *conjunctions* between those variables[S2].

S2.3 Kriging

Kriging[S3] is an ensemble of linear least-squares regression techniques for predicting the value of a random field at an unsampled location from observations at other locations. It is commonly used when interpolating spatially-referenced point data over a surface and provides a measure of the

uncertainty in its predictions. In statistics and machine learning, kriging is essentially an application of multivariate Gaussian process prediction. Crucially, kriging requires an explicit model of the spatial covariance and cross-covariance of the data, which needs to be chosen *a priori*. As the random field is generally assumed to be second-order stationary and isotropic, the covariance can be expressed as a function of the Euclidean distance between a pair of points, independently of their actual location in the spatial domain. A theoretical variogram is the quasi-dual form of a covariance model (it is slightly more generic in some situations). An overview of some common theoretical variograms is shown in Figure S6. Parameters required by the selected model are estimated from the data and substituted for the true values when computing the predictions. The covariance and cross-covariance model we used for all kriging predictions presented in the main text is the family of Matérn functions[S4] together with an added "nugget" component. The Matérn model exhibits adaptable smoothness controlled by a parameter κ and is recommended as a sensible default choice in the literature[S5], [S6]. The nugget component adds a discontinuous jump to the covariance function at coincident points and captures variance due to measurement error. Its relative strength is specified by a single numeric parameter. Additional parameters of the Matérn model are the sill, which determines its contribution to the covariance, as well as the range which reflects its spatial scale. For the main results reported in Figures 3 and 4, as well as Figures S7–S11, we left parameter κ fixed at its default gstat setting of 0.5, whereas for the extended comparison of kriging covariance models reported in Sections S3.4, S3.5, S3.6 and S3.7), κ was estimated within a pre-specified range of [0.1, 5].

In cases where the experimental data contained several variables, gstat estimated a linear model of coregionalization (LCM), which expressed all required auto- and cross-covariances as linear combinations of a Matérn function and a nugget component. The range parameter is constrained by gstat to be the same for all covariances in the LCM and was estimated from the first variable in the data prior to estimating the LCM. We configured gstat to use ordinary (co-)kriging with a constant but unknown mean in a global search window.

In addition to the Matérn model, we present a wider comparison of results with the kriging models shown in Figure S6 in Section S3.

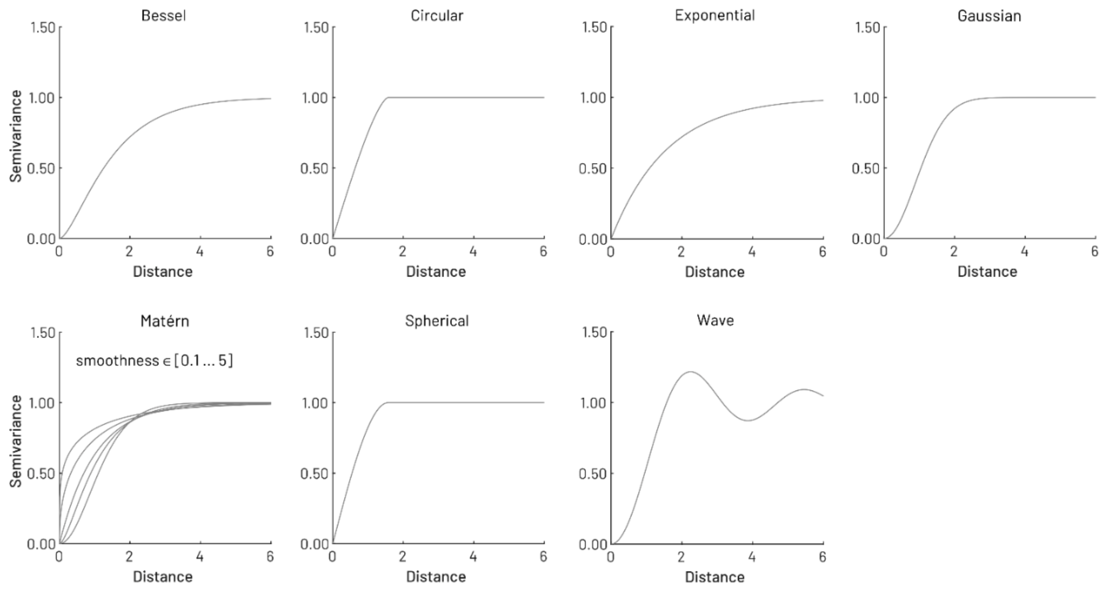

Figure S6. A range of common theoretical variograms to be fitted to the synthetic model data in the kriging experiments.

S3 Additional Results

S3.1 Synthetic Experiment Results for Univariate Models

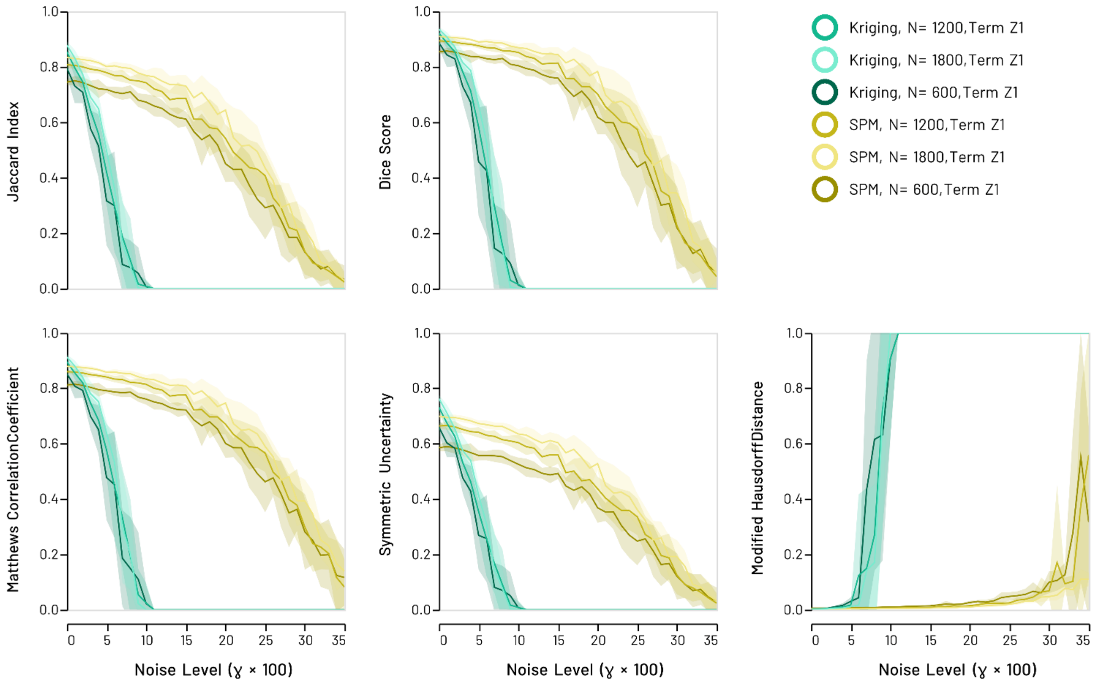

Figure S7. Synthetic univariate snowflake models: Recovery scores for the single GeoSPM and kriging model term in the low (N = 600), middle (N = 1200) and high (N = 1800) sampling regime. Lines denote the mean score across 10 random model realisations, shaded areas its standard deviation to either side of the mean. GeoSPM degrades more slowly and gracefully as noise increases compared with kriging.

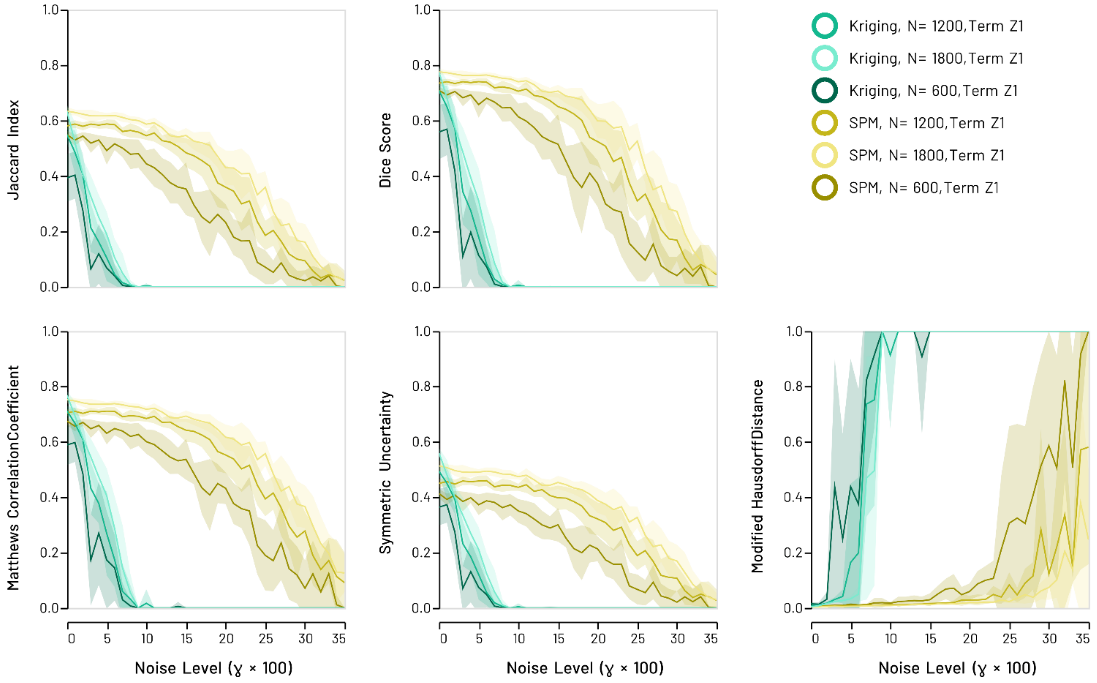

Figure S8. Synthetic univariate anti-snowflake models: Recovery scores for the single GeoSPM and kriging model term in the low (N = 600), middle (N = 1200) and high (N = 1800) sampling regime. Lines denote the mean score across 10 random model realisations, shaded areas its standard deviation to either side of the mean. Areas of overlapping performance are identified by additive shading. GeoSPM degrades more slowly and gracefully as noise increases compared with kriging.

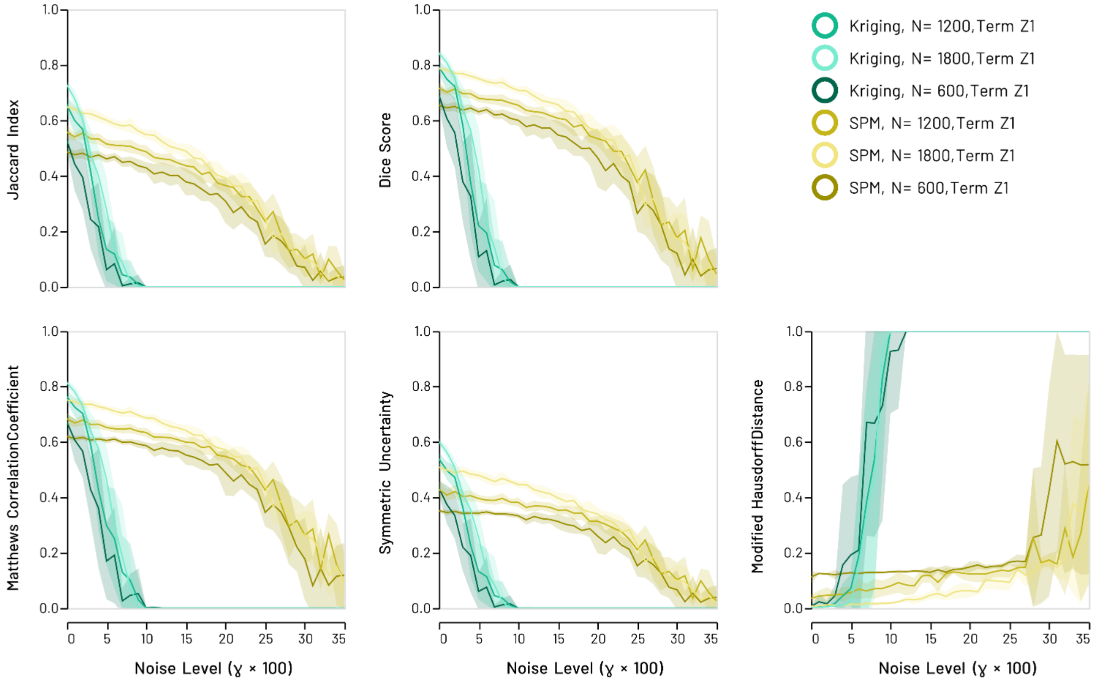

Figure S9. Synthetic univariate snowflake field models: Recovery scores for the single SPM and kriging model term in the low (N = 600), middle (N = 1200) and high (N = 1800) sampling regime. Lines denote the mean score across 10 random model realisations, shaded areas its standard deviation to either side of the mean. Areas of overlapping performance are identified by additive shading. GeoSPM degrades more slowly and gracefully as noise increases compared with kriging.

S3.2 Synthetic Experiment Results for Term $\boldsymbol{Z_2}$ of the Bivariate Models

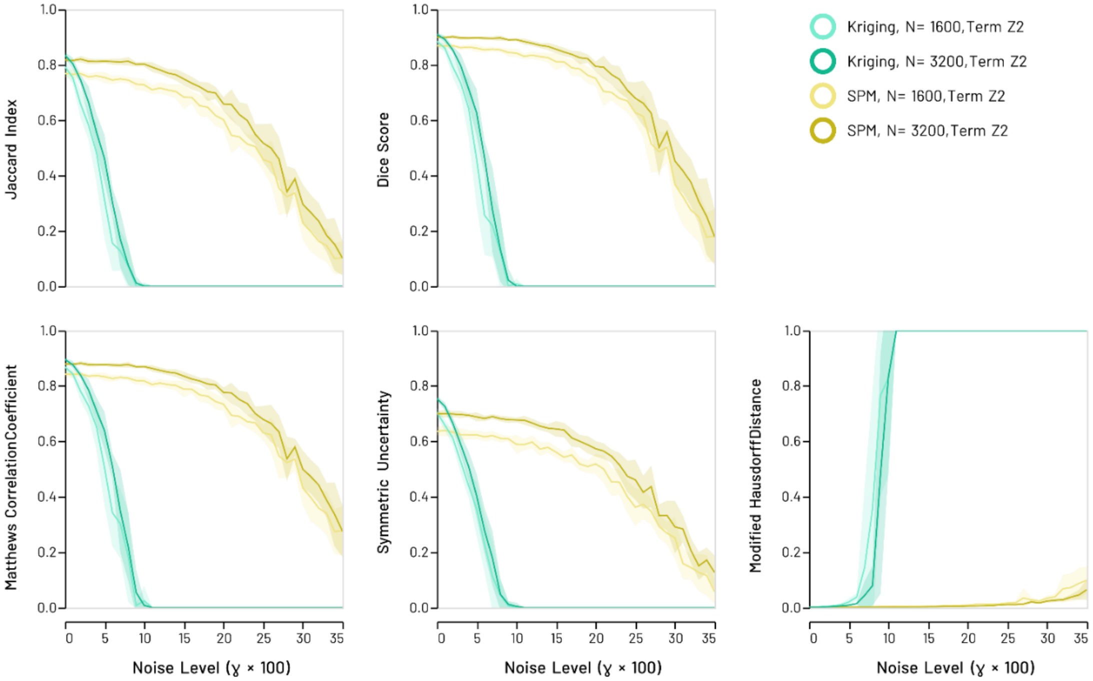

Figure S10. Synthetic snowflake models: Recovery scores for GeoSPM and kriging model term Z_2 in the low (N = 1600) and high (N = 3200) sampling regime. Lines denote the mean score across 10 random model realisations, shaded areas its standard deviation to either side of the mean. Areas of overlapping performance are identified by additive shading. GeoSPM degrades more slowly and gracefully as noise increases compared to kriging.

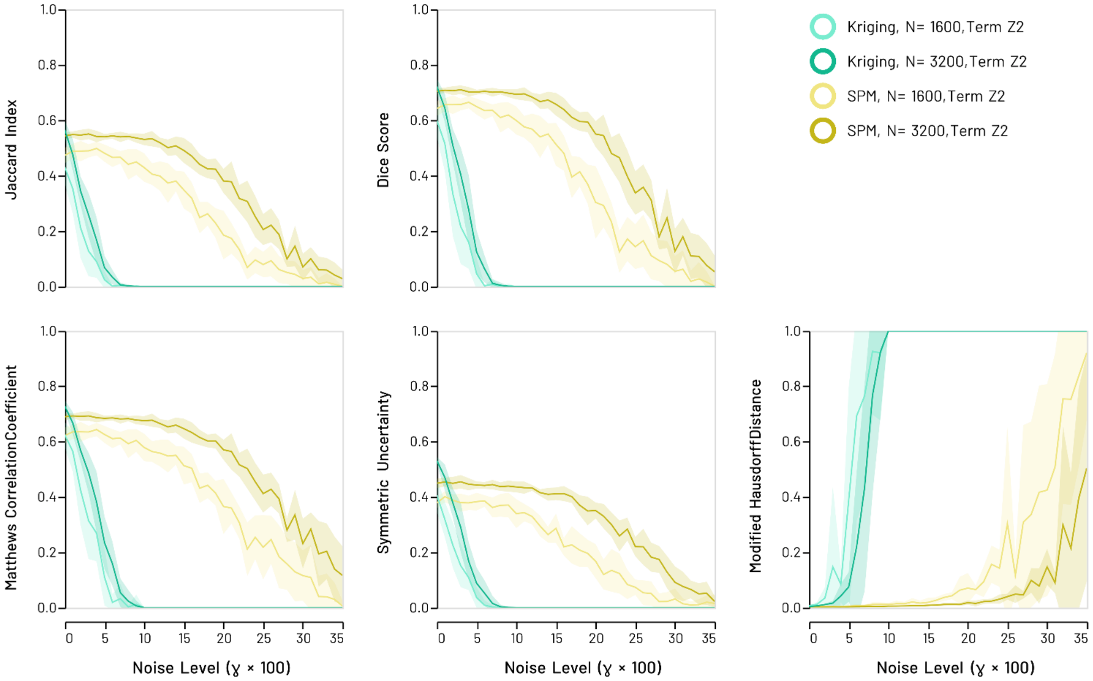

Figure S11. Synthetic anti-snowflake models: Recovery scores for GeoSPM and kriging model term Z_2 in the low (N = 1600) and high (N = 3200) sampling regime. Lines denote the mean score across 10 random model realisations, shaded areas its standard deviation to either side of the mean. Areas of overlapping performance are identified by additive shading. GeoSPM degrades more slowly and gracefully as noise increases compared with kriging.

S3.3 Synthetic Experiment Results for Kriging When Averaging Coincident Observations

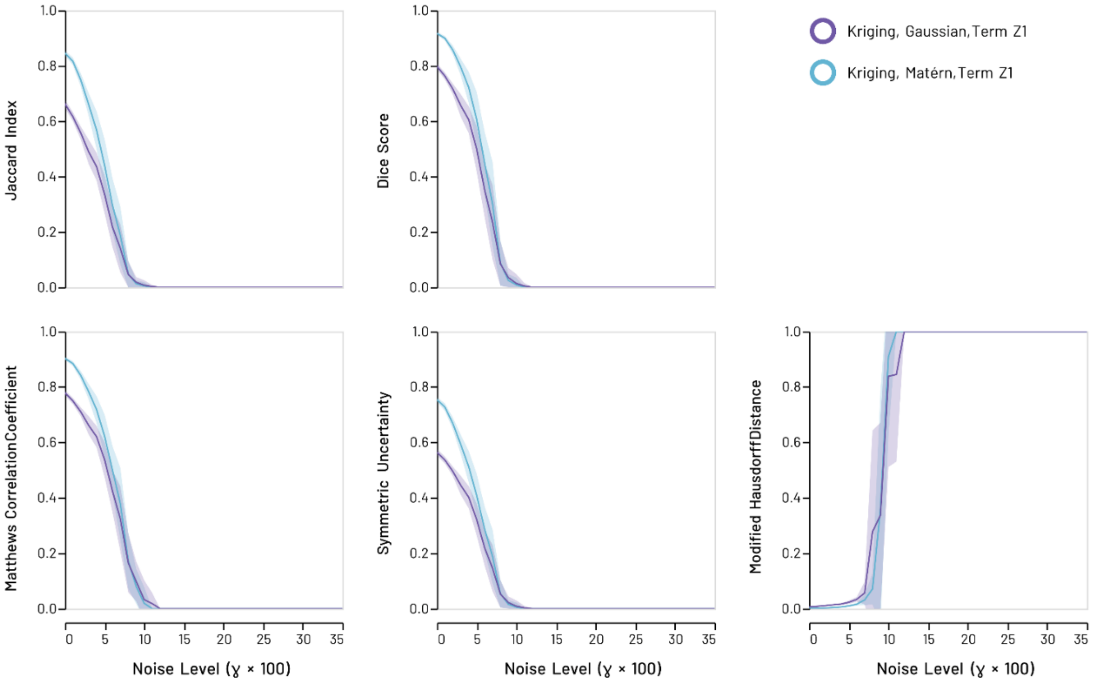

Figure S12. Synthetic bivariate snowflake models: Recovery scores for kriging model term Z_1 with a Matérn covariance function (blue) and a Gaussian covariance function (purple) in the high (N = 3200) sampling regime. Lines denote the mean score across 10 random model realisations, shaded areas its standard deviation to either side of the mean. In both cases coincident observations were *averaged* and reduced to one instead of adding a small amount of random noise to their locations as before. However, this did not change the performance in any meaningful way when compared with a Matérn covariance function with random noise added (as shown in Figure 3): That curve is almost identical to the averaged version displayed here in blue and was therefore left out. The Gaussian covariance function performs slightly worse than the Matérn covariance. This leads us to believe that kriging performance is not improved in our experiments by choosing a different coincident observation regime or covariance function (which is confirmed by the results presented in section S3.4)

S3.4 Extended Synthetic Experiment Results for Term $\boldsymbol{Z_1}$ of the Bivariate Model

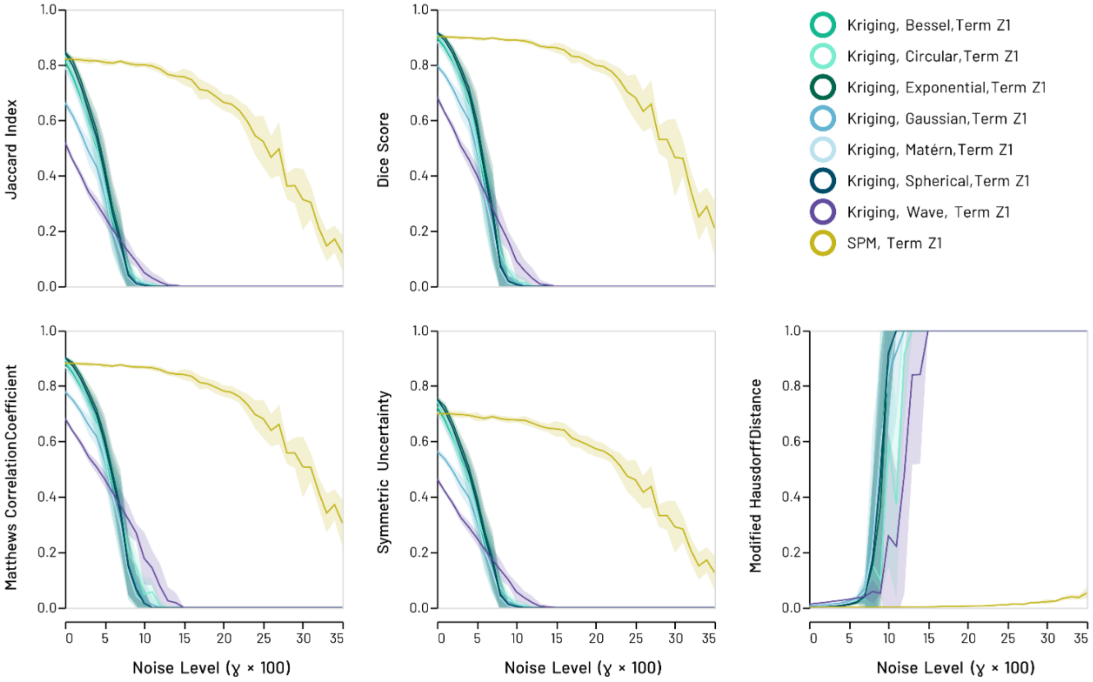

Figure S13. Synthetic snowflake models: Recovery scores for various kriging models *with* a nugget term in comparison with SPM for term Z_1 and the high sampling regime (N = 3200).

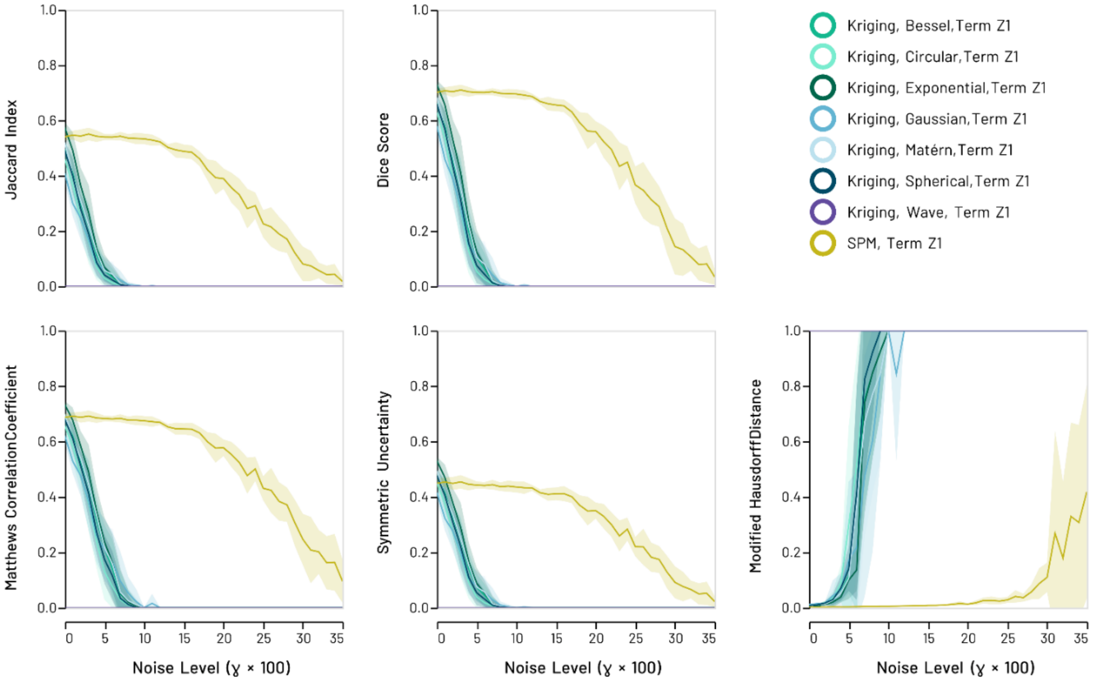

Figure S14. Synthetic anti-snowflake models: Recovery scores for various kriging models *with* a nugget term in comparison with SPM for term Z_1 and the high sampling regime (N = 3200).

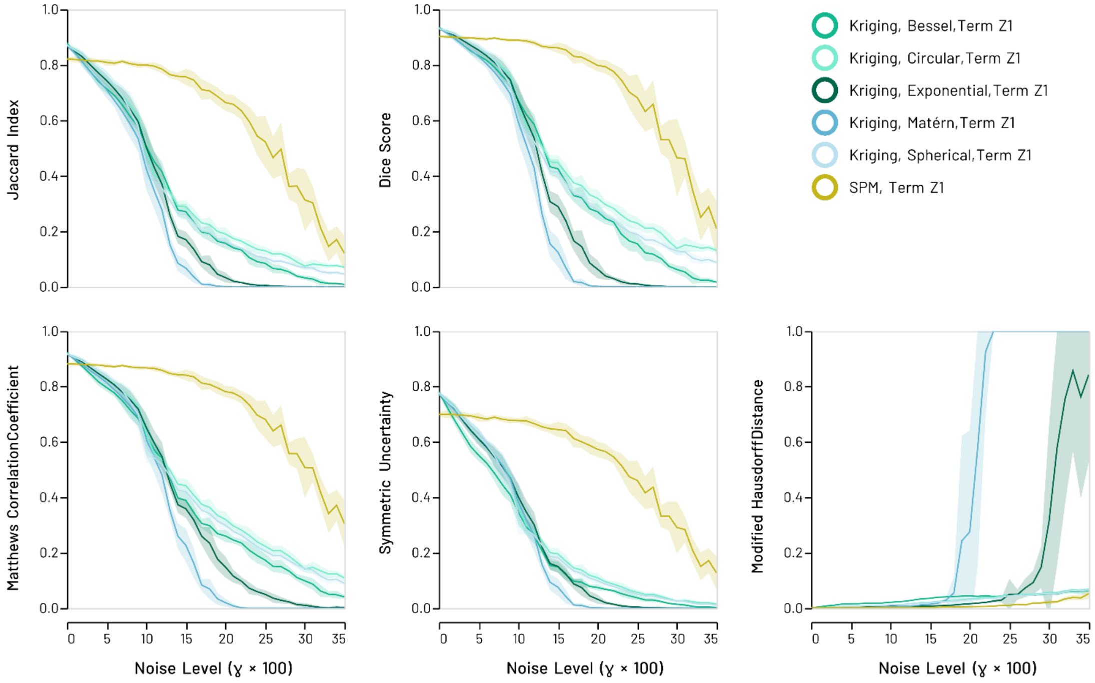

Figure S15. Synthetic snowflake models: Recovery scores for various kriging models *without* a nugget term in comparison with SPM for term Z_1 and the high sampling regime (N = 3200).

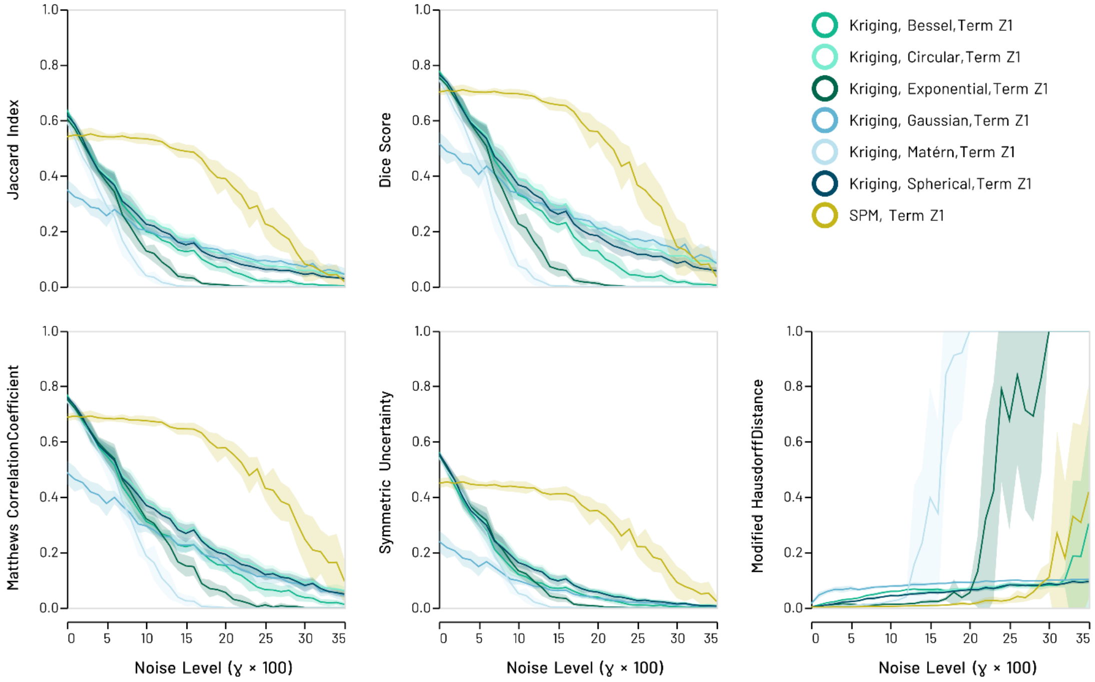

Figure S16. Synthetic anti-snowflake models: Recovery scores for various kriging models *without* a nugget term in comparison with SPM for term Z_1 and the high sampling regime (N = 3200).

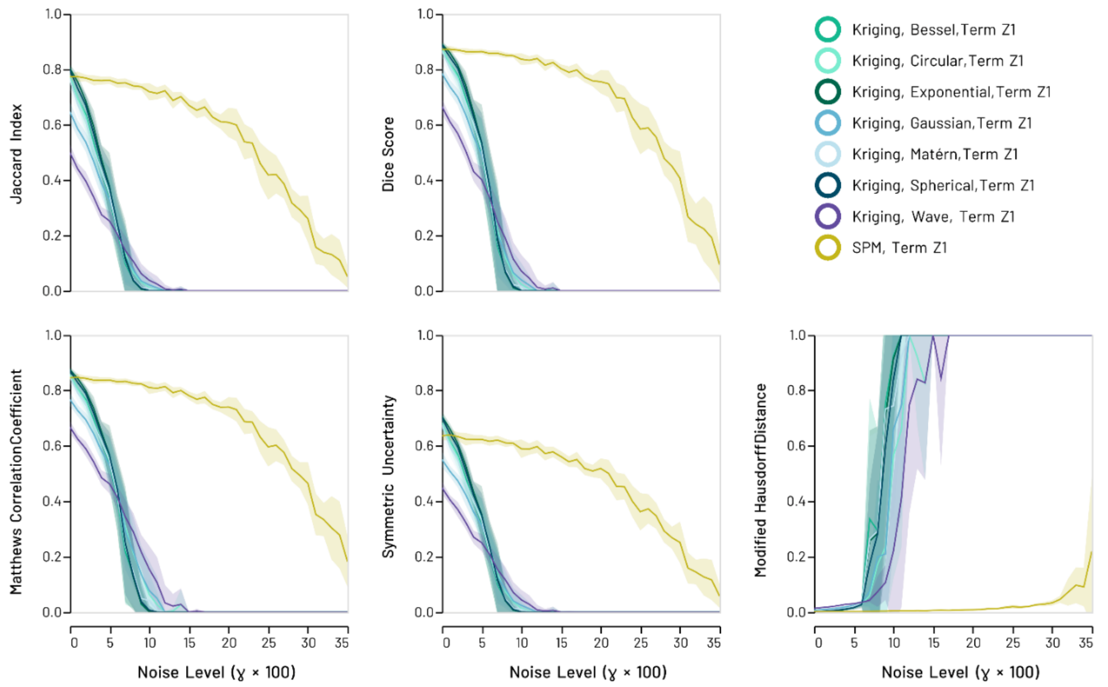

Figure S17. Synthetic snowflake models: Recovery scores for various kriging models *with* a nugget term in comparison with SPM for term Z_1 and the low sampling regime (N = 1600).

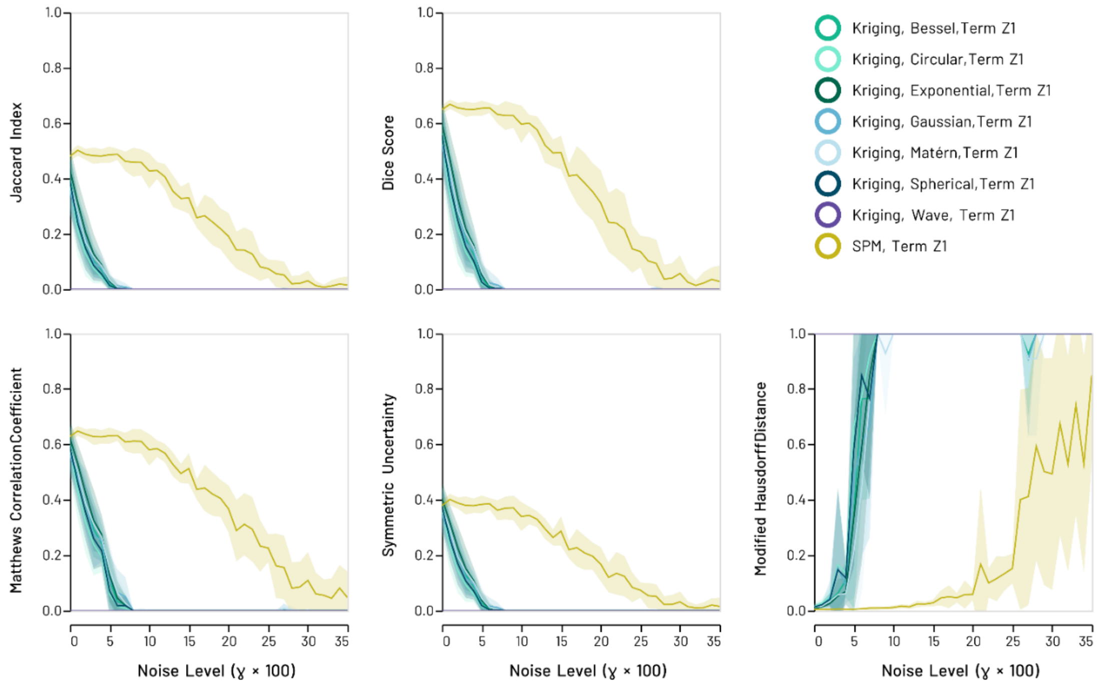

Figure S18. Synthetic anti-snowflake models: Recovery scores for various kriging models *with* a nugget term in comparison with SPM for term Z_1 and the low sampling regime (N = 1600).

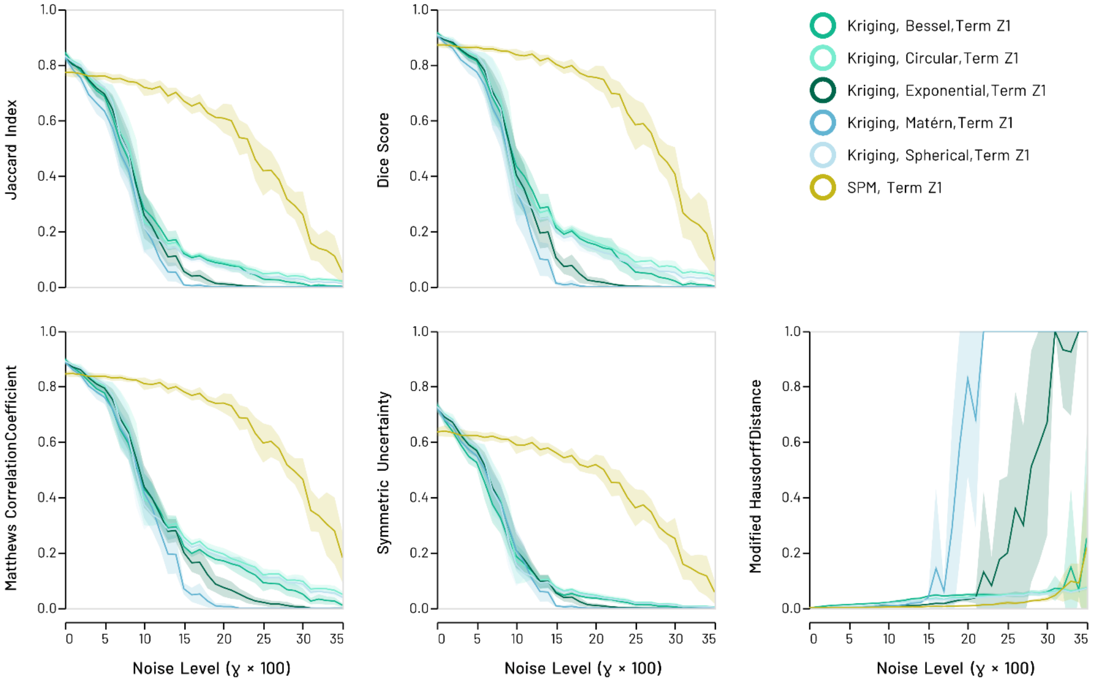

Figure S19. Synthetic snowflake models: Recovery scores for various kriging models *without* a nugget term in comparison with SPM for term Z_1 and the low sampling regime (N = 1600).

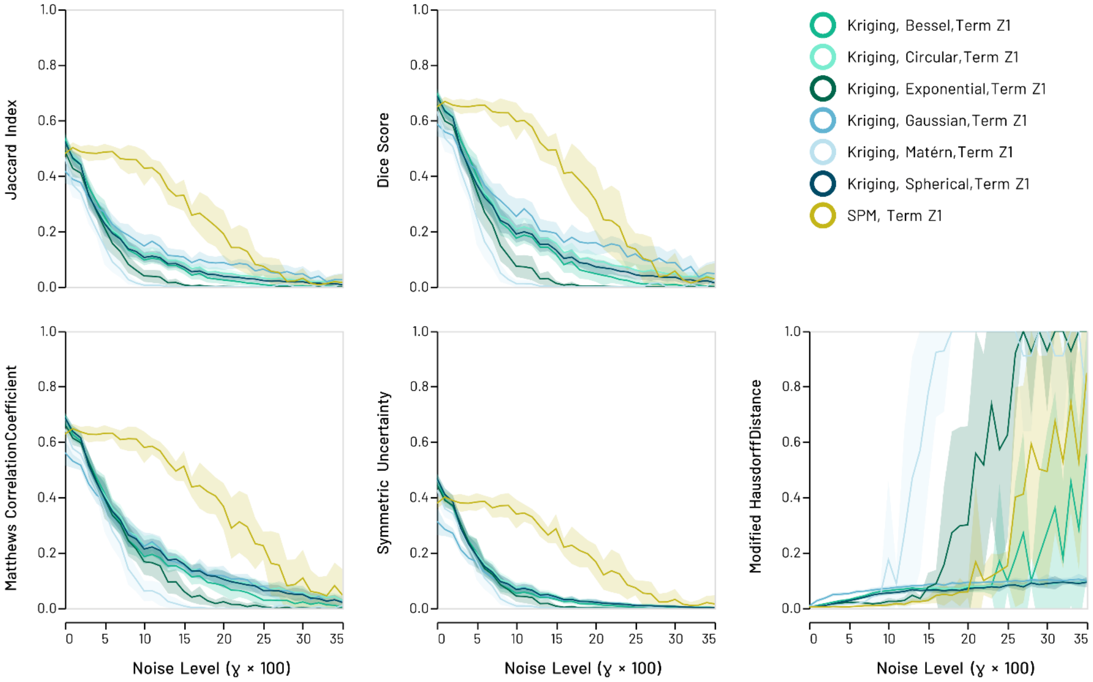

Figure S20. Synthetic anti-snowflake models: Recovery scores for various kriging models *without* a nugget term in comparison with SPM for term Z_1 and the low sampling regime (N = 1600).

S3.5 Extended Synthetic Experiment Kriging Recoveries for Term $\boldsymbol{Z_1}$ of the Bivariate Model

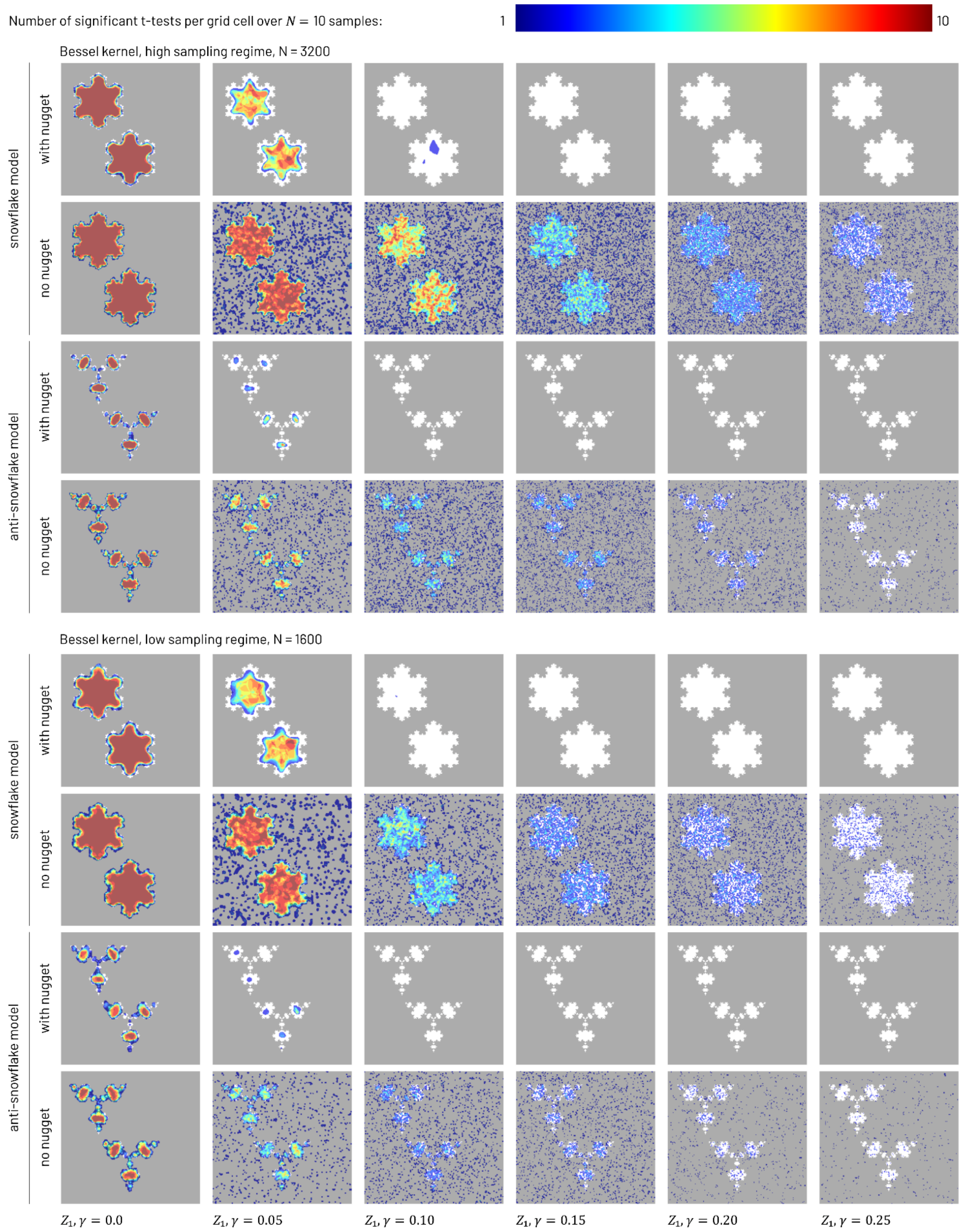

Figure S21. Kriging recoveries of term Z_1 for the Bessel kernel. Columns represent increasing noise levels. Each row shows a combination of the synthetic model used and whether a nugget component was included in the variogram.

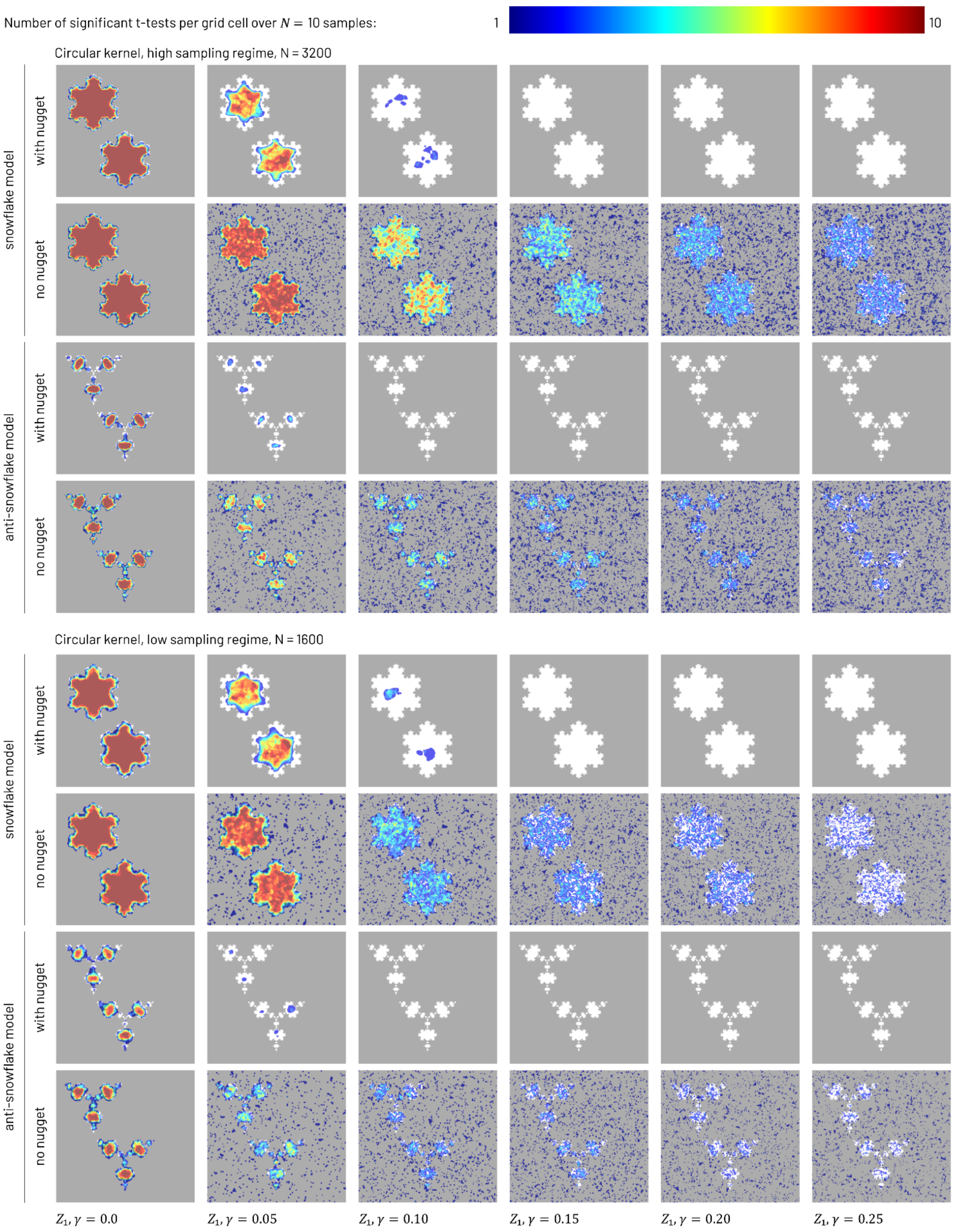

Figure S22. Kriging recoveries of term Z_1 for the circular kernel. Columns represent increasing noise levels. Each row shows a combination of the synthetic model used and whether a nugget component was included in the variogram.

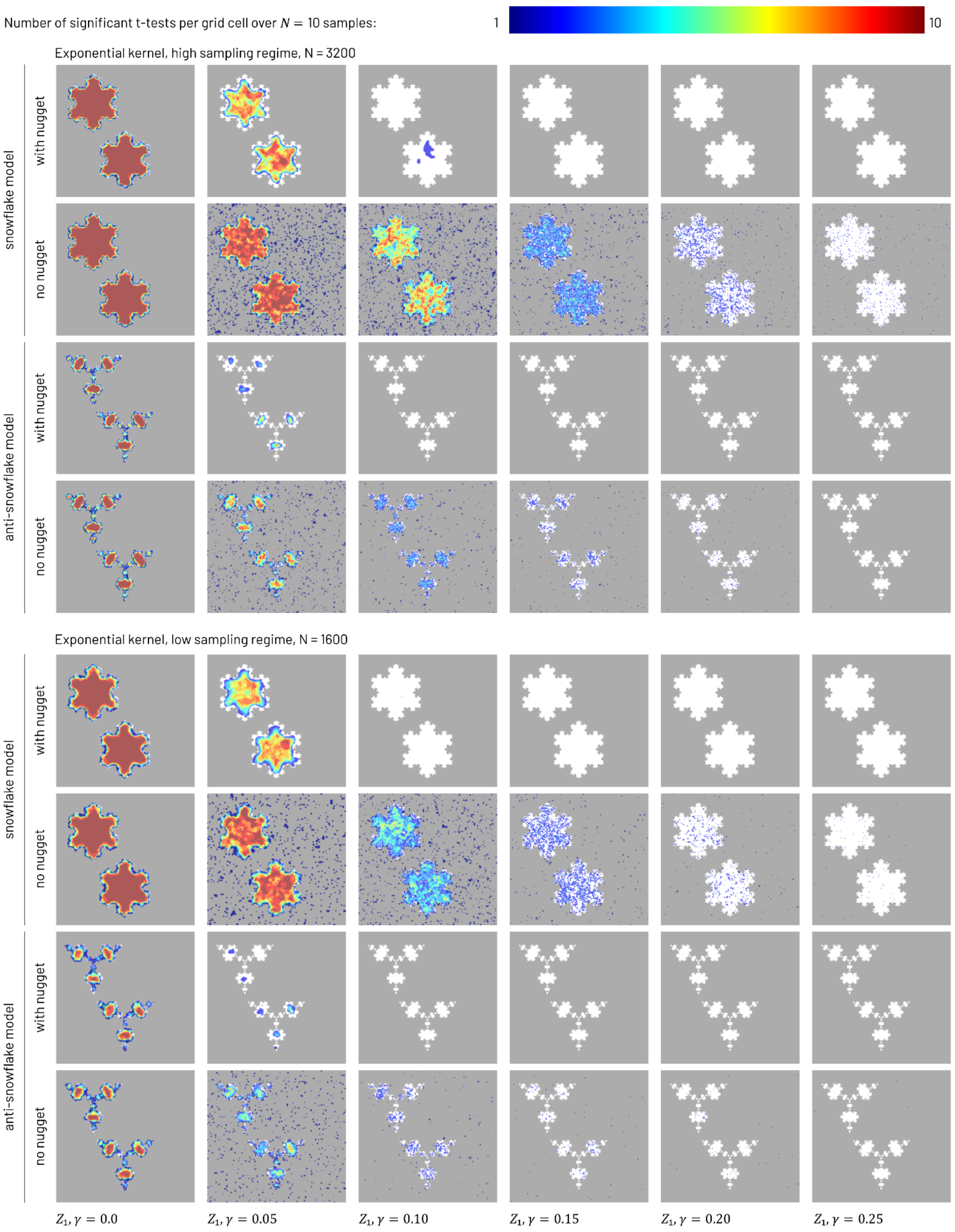

Figure S23. Kriging recoveries of term Z_1 for the exponential kernel. Columns represent increasing noise levels. Each row shows a combination of the synthetic model used and whether a nugget component was included in the variogram.

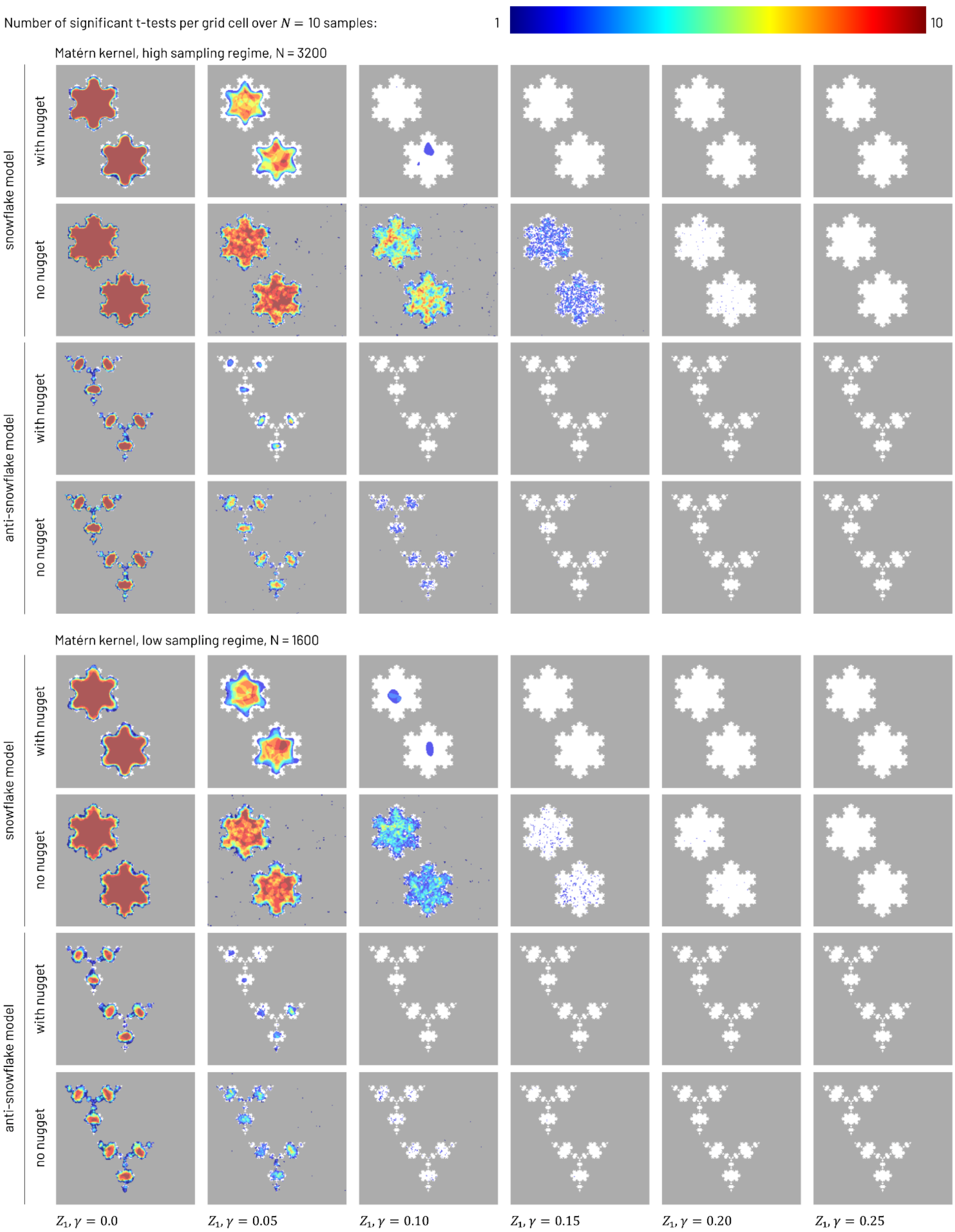

Figure S24. Kriging recoveries of term Z_1 for the Matérn kernel. Columns represent increasing noise levels. Each row shows a combination of the synthetic model used and whether a nugget component was included in the variogram.

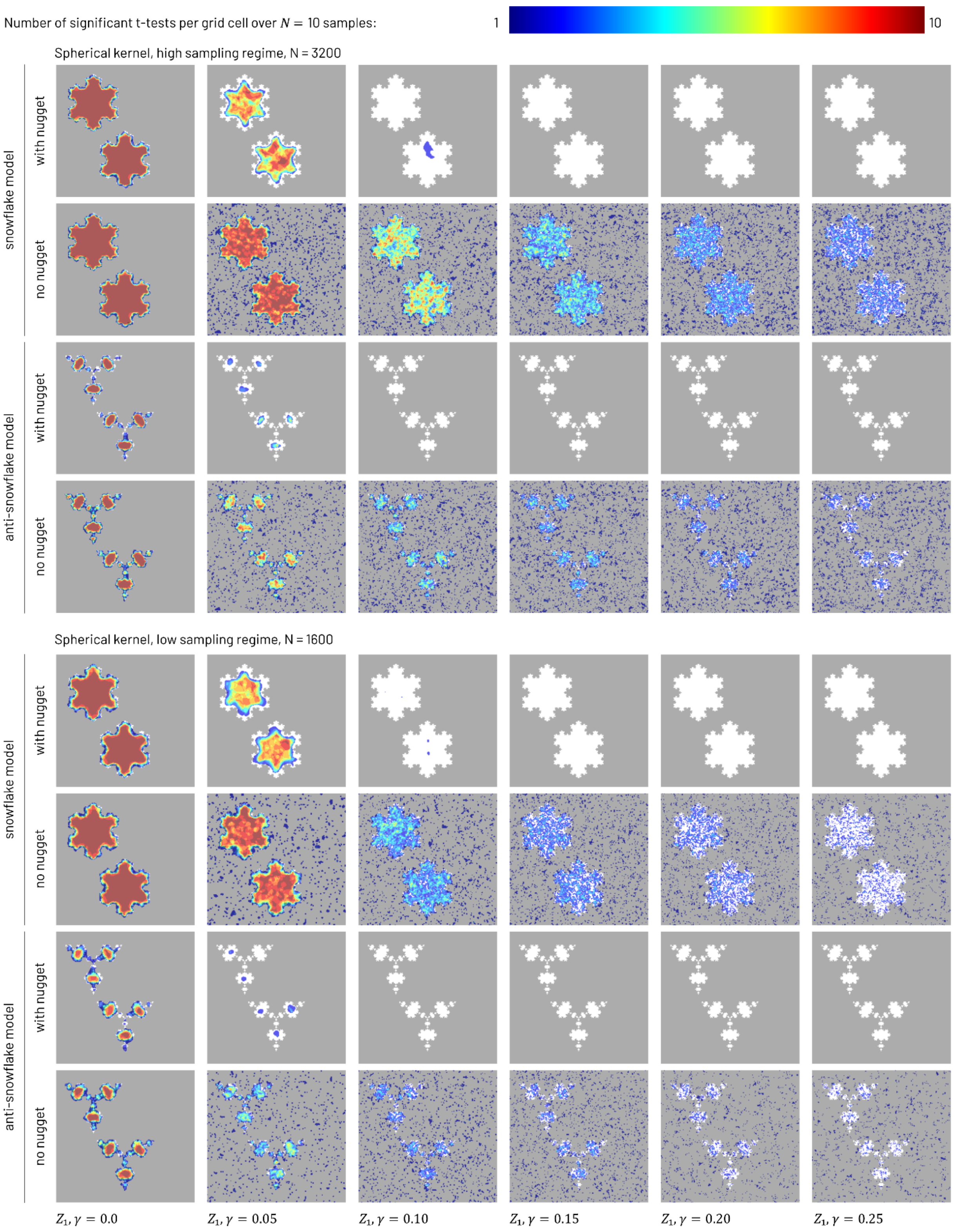

Figure S25. Kriging recoveries of term Z_1 for the spherical kernel. Columns represent increasing noise levels. Each row shows a combination of the synthetic model used and whether a nugget component was included in the variogram.

S3.6 Summary of Kriging Parameters for the Extended Synthetic Experiments

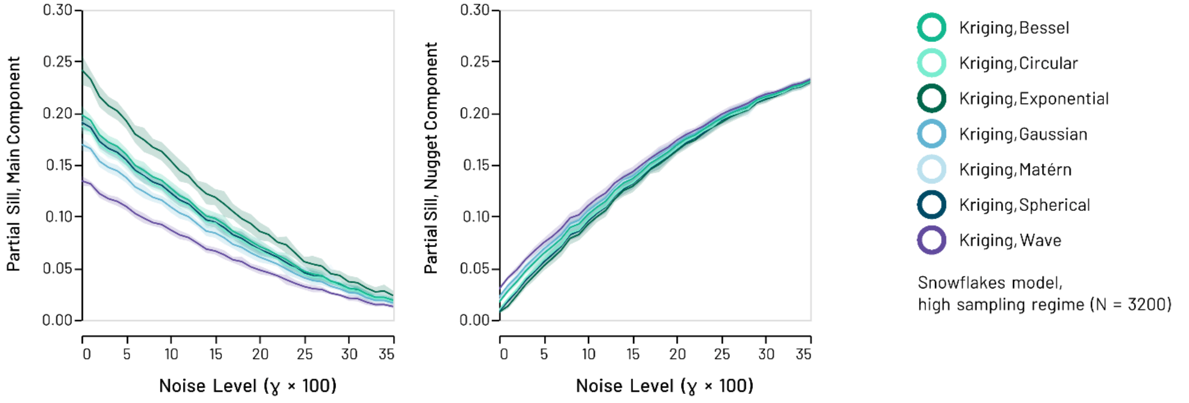

Figure S26. Synthetic snowflakes model: Estimated partial sill parameters for the main (left) and nugget (right) variogram components in the high sampling regime (N = 3200). Lines denote the mean estimate across 10 random model realisations, shaded areas its standard deviation to either side of the mean.

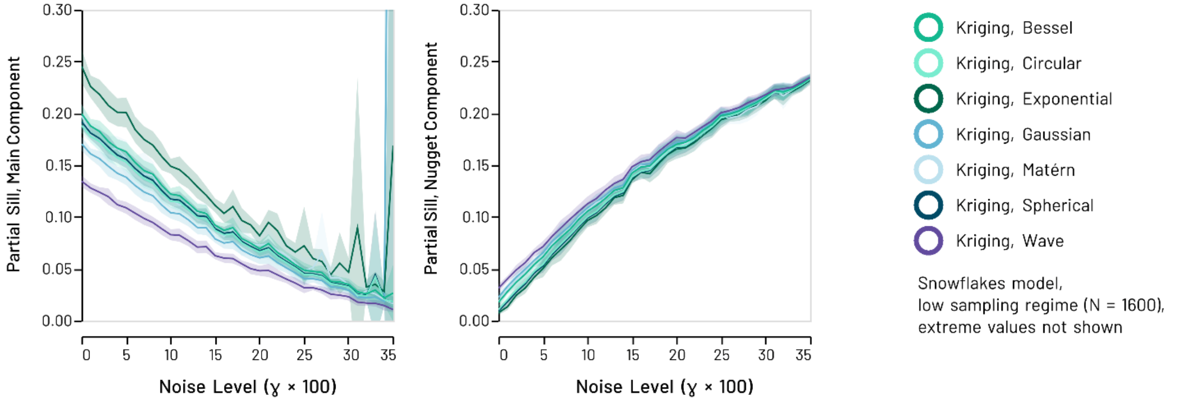

Figure S27. Synthetic snowflakes model: Estimated partial sill parameters for the main (left) and nugget (right) variogram components in the low sampling regime (N = 1600). Lines denote the mean estimate across 10 random model realisations, shaded areas its standard deviation to either side of the mean. At high levels of noise, estimates for some main components become unreliable, resulting in extreme values.

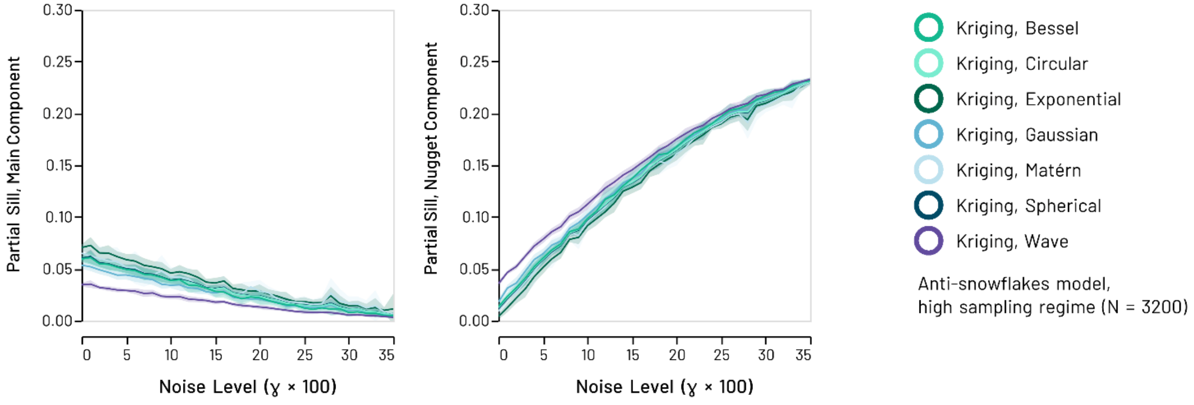

Figure S28. Synthetic anti-snowflakes model: Estimated partial sill parameters for the main (left) and nugget (right) variogram components in the high sampling regime (N = 3200). Lines denote the mean estimate across 10 random model realisations, shaded areas its standard deviation to either side of the mean.

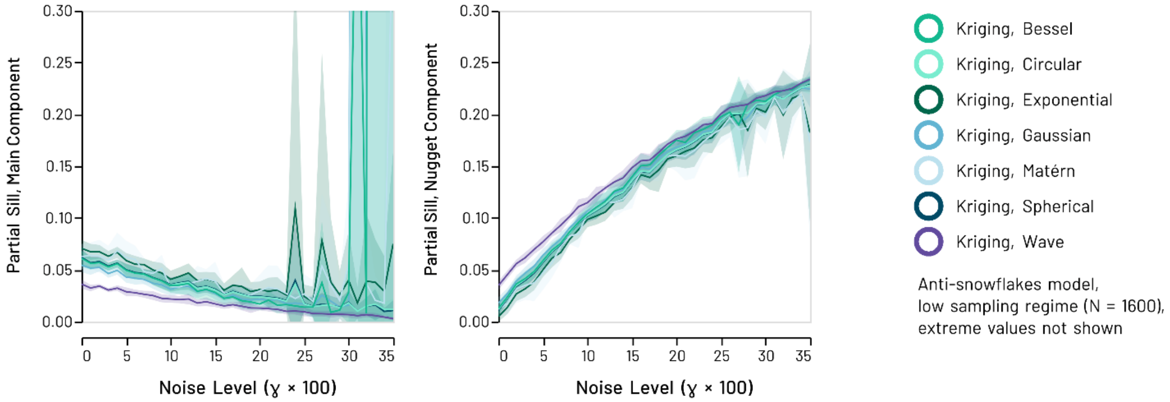

Figure S29. Synthetic anti-snowflakes model: Estimated partial sill parameters for the main (left) and nugget (right) variogram components in the low sampling regime (N = 1600). Lines denote the mean estimate across 10 random model realisations, shaded areas its standard deviation to either side of the mean. At high levels of noise, estimates for some main components become unreliable, resulting in extreme values.

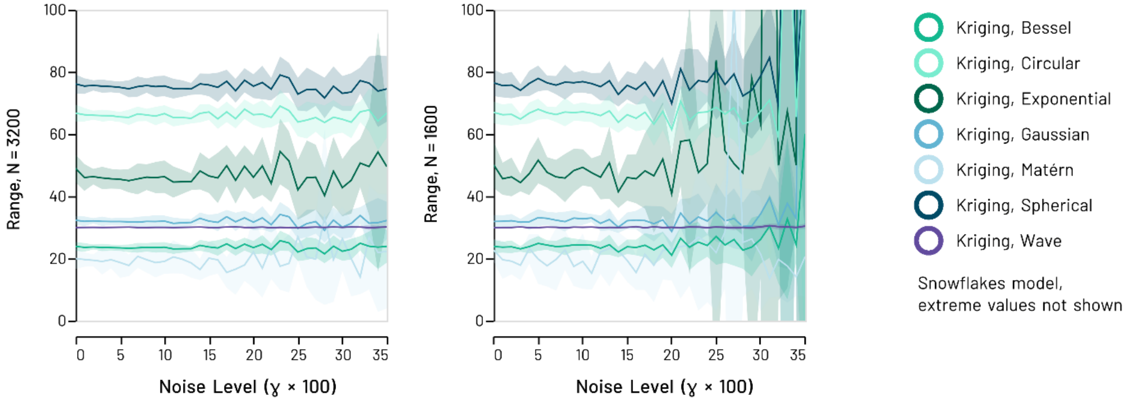

Figure S30. Synthetic snowflakes model: Estimated range parameters for the main variogram component in the high sampling regime (N = 3200, left) and low sampling regime (N = 1600, right). Lines denote the mean estimate across 10 random model realisations, shaded areas its standard deviation to either side of the mean. At high levels of noise, estimates unreliable, resulting in extreme values.

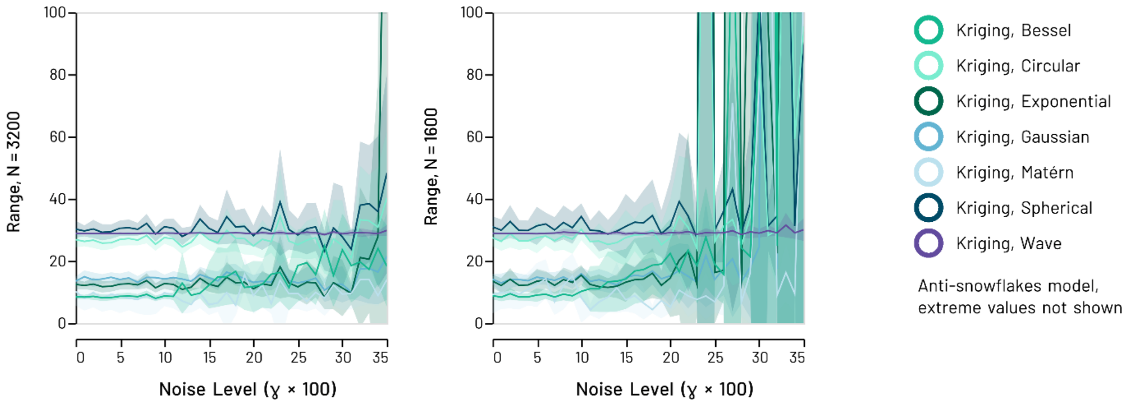

Figure S31. Synthetic anti-snowflakes model: Estimated range parameters for the main variogram component in the high sampling regime (N = 3200, left) and low sampling regime (N = 1600, right). Lines denote the mean estimate across 10 random model realisations, shaded areas its standard deviation to either side of the mean. At high levels of noise, estimates unreliable, resulting in extreme values.

S3.7 Summary of Kriging Variograms for the Extended Synthetic Experiments
S3.7.1 Bessel Kernel

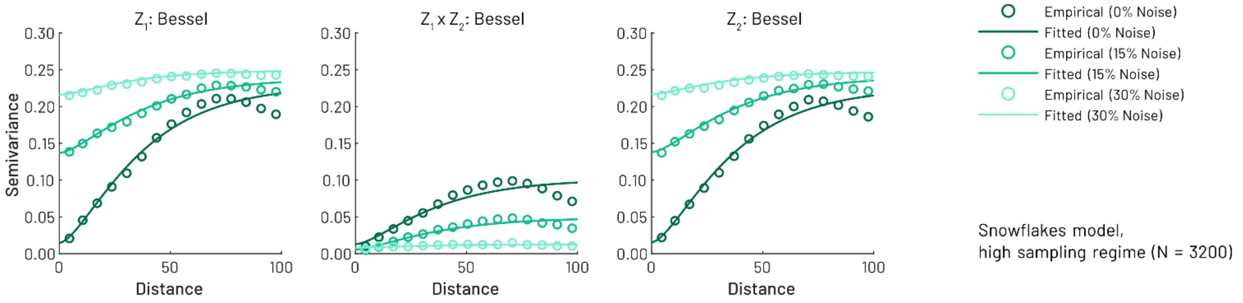

Figure S32. Synthetic snowflakes model: Empirical semivariograms and fitted Bessel kernel *with* a nugget component at three different noise levels for terms Z_1 and Z_2 and their cross-covariance in the high sampling regime (N = 3200) for a single run of the experiments.

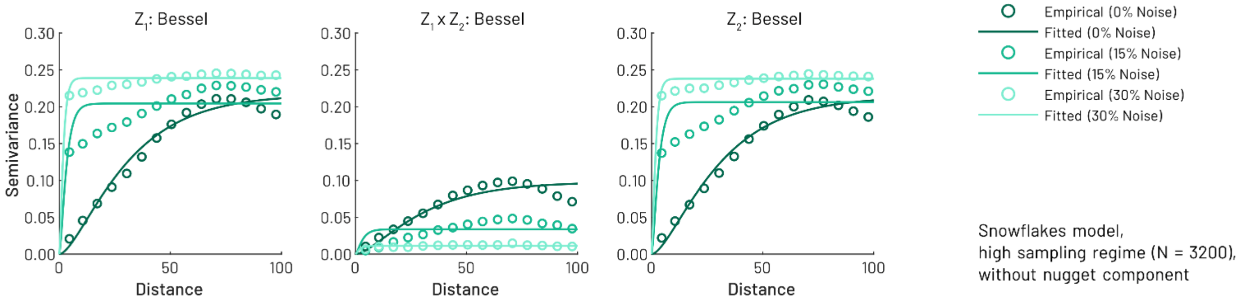

Figure S33. Synthetic snowflakes model: Empirical semivariograms and fitted Bessel kernel *without* a nugget component at three different noise levels for terms Z_1 and Z_2 and their cross-covariance in the high sampling regime (N = 3200) for a single run.

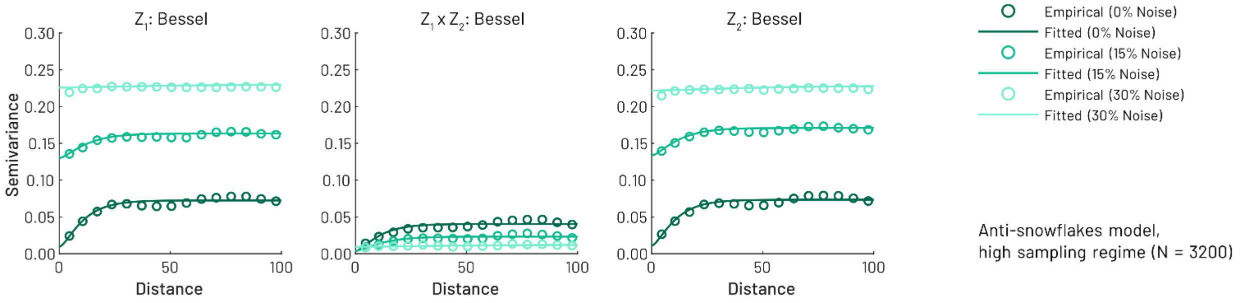

Figure S34. Synthetic anti-snowflakes model: Empirical semivariograms and fitted Bessel kernel *with* a nugget component at three different noise levels for terms Z_1 and Z_2 and their cross-covariance in the high sampling regime (N = 3200) for a single run.

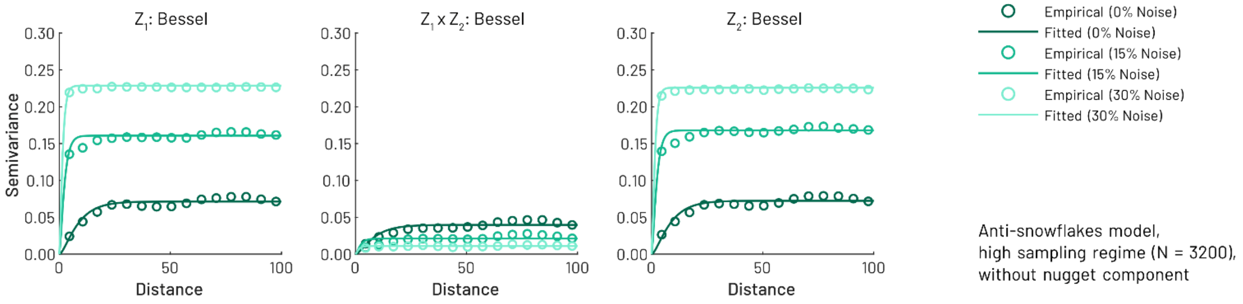

Figure S35. Synthetic anti-snowflakes model: Empirical semivariograms and fitted Bessel kernel *without* a nugget component at three different noise levels for terms Z_1 and Z_2 and their cross-covariance in the high sampling regime (N = 3200) for a single run.

S3.7.2 Circular Kernel

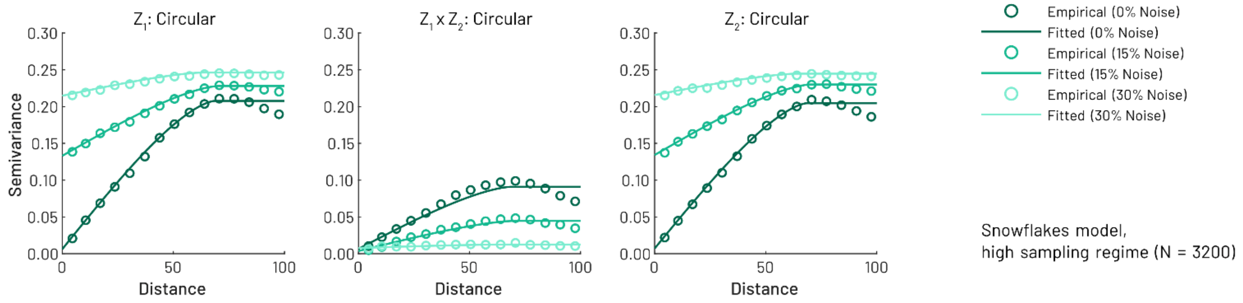

Figure S36. Synthetic snowflakes model: Empirical semivariograms and fitted circular kernel *with* a nugget component at three different noise levels for terms Z_1 and Z_2 and their cross-covariance in the high sampling regime (N = 3200) for a single run.

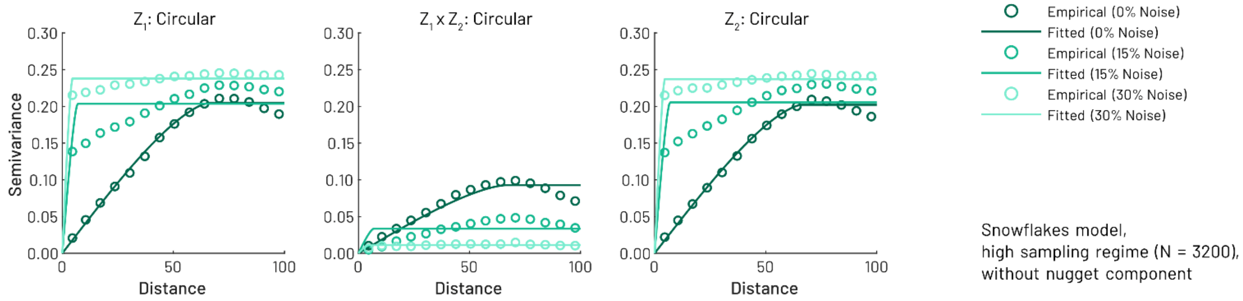

Figure S37. Synthetic snowflakes model: Empirical semivariograms and fitted circular kernel *without* a nugget component at three different noise levels for terms Z_1 and Z_2 and their cross-covariance in the high sampling regime (N = 3200) for a single run.

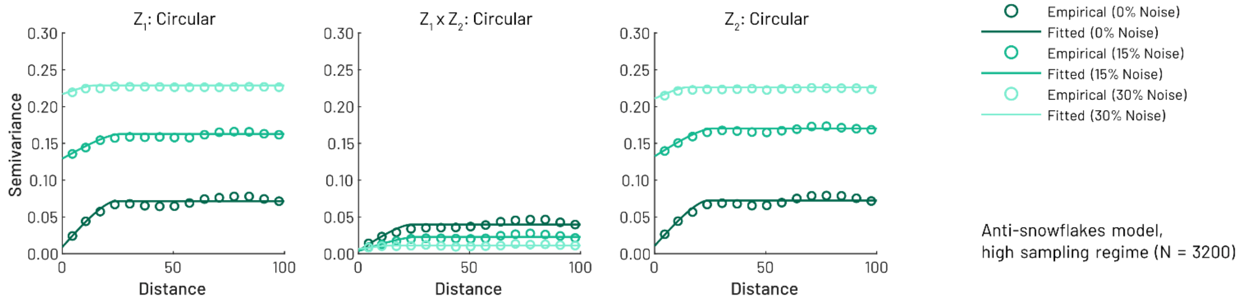

Figure S38. Synthetic anti-snowflakes model: Empirical semivariograms and fitted circular kernel *with* a nugget component at three different noise levels for terms Z_1 and Z_2 and their cross-covariance in the high sampling regime (N = 3200) for a single run.

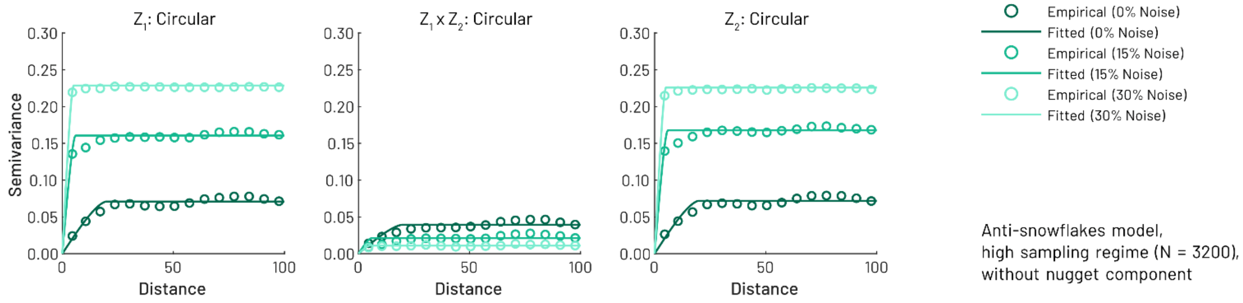

Figure S39. Synthetic anti-snowflakes model: Empirical semivariograms and fitted circular kernel *without* a nugget component at three different noise levels for terms Z_1 and Z_2 and their cross-covariance in the high sampling regime (N = 3200) for a single run.

S3.7.3 Exponential Kernel

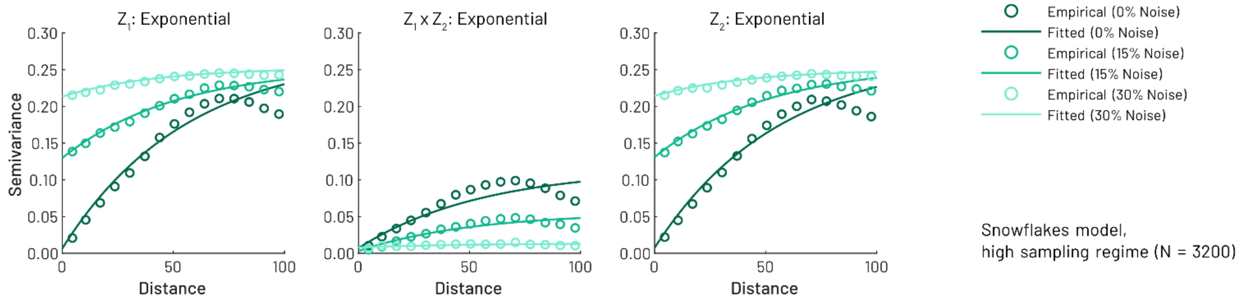

Figure S40. Synthetic snowflakes model: Empirical semivariograms and fitted exponential kernel *with* a nugget component at three different noise levels for terms Z_1 and Z_2 and their cross-covariance in the high sampling regime (N = 3200) for a single run.

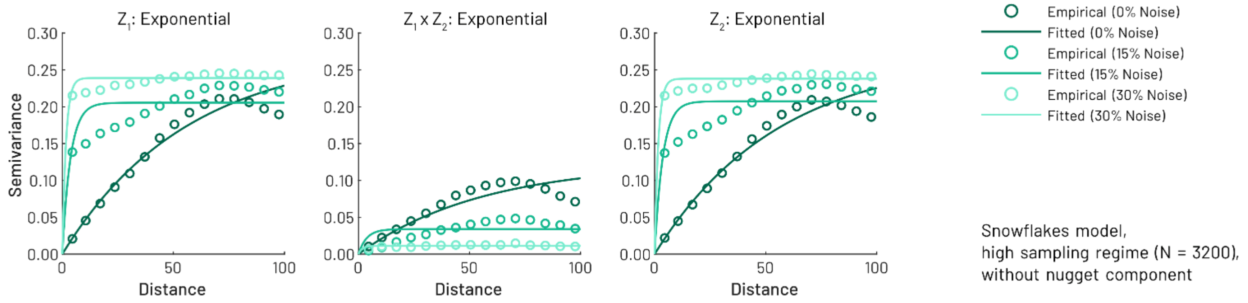

Figure S41. Synthetic snowflakes model: Empirical semivariograms and fitted exponential kernel *without* a nugget component at three different noise levels for terms Z_1 and Z_2 and their cross-covariance in the high sampling regime (N = 3200) for a single run.

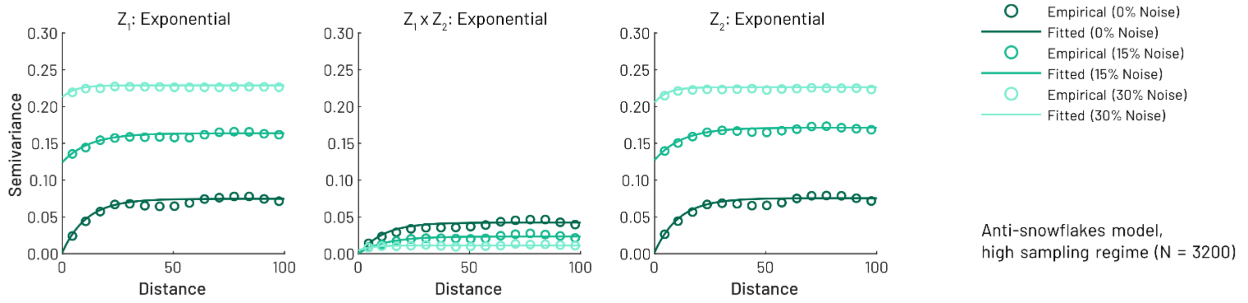

Figure S42. Synthetic anti-snowflakes model: Empirical semivariograms and fitted exponential kernel *with* a nugget component at three different noise levels for terms Z_1 and Z_2 and their cross-covariance in the high sampling regime (N = 3200) for a single run.

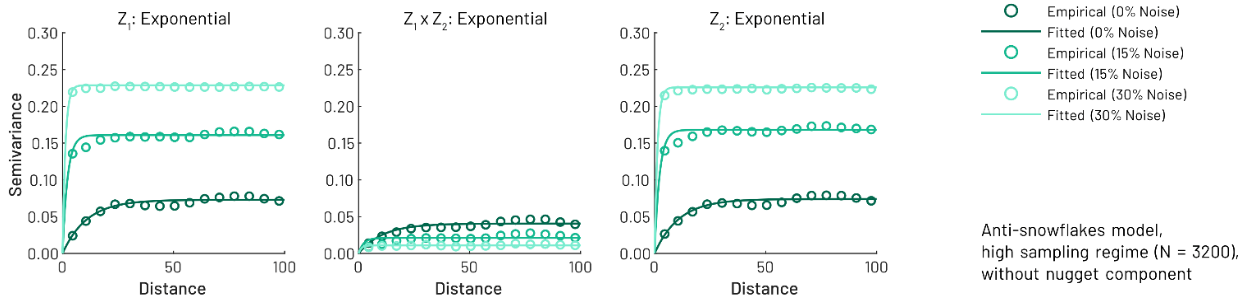

Figure S43. Synthetic anti-snowflakes model: Empirical semivariograms and fitted exponential kernel *without* a nugget component at three different noise levels for terms Z_1 and Z_2 and their cross-covariance in the high sampling regime (N = 3200) for a single run.

S3.7.4 Gaussian Kernel

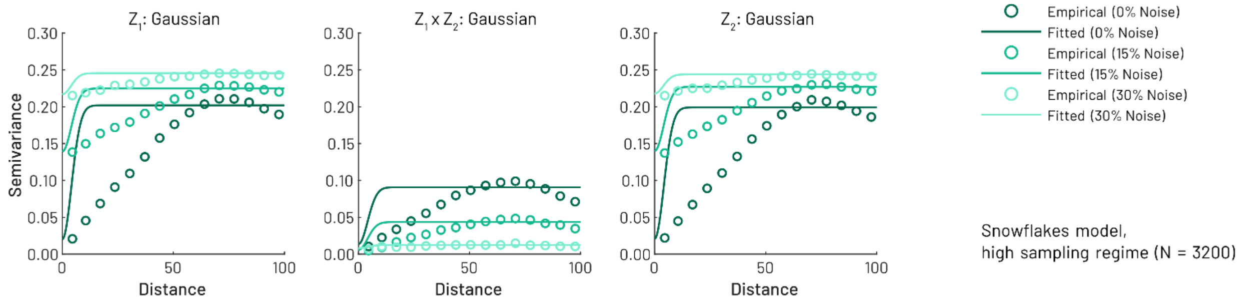

Figure S44. Synthetic snowflakes model: Empirical semivariograms and fitted Gaussian kernel *with* a nugget component at three different noise levels for terms Z_1 and Z_2 and their cross-covariance in the high sampling regime (N = 3200) for a single run.

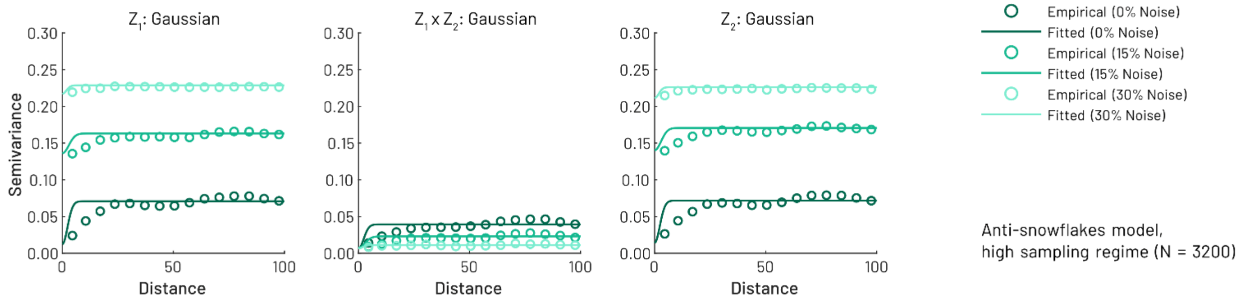

Figure S45. Synthetic anti-snowflakes model: Empirical semivariograms and fitted Gaussian kernel *with* a nugget component at three different noise levels for terms Z_1 and Z_2 and their cross-covariance in the high sampling regime (N = 3200) for a single run.

S3.7.5 Matérn Kernel

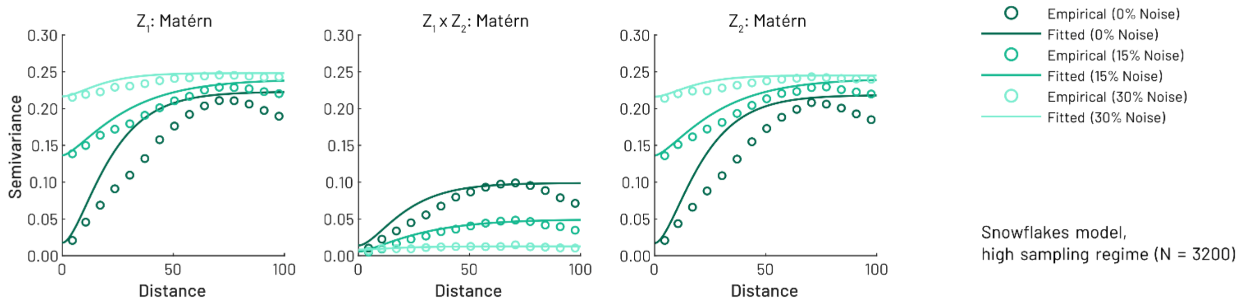

Figure S46. Synthetic snowflakes model: Empirical semivariograms and fitted Matérn kernel *with* a nugget component at three different noise levels for terms Z_1 and Z_2 and their cross-covariance in the high sampling regime (N = 3200) for a single run.

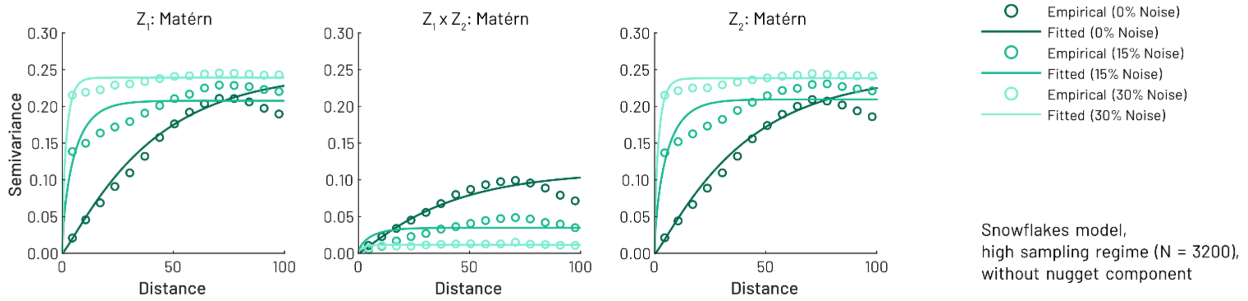

Figure S47. Synthetic snowflakes model: Empirical semivariograms and fitted Matérn kernel *without* a nugget component at three different noise levels for terms Z_1 and Z_2 and their cross-covariance in the high sampling regime (N = 3200) for a single run.

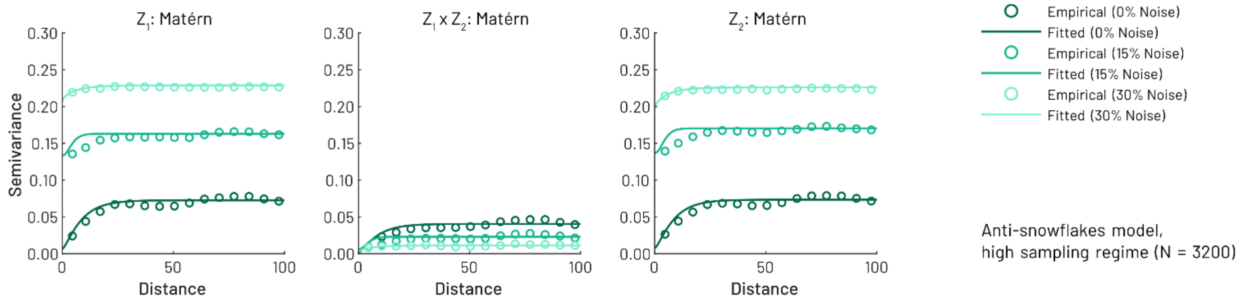

Figure S48. Synthetic anti-snowflakes model: Empirical semivariograms and fitted Matérn kernel *with* a nugget component at three different noise levels for terms Z_1 and Z_2 and their cross-covariance in the high sampling regime (N = 3200) for a single run.

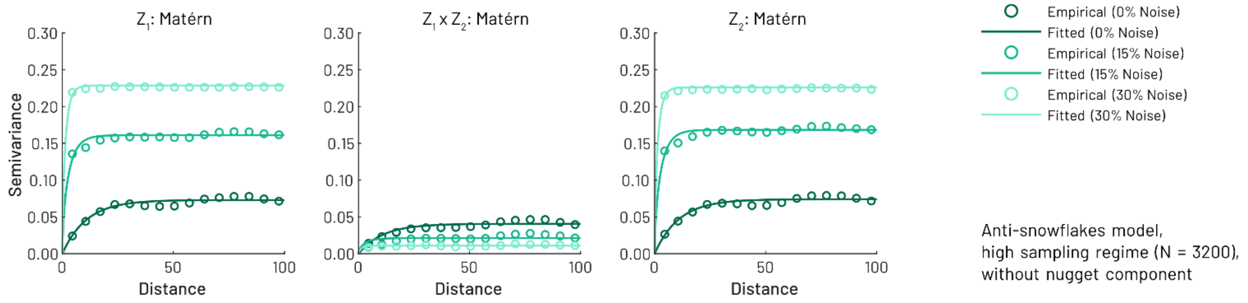

Figure S49. Synthetic anti-snowflakes model: Empirical semivariograms and fitted Matérn kernel *without* a nugget component at three different noise levels for terms Z_1 and Z_2 and their cross-covariance in the high sampling regime (N = 3200) for a single run.

S3.7.6 Spherical Kernel

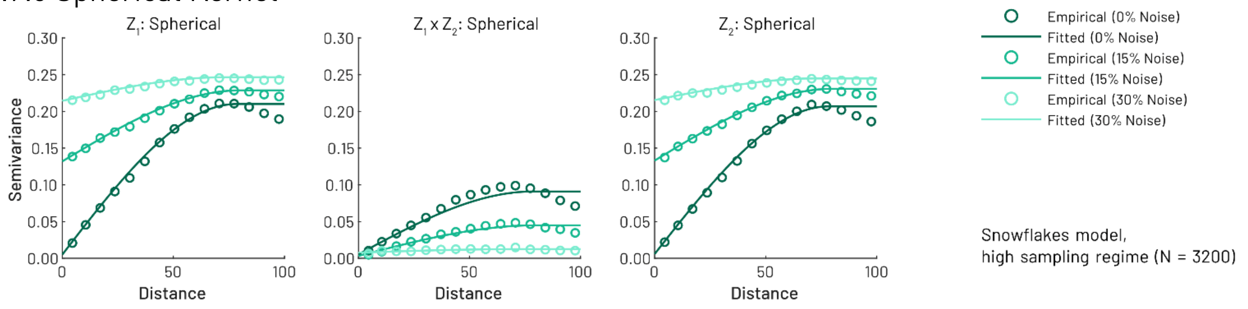

Figure S50. Synthetic snowflakes model: Empirical semivariograms and fitted spherical kernel *with* a nugget component at three different noise levels for terms Z_1 and Z_2 and their cross-covariance in the high sampling regime (N = 3200) for a single run.

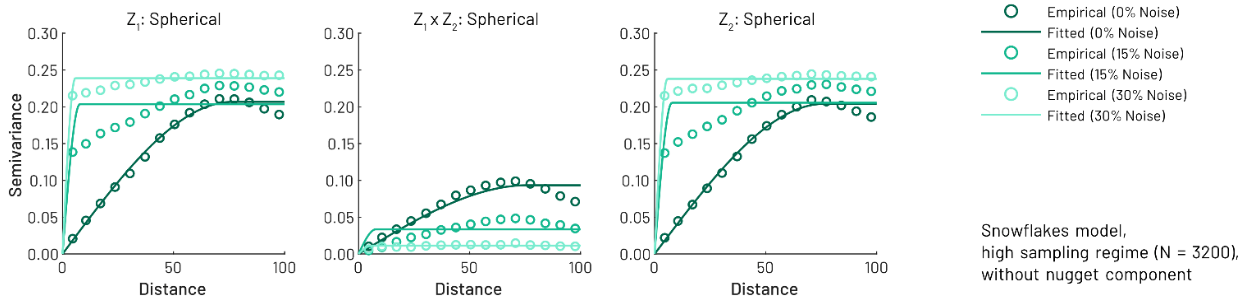

Figure S51. Synthetic snowflakes model: Empirical semivariograms and fitted spherical kernel *without* a nugget component at three different noise levels for terms Z_1 and Z_2 and their cross-covariance in the high sampling regime (N = 3200) for a single run.

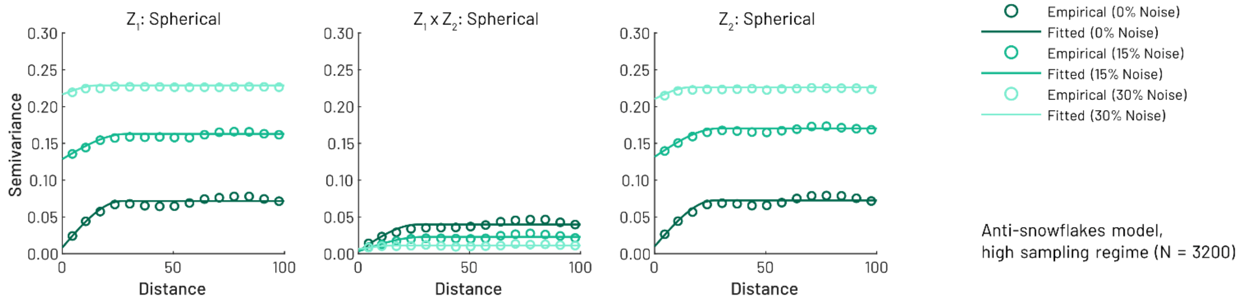

Figure S52. Synthetic anti-snowflakes model: Empirical semivariograms and fitted spherical kernel *with* a nugget component at three different noise levels for terms Z_1 and Z_2 and their cross-covariance in the high sampling regime (N = 3200) for a single run.

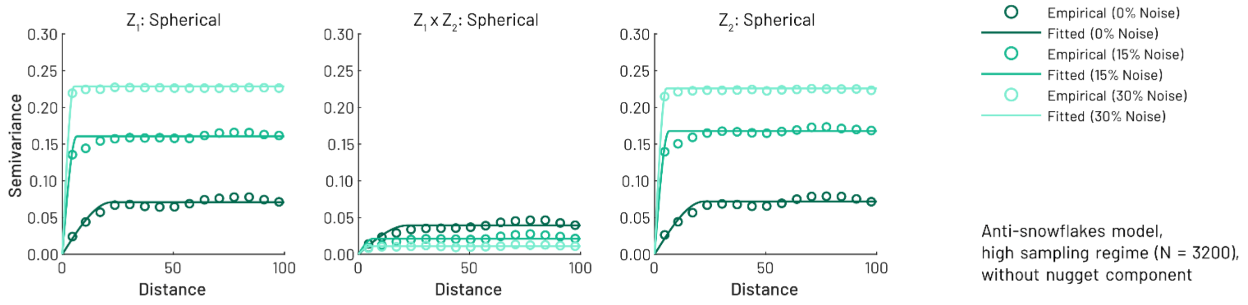

Figure S53. Synthetic anti-snowflakes model: Empirical semivariograms and fitted spherical kernel *without* a nugget component at three different noise levels for terms Z_1 and Z_2 and their cross-covariance in the high sampling regime (N = 3200) for a single run.

S3.7.7 Wave Kernel

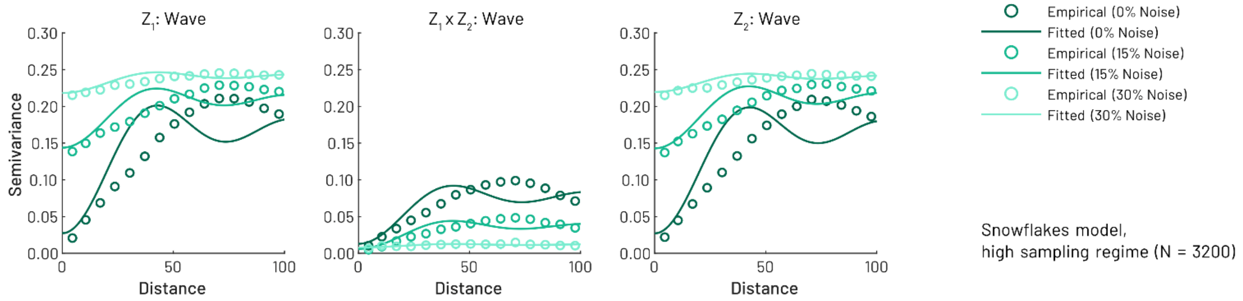

Figure S54. Synthetic snowflakes model: Empirical semivariograms and fitted wave kernel *with* a nugget component at three different noise levels for terms Z_1 and Z_2 and their cross-covariance in the high sampling regime (N = 3200) for a single run.

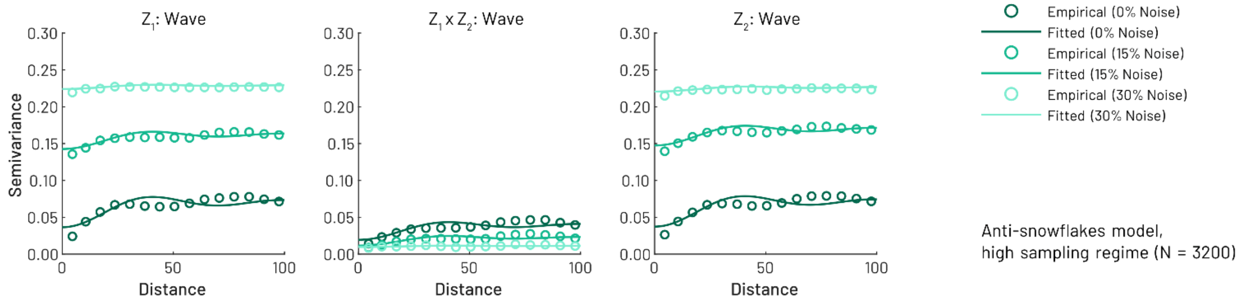

Figure S55. Synthetic anti-snowflakes model: Empirical semivariograms and fitted wave kernel *with* a nugget component at three different noise levels for terms Z_1 and Z_2 and their cross-covariance in the high sampling regime (N = 3200) for a single run.

ICD-9 codes

ICD-9 Group	Description	ICD-9 Codes in Group
250.0	Diabetes mellitus without mention of complication	250.00, 250.02
250.1	Diabetes with ketoacidosis	250.10, 250.12
250.2	Diabetes with hyperosmolarity	250.20, 250.22
250.3	Diabetes with other coma	250.30, 250.32
250.4	Diabetes with renal manifestations	250.40, 250.42
250.5	Diabetes with ophthalmic manifestations	250.50, 250.52
250.6	Diabetes with neurological manifestations	250.60, 250.62
250.7	Diabetes with peripheral circulatory disorders	250.70, 250.72
250.8	Diabetes with other specified manifestations	250.80, 250.82
250.9	Diabetes with unspecified complication	250.90, 250.92

Table S7. ICD-9 codes used in the extraction of the type II diabetes indicator variable.

S4 GeoSPM Software Overview

GeoSPM is implemented as a well-structured collection of MATLAB classes and packages in the "geospm" and "hdng" namespaces, preventing name-collisions with a user's existing MATLAB installation. It makes use of a separately provided SPM toolbox (synthetic_volumes_toolbox) to allow in-memory generation of SPM scan files, which we hope to integrate into SPM proper in the future. An overview of key classes and packages is shown in Figure S56. A potential user of GeoSPM invokes a single function – geospm.compute() – to initiate an analysis, passing a path to a working directory, a SpatialData object and a set of name-value options. All results will be stored as files in the given directory, including images of all regression coefficients and vector-based shape files demarking regions of significance for any applied thresholds. A SpatialData object can be constructed manually or obtained via loading a comma-separated value (CSV) file from disk via geospm.load_data(). In order to produce geo-referenced TIFF images, GeoSPM requires a SpatialData object to have an attached co-ordinate reference system. This can be specified when calling geospm.load_data() or manually, by creating a hdng.SpatialCRS object from an appropriate identifier. For example, 'EPSG:27700' is the identifier for the Ordnance Survey National Grid used by UK Biobank.

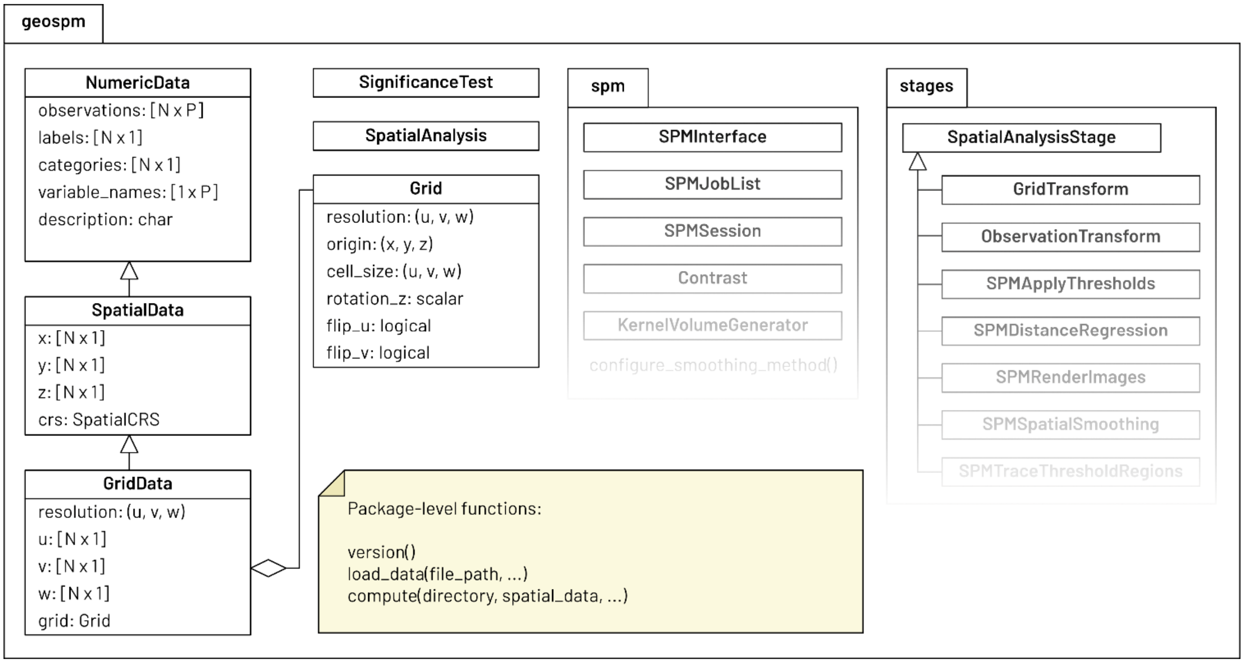

Figure S56. Class diagram of GeoSPM.

Internally, geospm.compute() uses a SpatialAnalysis object to define a pipeline comprising a number of successive processing stages, each concerned with a clearly de-lineated task, such as transforming continuous locations to discrete grid co-ordinates, rendering a Gaussian kernel of desired size at each location of the data, running SPM itself, colour-mapping output images, and extracting vector-based areas of significance for each threshold.

References

S1. Makalic, E. & Schmidt, D. F. (2016). High-dimensional Bayesian regularised regression with the BayesReg package. arXiv. DOI: 10.48550/arXiv.1611.06649.

S2. Friston, K. J., Holmes, A. P., Price, C. J., Büchel, C. & Worsley, K. J. (1999). Multisubject fMRI studies and conjunction analyses. Neuroimage *10*, 385–396. DOI: 10.1006/nimg.1999.0484.

S3. Goovaerts, P. (1997). Geostatistics for natural resources evaluation (Oxford University Press on Demand).

S4. Matérn, B. (1960). Spatial variation, Technical Report. Statens Skogsforsningsinstitut, Stockholm. https://pub.epsilon.slu.se/10033/1/medd_statens_skogsforskningsinst_049_05.pdf.

S5. Diggle, P. J. & Ribeiro, P. J. (2007). Model-based geostatistics (Springer). DOI: 10.1007/978-0-387-48536-2.

S6. Stein, M. L. (1999). Interpolation of spatial data: some theory for kriging (Springer Science & Business Media). DOI: 10.1007/978-1-4612-1494-6.
